# Development and validation of a questionnaire to assess environmental and lifestyle factors influencing infectious disease outcomes: application to SARS-CoV-2-infected individuals in Cuba

**DOI:** 10.3389/fpubh.2026.1805212

**Published:** 2026-06-29

**Authors:** Waldemar Baldoquín-Rodríguez, Ana B. Pérez, Lizette Gil, Rosario Gravier, Hector Granela, Odalys Orraca, Lillyam Betancourt-Peraza, Alejandro Almeida-Pons, Juan M. Junco-Rodriguez, Pablo Sariol, Tatiana Prieto, Dennis Pérez, Veerle Vanlerberghe, María G. Guzmán, Wim Vanden Berghe, Xaveer Van Ostade, Beatriz Sierra

**Affiliations:** 1 Center for Research, Diagnostic and Reference (CIDR), Pedro Kourí Institute of Tropical Medicine (IPK), Havana, Cuba; 2 Cellular, Molecular, and Biomedical Sciences, University of Vermont, Burlington, VT, United States; 3 Outpatient Department, Clíníca Central Cira García, Havana, Cuba; 4 Medical Attention Center, Pedro Kourí Institute of Tropical Medicine (IPK), Havana, Cuba; 5 Department of Public Health, Institute of Tropical Medicine, Antwerp, Belgium; 6 Department of Biomedical Sciences (BMW), Faculty of Pharmaceutical, Biomedical and Veterinary Sciences (FBD), Cell Death Signaling Lab, University of Antwerp, Antwerp, Belgium

**Keywords:** clinical outcome, COVID-19, environmental factors, habits, lifestyle, validation

## Abstract

**Background and objectives:**

The coronavirus disease 2019 (COVID-19) pandemic highlighted the influence of behavioral and environmental factors on the risk of infection, as well as on disease progression and severity. Therefore, it is critical to improve public health knowledge regarding the factors influencing disease outcomes. This study describes the development and validation of the “Cuban population environmental and lifestyle factors Questionnaire” (CELF-Q), a tool designed to evaluate the impact of environmental and lifestyle factors on COVID-19 outcomes in the Cuban population.

**Study design and setting:**

Based on an existing lifestyle questionnaire developed for the Latin American population, a thorough process of modification, cross-cultural adaptation, and iterative desk review was conducted to generate the CELF-Q. Pre-test analysis was performed to assess face and content validity. To evaluate content validity, a panel of 15 experts was selected. Test–retest reliability was assessed in 60 participants, while internal consistency was evaluated by administering the questionnaire to 309 individuals with SARS-CoV-2 infection. Confirmatory factor analysis (CFA) was performed to assess the fit of the CELF-Q's factor structure.

**Results:**

The content validity of the questionnaire was deemed “acceptable” across several dimensions, based on the results of the content validity ratio (CVR), content validity index (CVI), and Aiken's validity (V). Only a few items were identified for revision using these quantitative measures. Additionally, the experts supplemented the evaluations with qualitative comments for the modification of the questions. The test–retest reliability analysis showed an overall mean kappa coefficient of 0.89 [standard deviation (SD): 0.21] and a mean overall correlation coefficient of 0.99 (0.02), indicating high to almost perfect agreement. An internal consistency analysis showed that the majority of the dimensions had acceptable Cronbach's alpha values. In particular, the self-care behavior, socioeconomic restrictions, and diet and nutritional habits dimensions achieved respectable Cronbach's alpha values (between 0.7 and 0.8).

**Conclusion:**

The generated CELF-Q is a comprehensive, valid, and reliable tool for obtaining information on environmental/lifestyle factors associated with SARS-CoV-2 infection, as well as the development and severity of COVID-19, in the Cuban population.

## 1 Introduction

Risk factors commonly associated with non-communicable diseases (NCDs) are prevalent worldwide (1). Studies, primarily conducted in high-income countries (HICs) (2, 3), have shown that many of these factors also influence communicable diseases, particularly the risk of infection and severe disease outcomes (4). More recently, the coronavirus disease 2019 (COVID-19) pandemic underscored this association (2, 5). Nevertheless, the impact of these factors on emerging and re-emerging neglected tropical diseases—including arboviral, respiratory, and other infectious diseases with pandemic potential—remains comparatively understudied, particularly in lower- and middle-income countries (LMICs) situated within tropical latitudes.

Growing evidence also demonstrates that everyday choices have a significant impact on our ability to combat pathogens (6, 7). Specifically, stress, sleep deprivation, and an unhealthy diet are lifestyle factors that can weaken immune function (8).

The Cuban population exhibits distinct lifestyle patterns compared to other Latin American countries, potentially influencing health outcomes (9). The traditional Cuban diet is high in calories, fat, and sugar, with frequent consumption of refined starchy food, sugary drinks, and desserts (10). The prevalence of tobacco use and alcohol consumption is also high, contributing significantly to death and disability (11, 12).

Considering all of the above, once the first COVID-19 cases were reported in Cuba, we decided to generate and validate a questionnaire to study how habits, lifestyles, and environmental factors (potentially influencing epigenetic modifications) affect the clinical evolution of infectious diseases in the Cuban population, using SARS-Cov-2-infected individuals as a model. COVID-19 presents a spectrum of outcomes ranging from asymptomatic or subclinical infections to severe complications and death, with a notably higher incidence observed in older adults who have comorbidities (13). Nevertheless, we designed this questionnaire to assess environmental and behavioral risk factors, with a broad focus on those factors that may influence emerging and re-emerging tropical infectious diseases with epidemic potential.

In this study, we aimed to determine the validity and reliability of the questionnaire, which was created as a compilation of questions and dimensions to assess environmental and lifestyle factors reported to be relevant for individuals infected with SARS-CoV-2 and associated with COVID-19 outcomes (13–21).

## 2 Methods

### 2.1 Study design and setting

This study comprised two phases. The first phase involved the development of the Cuban population environmental and lifestyle factors questionnaire (CELF-Q), based on existing instruments such as “Cuestionario de prácticas y creencias sobre estilos de vida” and “Encuesta sobre factores contextuales relacionados con estilos de vida” (22, 23). This process involved selecting the dimensions and their corresponding items and adapting the questionnaire to the local language to ensure cultural appropriateness and ease of comprehension (Supplementary material S1).

**Figure 1:**
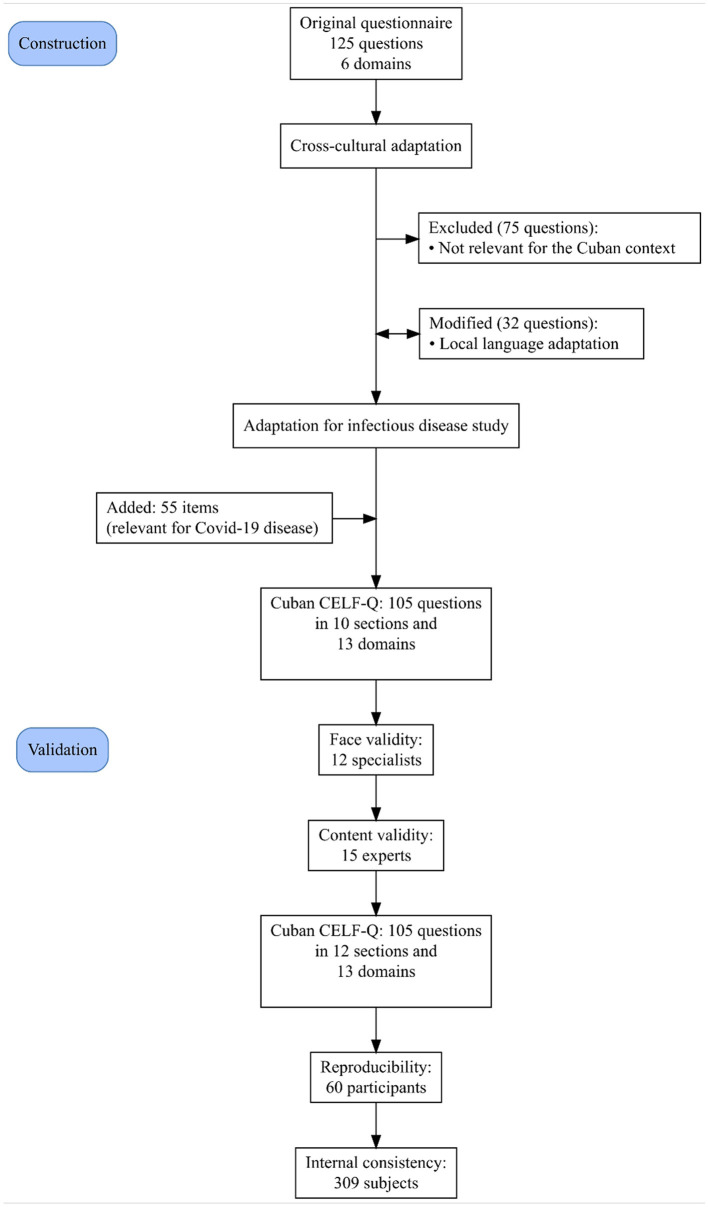
Questionnaire creation, validation, and testing.

The second phase comprised the assessment of the validity, reliability, and internal consistency of the questionnaire. The questionnaire assessed several dietary, lifestyle, and health-related behaviors, as well as risk exposures and other factors associated with SARS-CoV-2 infection and COVID-19 disease outcomes among adults infected with SARS-CoV-2 in Havana and Pinar del Río, two cities located in western Cuba. The steps are outlined in Figure 1.

### 2.2 Study population/participants

This study enrolled 309 participants (Table 1) with confirmed SARS-CoV-2 infection, as determined by reverse transcription polymerase chain reaction (RT-PCR). Participants were recruited between April 2020 and December 2023 from six hospitals in Cuba: The Pedro Kourí Institute of Tropical Medicine (IPK), the University of Informatics Sciences Hospital (UISH), Dr. Luis Días Soto Hospital, Dr. Salvador Allende Hospital, Manuel Fajardo Hospital in Havana, and the Pinar del Río Medical Science University Hospital (PRMUH).

**Table 1:** Demographic and clinical characteristics of the study participants.

Characteristic	*N* = 309^a^
Age (years)	
Mean age (SD)	51.7 (16.7)
Unknown	3
Age group	
< 65 years old	240 (78%)
65+ years old	66 (22%)
Unknown	3
Sex	
Female	142 (46%)
Male	167 (54%)
Ethnicity	
Black	49 (16%)
Mestizo	55 (18%)
White	203 (66%)
Unknown	2
Weight (Kg)	
Mean weight (SD)	75.5 (33.5)
Unknown	5
Height (m)	
Mean height	1.7 (0.1)
Unknown	8
BMI (Kg/m^2^)	
Mean BMI (SD)	27.4 (12.3)
Unknown	8
**BMI category**	
Normal	116 (39%)
Underweight	13 (4.3%)
Overweight	101 (34%)
Obese	71 (24%)
Unknown	8
Marital status	
Single	69 (23%)
Married	132 (43%)
Widowed	24 (7.9%)
Divorced	33 (11%)
In union	46 (15%)
Unknown	5
Has children	235 (76%)
Occupation	
Unemployed	21 (6.9%)
Laborer	53 (17%)
Military	12 (3.9%)
Student	13 (4.3%)
Farmer	3 (1.0%)
Housewife/homemaker	24 (7.9%)
Self-employed	25 (8.2%)
Professional	139 (46%)
Other	14 (4.6%)
Unknown	5
Healthcare professional	39 (13%)
Province/setting	
PR	100 (32%)
LH	209 (68%)
Smoking habits	56 (18%)
Comorbidities	
comorbidities, 1+	100 (32%)
comorbidities, 2+	22 (7.1%)
comorbidities, 3+	6 (1.9%)
Diabetes mellitus	39 (13%)
Asthma	36 (12%)
Hypertension	117 (38%)
Dyslipidemia/hypercholesterolemia	4 (1.3%)
Cancer	6 (1.9%)
Allergies	27 (8.7%)
Disease severity	
Asymptomatic	98 (32%)
Mild/Moderate	107 (35%)
Severe	104 (34%)

^a^Mean (SD); *n* (%).

In accordance with the protocols approved by a commission of the Cuban Ministry of Public Health (MINSAP) in April 2020 (24), all individuals who tested positive for SARS-CoV-2 by RT-PCR were hospitalized in different institutions across the country. These institutions were prepared to adhere to established biosafety protocols for the management of COVID-19 cases.

### 2.3 Development of the CELF-Q

#### 2.3.1 Cross-cultural adaptation and development of the CELF-Q

The CELF-Q was based on the questionnaire developed and validated by Arrivillaga et al. (22) and Arrivillaga and Salazar (23), which characterizes health-related beliefs and their relationship with protective (or risk) practices among young university students in Cali, Colombia (22, 23). After receiving approval from the original instrument authors, an iterative process of modification and cross-cultural adaptation was conducted. This involved desk reviews and expert evaluations. The adapted questionnaire underwent several revisions to ensure alignment with its intended purpose, content validity, structural coherence, contextual relevance, and adherence to ethical considerations. Each item was evaluated for readability, clarity, and accuracy, with a focus on reflecting the influence of lifestyle behaviors and environmental factors on COVID-19 symptomatology and severity. As a result, the first version of the CELF-Q was created, initially consisting of 105 questions distributed across 10 sections (demographics, health-related factors, physical activity, free-time activities, self-care, practices, diet, psychoactive substance use, sleep quality, and socioeconomic status), covering 13 domains.

#### 2.3.2 Face and content validity

To assess face validity, a printed version of the questionnaire was distributed to 12 staff members in the Virology Department at the IPK. Participants were asked to complete the questionnaire, paying special attention to its usability, readability, comprehensiveness of the questions and response options, duration, and clarity.

An expert committee was selected using the snowball method to evaluate the content validity of the questionnaire. The panel comprised fifteen experts, including one biostatistician, one economist, one computer specialist, one pharmacologist, two immunologists/virologists, two clinicians/infectologists, two biochemists/pharmacologists, two epidemiologists, two sociologists, and one psychiatrist.

A new assessment tool (Supplementary material S2) was developed for evaluating individual questions and the overall questionnaire. As suggested elsewhere (25–27), experts were provided with (1) an introductory letter explaining the objectives of the research, a description of the instrument, and the criteria for content validity; (2) content validation assessment forms; and (3) a copy of the CELF-Q. The experts conducted a review of the 105-question instrument independently.

The criteria used to assess the content validity of the items were as follows: (1) pertinence (are the questions in the questionnaire relevant to the research objective and can they be adequately answered by the provided options?), (2) adequacy (is the questionnaire adapted to the characteristics of the respondents, and is it formulated in clear and appropriate language?), and (3) organization (is there a logical structure in the sequence of questions within the questionnaire?). Experts evaluated the questions based on these three criteria using a five-point scale, where 1 indicated a low level of validity and 5 indicated a high level.

The quantitative analysis of content validity included measures such as Aiken's validity (V) (28), Lawshe's content validity ratio (CVR) (29), and the item-level content validity index (I-CVI), as described elsewhere (25). Aiken's V index, which evaluates the degree of agreement between experts, was computed using the following formula (Equation 1):


$$\begin{array}{l} V=\frac{\sum s}{n\left(c-1\right)} \end{array}$$ (1)

Where *s* = *r*−*lo*, *lo* is the lowest validity assessment score, *c* is the highest validity assessment score, and *r* is the score given by the raters.

Aiken's V values range from 0 (total disagreement) to 1 (total agreement). A value of 0.5 is regarded as indicating indecision. The 95% confidence interval for Aiken's V index was computed in *R* using the method proposed by Penfield and Giacobbi (30). The null hypothesis was defined as *V* ≤ 0.50. Critical values for *V* were also provided by Aiken (28) based on the number of rating categories and the number of experts involved. In our study, which used a 5-point rating scale and included 15 experts (with an alpha level of *p* = 0.05), items with an Aiken's *V* value greater than or equal to 0.67 were considered valid (28).

Lawshe's CVR was estimated using the Equation 2 shown below:


$$\begin{array}{l} CVR=\frac{2n_{e}}{n}-1 \end{array}$$ (2)

Where *ne* = number of experts who rated the item as essential and *n* = total number of experts who evaluated the item.

CVR values range between 1 (perfect agreement) and −1 (perfect disagreement). A value of 0 indicates that half of the panel experts agree that an item is essential. A table of critical CVR values based on the number of experts was provided by Lawshe (29). For our panel of 15 experts, the minimum acceptable value of CVR (at a significance level of α = 0.05) is 0.49 (29).

The I-CVI is calculated (Equation 3) as the proportion of experts who rated an item with a score of 4 or 5:


$$\begin{array}{l} ICVI=\frac{n_{e}}{n} \end{array}$$ (3)

A minimum I-CVI value of 70% has been recommended for an item to be considered valid. Items with I-CVI values between 70 and 79% should be revised, while items with I-CVI values greater than 79% are considered appropriate (25).

Content validity for each dimension scale was calculated by taking the mean of the I-CVI for all items included in each scale (31). Davis suggested that the developed scales should have a content validity index (CVI) of 0.80 to be considered acceptable (32). Scale-level mean values of the CVR and Aiken's *V* index were also computed.

The expert assessment tool also included a section in which the experts were asked to provide qualitative comments for each item, each dimension, and the overall questionnaire. A qualitative review of the expert's comments and suggestions was conducted to evaluate the questionnaire's appropriateness for the Cuban population, the relevance of its content, and the identification of any misunderstandings or omissions. Based on the expert's evaluation, a reorganization of the CELF-Q items was proposed and implemented, aligning them with current literature and existing validated instruments specific to each domain.

#### 2.3.3 Variables and measurements

After reorganizing the CELF-Q items, two sections were added: one to group the items exploring pollution sources and another for questions regarding COVID-19 clinical symptoms, signs, and evolution. Finally, following a detailed process of validation, the questionnaire was developed, and it consisted of 12 sections with 105 questions. It covers 13 construct dimensions and contains 244 items (28 numeric and 216 categorical items; Supplementary material S1). This questionnaire covers 18 health-related categories, including demographics (12 items), diet/nutritional habits (49), physical activity (8), healthy free-time activities (12), self-care habits (19), consumption of medicines (3), consumption of natural products (5), consumption of psychoactive products (6), economic restrictions (44), exposure to stress and violence (7), sleep quality (6), work environment risks (6), pollution (11), sedentary behavior (2), comorbidities (8), general family information (5), medical history (19), and COVID-19 clinical presentation (22). Based on the CELF-Q, all data were self-reported by participants during one-to-one interviews conducted by qualified health personnel to reduce memory bias. Out-of-range variables were recoded as missing. Dichotomous yes (1)/no (2) questions were recoded as 1/0, and the category coded as 0 was used as the reference in the regression models.

#### 2.3.4 Confirmatory factor analysis

The factor structure of the original questionnaire (23) consisted of six dimensions: physical activity and sports; leisure/free-time activities; self-care and medical care; diet and eating habits; consumption of alcohol, tobacco, and drugs; and sleep quality. To evaluate the version adapted for the Cuban context (CELF-Q), which includes 13 dimensions, we conducted a confirmatory factor analysis (CFA).

Before conducting the CFA, the Kaiser–Meyer–Olkin (KMO) measure of sampling adequacy (MSA) and Bartlett's test of sphericity (33) were used to evaluate the suitability of the data for factor analysis (Supplementary Table S4.1). The MSA quantifies the common variance as a proportion of the total variance. Overall MSA values of 0.5 or higher were considered acceptable (34). The MSA was also calculated for each item. Bartlett's test was used to rule out that the items within each dimension are uncorrelated. This test examines whether the correlation matrix is an identity matrix, implying that all off-diagonal correlations are equal to zero. Rejecting the null hypothesis indicates that the items are correlated, suggesting that the data are suitable for factor analysis.

A diagonally weighted least squares (DWLS) estimation method with delta parameterization was used to address the dichotomous and ordinal nature of the scale items (35). The analyses were conducted in R using the lavaan package (36, 37). The average variance extracted (AVE) was computed to evaluate the amount of variance captured by each dimension. Latent constructs explaining more than 50% of the indicator variance were considered adequate (38).

The hypothesized model structure was evaluated using χ^2^ statistics and goodness-of-fit indices, including the Tucker–Lewis index (TLI) (39), the comparative fit index (CLI) (40), the root mean square error of approximation (RMSEA), and the standardized root mean squared residual (SRMR). TLI and CLI values above 0.90 were considered acceptable. RMSEA and SRMR values greater than 0.10 indicated poor model fit (41).

#### 2.3.5 Reproducibility and internal consistency assessment

*Test–retest reliability* was assessed by administering the questionnaire to the same 60 participants, using the same interviewers, and comparing the first and second administrations 1 week apart. The objective of this assessment was to determine the level of agreement between the two time points. Different reliability measures were employed according to the type of question. For categorical items, Cohen's kappa coefficients were calculated to quantify agreement. Ordinal items were analyzed using Fleiss's kappa, while either Pearson's or Spearman's correlation coefficients were used for numeric questions, based on the distribution of the variable. Pearson's *r* and Spearman's rho range from 0 to 1, with 0 indicating no agreement between two questions for the same person and 1 indicating perfect agreement (42). Items exhibiting a kappa coefficient or rho greater than 0.6 were considered valid. Intraclass correlation coefficients (ICCs) were also used for numeric questions to assess reliability.

To account for selection bias, the demographic characteristics of the validation group (*n* = 60) were compared with those of the remaining study participants (*n* = 249) using Pearson's chi-squared tests (or Fisher's exact test) for categorical variables and the Wilcoxon rank-sum test for continuous variables (Table 2). Compared to the remaining participants, the validation group included a significantly higher proportion of female participants. Black ethnicity was the second most prevalent category, followed by mestizo ethnicity. Furthermore, the validation group exhibited statistically significantly lower weight and BMI. These differences were not expected to influence the results of the questionnaire validation.

**Table 2:** Demographic and clinical characteristics of the participants included in the validation group compared to the remaining group.

Characteristic	Overall	Validation group	Remaining group	*P*-value^b^
	*N* = 309^a^	*N* = 60^a^	*N* = 249^a^	
Sex				<0.001
Female	142 (46%)	39 (65%)	103 (41%)	
Male	167 (54%)	21 (35%)	146 (59%)	
Ethnicity				0.028
Black	49 (16%)	13 (22%)	36 (15%)	
Mestizo	55 (18%)	4 (6.7%)	51 (21%)	
White	203 (66%)	43 (72%)	160 (65%)	
Unknown	2	0	2	
Age (years)				
Mean age	51.7 (16.7)	52.3 (17.3)	51.6 (16.6)	0.7
Unknown	3	0	3	
Weight (Kg)				
Mean weight	75.5 (33.5)	69.2 (12.2)	77.0 (36.7)	0.029
Unknown	5	0	5	
Height (m)				
Mean height	1.67 (0.11)	1.66 (0.09)	1.67 (0.11)	0.4
Unknown	8	0	8	
BMI (Kg/m^2^)				
Mean BMI	27.4 (12.3)	25.1 (4.3)	27.9 (13.6)	0.032
Unknown	8	0	8	
BMI category				0.021
Normal	116 (39%)	30 (50%)	86 (36%)	
Underweight	13 (4.3%)	3 (5.0%)	10 (4.1%)	
Overweight	101 (34%)	21 (35%)	80 (33%)	
Obese	71 (24%)	6 (10%)	65 (27%)	
Unknown	8	0	8	
Marital status				0.7
Single	69 (23%)	13 (22%)	56 (23%)	
Married	132 (43%)	25 (42%)	107 (44%)	
Widowed	24 (7.9%)	3 (5.1%)	21 (8.6%)	
Divorced	33 (11%)	6 (10%)	27 (11%)	
In union	46 (15%)	12 (20%)	34 (14%)	
Unknown	5	1	4	
Occupation				
Unemployed	21 (6.9%)	0 (0%)	21 (8.6%)	
Laborer	53 (17%)	18 (30%)	35 (14%)	
Military	12 (3.9%)	2 (3.3%)	10 (4.1%)	
Student	13 (4.3%)	3 (5.0%)	10 (4.1%)	
Farmer	3 (1.0%)	0 (0%)	3 (1.2%)	
Housewife/homemaker	24 (7.9%)	8 (13%)	16 (6.6%)	
Self-employed	25 (8.2%)	5 (8.3%)	20 (8.2%)	
Professional	139 (46%)	24 (40%)	115 (47%)	
Other	14 (4.6%)	0 (0%)	14 (5.7%)	
Unknown	5	0	5	
Healthcare professional	39 (13%)	7 (12%)	32 (13%)	0.8
Smoking_hb	56 (18%)	5 (8.3%)	51 (20%)	0.028
Comorbidities				
Diabetes mellitus	39 (13%)	5 (8.3%)	34 (14%)	0.3
Asthma	36 (12%)	6 (10%)	30 (12%)	0.7
Hypertension	117 (38%)	20 (33%)	97 (39%)	0.4
Dyslipidemia/ hypercholesterolemia	4 (1.3%)	2 (3.3%)	2 (0.8%)	0.2
Cancer	6 (1.9%)	1 (1.7%)	5 (2.0%)	>0.9
Allergies	27 (8.7%)	13 (22%)	14 (5.6%)	< 0.001

^a^*n* (%); Mean (SD).

^b^Pearson's Chi-squared test; Wilcoxon rank sum test; Fisher's exact test; NA.

*Internal consistency* was assessed using Cronbach's alpha, applied to the CELF-Q administered to the 309 individuals with confirmed SARS-CoV-2 infection. Alpha coefficients were calculated for each subscale, and, for comparison with the original questionnaire, an overall value was computed by including the items covered by 13 selected subscales: diet, physical activity, free-time activities, self-care, consumption of medicines, consumption of natural products, consumption of psychoactive products, socioeconomic restrictions, exposure to stress and violence, sleep quality, work environmental risks, pollution, and sedentary behavior. Cronbach's alpha values range from 0 to 1. Alpha coefficients were classified using the categories proposed by DeVellis and Thorpe (43), who considered values below 0.60 as unacceptable, between 0.60 and 0.65 as undesirable, between 0.65 and 0.70 as minimally acceptable, between 0.70 and 0.80 as respectable, and between 0.80 and 0.90 as very good (44).

### 2.4 Statistical analysis

Data were tabulated as mean and standard deviation (SD) for continuous variables with a normal distribution. Quantitative variables that did not follow a normal distribution were summarized using the median and interquartile range (IQR). Absolute and relative frequencies (proportions) were used for categorical variables. The Shapiro–Wilk test of normality was used to determine whether the examined variables followed a normal distribution. Student's *t*-test was used for continuous variables that were normally distributed. The chi-squared test was used for categorical variables. A two-tailed *p*-value of < 0.05 was considered to indicate a statistically significant difference at the 95% confidence level. Analyses were performed using R statistical software, version 4.0.1 (45).

### 2.5 Ethical concerns

The study was conducted in accordance with the Declaration of Helsinki (2013). The protocol was approved by the Scientific and the Ethics Committee of the IPK, the University of Informatic Sciences Hospital (UISH), and the Pinar del Río Medical Science University Hospital (PRMUH), Cuba. Written informed consent was obtained from all respondents following an explanation of the study's aims and procedures. The experts involved in the questionnaire assessment were also required to sign an informed consent form. This study was also approved by and registered with the Cuban Ministry of Public Health (code number 2105028) in accordance with the regulatory Cuban agency (CECMED).

## 3 Results

### 3.1 Cross-cultural adaptation and the development of the CELF-Q

The questionnaire developed by Arrivillaga et al. for the Colombian population (22, 23) was originally in the Spanish language, and the relationship between the questionnaire and its underlying constructs was preserved in both the original and the adapted versions. Nevertheless, extensive modifications based on experts' opinions were made (Figure 1). From the original questionnaire, 75 items considered irrelevant to the Cuban context were excluded, whereas 32 items were modified to make them semantically, idiomatically, and culturally comprehensible and relevant for the Cuban population. A total of 55 items were further added, including questions designed to obtain information on COVID-19 disease.

After cross-cultural and COVID-19 adaptation, the resulting questionnaire was titled the CELF-Q (Supplementary material S1).

### 3.2 Application of the CELF-Q to Cuban COVID-19 patients

Between April 2020 and December 2023, the CELF-Q was administered to 309 SARS-CoV-2-positive participants. Table 1 shows the characteristics of the included participants. The mean age was 51.7 years (SD = 16.7); 167 participants (54%) were male; and 203 participants (66%) were White, 55 (18%) were mestizo, and 49 (16%) were Black. The mean weight was 75.5 kg (SD = 33.5), and the BMI was 27.4 kg/m^2^ (SD = 12.3).

Regarding comorbidities, hypertension, diabetes mellitus, bronchial asthma, and allergies were present in 117 (38%), 39 (13%), 36 (12%), and 27 (8.7%) participants, respectively. Common risk factors such as obesity and smoking were also reported in 24% and 18% of participants, respectively. In total, 98 participants (32%) had asymptomatic SARS-CoV-2 infection, while 104 (34%) progressed to severe disease.

### 3.3 Face and content validity

All 12 workers interviewed for the face validity assessment found the questionnaire to be clear, readable, and comprehensible. In addition, the participants reported that the questionnaire items were acceptable and had an appropriate emotional impact. On average, completion of the questionnaire took 45 min., which was also considered acceptable by the respondents. Questions belonging to the construct of health beliefs of the original questionnaire were excluded. Questions related to practices were reviewed for modification or removal. A total of six questions were removed from the self-care section, five from the demographics and alcohol, tobacco, and other drug consumption sections, and four from the sleep quality and free-time activities sections.

Of the 32 modified questions, 12 belonged to the diet and nutritional habits section, followed by the self-care section with seven questions. The physical activity and alcohol, tobacco, and other drug consumption sections each included four modified questions. Most of the added questions were incorporated into three new sections: health history (18 questions), health practices (14 questions), and socioeconomic characteristics (10 questions). We decided to use the International System of Units for weight and height measurements. The starting age for cervical cancer screening was set at 25 years, while the starting age for prostate cancer screening was established at 40 years. To avoid confusion, condiments were reclassified into three categories: natural condiments, dehydrated condiments, and condiments containing chemical preservatives.

The content validity of the CELF-Q was based on the evaluations of 15 experts. Table 3 shows the average validity for each dimension—pertinence, adequacy, and organization—assessed using the CVI, CVR, and Aiken's *V*. The validity values for each item are provided in Supplementary Table S3.1 in Supplementary material S3.

**Table 3:** Content validity per dimension: average values (min; max) of the CVR, the S-CVI, and Aiken's V for pertinence (p), adequacy (a), and organization (o).

Dimensions^a^	cvr_p	scvi_p	V_p	cvr_a	scvi_a	V_a	cvr_o	scvi_o	V_o
Demg	0.89 (0.33;1.00)	0.94 (0.67;1.00)	0.94 (0.75;1.00)	0.90 (0.60;1.00)	0.95 (0.80;1.00)	0.95 (0.85;1.00)	0.89 (0.60;1.00)	0.94 (0.80;1.00)	0.96 (0.87;1.00)
Diet	0.88 (0.73;1.00)	0.94 (0.87;1.00)	0.95 (0.87;1.00)	0.98 (0.87;1.00)	0.99 (0.93;1.00)	0.98 (0.95;1.00)	1.00 (1.00;1.00)	1.00 (1.00;1.00)	0.99 (0.97;1.00)
Phys	0.66 (0.60;0.73)	0.83 (0.80;0.87)	0.88 (0.85;0.90)	0.87 (0.73;1.00)	0.93 (0.87;1.00)	0.92 (0.85;0.97)	0.97 (0.87;1.00)	0.98 (0.93;1.00)	0.96 (0.92;1.00)
FreeT	0.87 (0.87;0.87)	0.93 (0.93;0.93)	0.96 (0.95;0.97)	1.00 (1.00;1.00)	1.00 (1.00;1.00)	0.98 (0.97;0.98)	1.00 (1.00;1.00)	1.00 (1.00;1.00)	0.98 (0.97;0.98)
SelfC	0.85 (0.60;1.00)	0.92 (0.80;1.00)	0.94 (0.87;1.00)	0.90 (0.47;1.00)	0.95 (0.73;1.00)	0.94 (0.80;1.00)	0.93 (0.73;1.00)	0.97 (0.87;1.00)	0.96 (0.87;0.98)
CsMed	0.78 (0.73;0.87)	0.89 (0.87;0.93)	0.91 (0.90;0.92)	0.73 (0.60;0.87)	0.87 (0.80;0.93)	0.90 (0.85;0.95)	0.96 (0.87;1.00)	0.98 (0.93;1.00)	0.96 (0.95;0.97)
CsNat	0.87 (0.87;0.87)	0.93 (0.93;0.93)	0.94 (0.93;0.95)	1.00 (1.00;1.00)	1.00 (1.00;1.00)	0.96 (0.95;0.97)	1.00 (1.00;1.00)	1.00 (1.00;1.00)	0.98 (0.98;0.98)
CsPsc	0.94 (0.87;1.00)	0.97 (0.93;1.00)	0.98 (0.97;1.00)	0.91 (0.87;1.00)	0.96 (0.93;1.00)	0.95 (0.93;0.98)	0.98 (0.87;1.00)	0.99 (0.93;1.00)	0.98 (0.97;1.00)
EconR	0.92 (0.60;1.00)	0.96 (0.80;1.00)	0.95 (0.83;1.00)	0.92 (0.73;1.00)	0.96 (0.87;1.00)	0.96 (0.88;1.00)	0.98 (0.87;1.00)	0.99 (0.93;1.00)	0.99 (0.95;1.00)
StrsV	0.91 (0.87;1.00)	0.95 (0.93;1.00)	0.97 (0.95;0.98)	0.96 (0.87;1.00)	0.98 (0.93;1.00)	0.95 (0.93;0.98)	0.89 (0.73;1.00)	0.94 (0.87;1.00)	0.94 (0.88;0.97)
Sleep	0.93 (0.73;1.00)	0.97 (0.87;1.00)	0.96 (0.88;1.00)	1.00 (1.00;1.00)	1.00 (1.00;1.00)	0.99 (0.97;1.00)	1.00 (1.00;1.00)	1.00 (1.00;1.00)	1.00 (0.98;1.00)
WrkEn	0.87 (0.87;0.87)	0.93 (0.93;0.93)	0.95 (0.92;0.97)	0.92 (0.73;1.00)	0.96 (0.87;1.00)	0.94 (0.87;0.98)	0.97 (0.87;1.00)	0.99 (0.93;1.00)	0.97 (0.95;0.98)
Pollt	0.87 (0.73;1.00)	0.93 (0.87;1.00)	0.94 (0.87;0.98)	0.87 (0.73;1.00)	0.93 (0.87;1.00)	0.92 (0.88;0.95)	0.90 (0.87;1.00)	0.95 (0.93;1.00)	0.95 (0.90;0.98)
Sednt	0.87 (0.87;0.87)	0.93 (0.93;0.93)	0.94 (0.93;0.95)	1.00 (1.00;1.00)	1.00 (1.00;1.00)	0.95 (0.95;0.95)	0.94 (0.87;1.00)	0.97 (0.93;1.00)	0.94 (0.93;0.95)
CCI	0.84 (0.60;1.00)	0.92 (0.80;1.00)	0.93 (0.85;0.98)	0.88 (0.60;1.00)	0.94 (0.80;1.00)	0.93 (0.83;0.95)	0.79 (0.60;1.00)	0.90 (0.80;1.00)	0.92 (0.82;0.98)
outc	0.96 (0.87;1.00)	0.98 (0.93;1.00)	0.97 (0.95;1.00)	0.78 (0.73;0.87)	0.89 (0.87;0.93)	0.89 (0.87;0.93)	0.91 (0.87;1.00)	0.96 (0.93;1.00)	0.96 (0.93;1.00)

^a^Demg, demographics; Diet, negative nutritional habits; Phys, physical activity; FreeT, healthy free-time activities; Selfc, self-care; CsMed, excessive consumption of medicines; CsNat, consumption of natural products, CPsych, consumption of psychoactive products; EconR, economic restrictions; StrsV, exposure to stress and violence; Sleep, sleep quality; WrkEn, work environment risks; Pollt, pollution; Sednt - sedentary behavior; CCI, comorbidities; outc, outcomes.

For each dimension, the average CVR for pertinence (CVR_p_), adequacy (CVR_a_), and organization (CVR_o_) exceeded the critical value of 0.49, as suggested for a panel of 15 experts (29). Nevertheless, among the 11 items in the demographics section, one item (Item 10: Were you living in this place during 2018?/¿Usted también vivía en este lugar durante el año 2018?) had a CVR_p_ value of 0.33 and was revised. Moreover, the self-care dimension, which achieved a CVR for adequacy (CVR_a_) of 0.9, included one item (out of 14) with a CVR_a_ of 0.47 (Item 49: How many hours do you work in an average working day?/¿Cuántas horas diarias trabajas en un día laboral en promedio?), suggesting that it required revision.

All dimension-level S-CVI values for pertinence (S-CVI_p_), adequacy (S-CVI_a_), and organization (S-CVI_o_) exceeded the recommended value of 80%. Nevertheless, in the demographics section, one item (Item 10) had an S-CVI_p_ value of 0.67 and was revised. In addition, all dimension-level Aiken's *V* values exceeded the recommended threshold of 0.67. Item-wise analysis also showed that all items exceeded this threshold.

Based on the content validity ratio (CVR), content validity indices (I-CVI and S-CVI), and Aiken's *V*, the questionnaire demonstrated overall acceptable content validity. The expert committee evaluating the content validity of the questionnaire concluded that the items were comprehensible and appropriate for the local context.

### 3.4 Reproducibility

The test–retest reliability analysis showed an overall mean kappa coefficient of 0.89 (standard deviation 0.21) for all 198 categorical items included in the evaluation dataset, indicating a high level of agreement (Table 4). Cohen's kappa showed similar results, with a mean of 0.88 (0.22) for the 178 items analyzed (Table 5). However, minimal values below 0.1 in the diet, self-care, consumption of medicines, and general aspects sections indicated substantial variability across questionnaire dimensions. A mean overall Fleiss's kappa coefficient of 0.97 (0.04) was obtained for all 20 ordinal items, ranging from a mean of 0.91 (0.002) for the free-time activities section to 1.0 for the diet, consumption of psychoactive substances, and sleep quality sections, indicating high to almost perfect agreement. A mean overall correlation coefficient of 0.99 (0.02) was obtained for all 22 numerical items, ranging from a mean of 0.93 for diet-related items to 1.00 (0.00) for demographic items (Supplementary Table S3.2). Consistent with the correlation coefficients, the ICC showed a mean value of 0.99 (0.02), indicating almost perfect agreement. Due to variation between and within dimensions, a more detailed analysis will be presented for the relevant dimensions and items.

**Table 4:** Reliability tests per dimension scale: mean (SD)(min;max) and number of variables included for kappa coefficient (Cohen's kappa or Fleiss kappa), correlation coefficient (Pearson's correlation coefficient or Spearman's rho), and ICCs.

Dimensions^a^	Kappa^b^	ICC^c^	*R^d^*
Demog	0.95(0.09) (0.76;1.00):8	1.00(5e-05) (1.00;1.00):4	1.00(6e-05) (1.00;1.00):4
Diet	0.82(0.30) (−0.03;1.00):48	0.92(NA) (0.92;0.92):1	0.93(NA) (0.93;0.93):1
Phys	0.91(0.12) (0.66;1.00):7	–	–
FreeT	0.93(0.07) (0.80;1.00):12	–	–
Selfc	0.83(0.23) (−0.02;1.00):19	–	–
CsMed	0.61(0.47) (0.07;0.89):3	–	–
CsNat	0.98(0.04) (0.91;1.00):4	–	–
CPsych	0.98(0.02) (0.95;1.00):5	–	–
EconR	0.97(0.04) (0.87;1.00):18	1.00(0.01) (0.95;1.00):17	0.99(0.02) (0.93;1.00):17
StrsV	0.97(0.06) (0.83;1.00):7	–	–
Sleep	0.82(0.11) (0.66;1.00):6	–	–
WrkEn	0.86(0.22) (0.48;1.00):5	–	–
Pollt	0.95(0.08) (0.79;1.00):9	–	–
Sednt	0.97(0.04) (0.95;1.00):2	–	–
CCI	0.94(0.07) (0.84;1.00):8	–	–
Health	0.86(0.27) (0.00;1.00):17	–	–
CVD_CP	0.94(0.06) (0.83;1.00):15	–	–
General	0.99(0.02) (0.96;1.00):5	–	–
Overall	0.89(0.21) (−0.03;1.00):198	0.99(0.02) (0.92;1.00):22	0.99(0.02) (0.93;1.00):22

^a^Demog, Demographic; Diet, negative nutritional habits; Phys, Physical activity; FreeT, healthy free time activities; Selfc, self care; CsMed, Excessive consumption of medicines; CsNat, consumption of natural products; CPsych, consumption of psychoactive products; EconR, economic restrictions; StrsV, exposure to stress and violence; Sleep, sleep quality; WrkEn, work environment risks; Pollt, pollution; Sednt, sedentary behaviour; CCI, comorbidities; General, other convenience variables; Health, health related variable; CVD_CP, COVID-19 clinical picture (symptoms and signs); Overall, all included variables.

^b^Cohen's kappa coefficients were calculated for categorical variables. Fleiss's kappa coefficients were calculated for ordinal variables.

^c^ICC-Intraclass correlation coefficient were calculated for numeric variables.

^d^Pearson's product-moment correlation coefficient (*R*) or Spearman's rank correlation (rho) were calculated for numeric variables, depending on variable distribution. Shapiro test was used for assessing normality.

**Table 5:** Reliability tests per dimension scale: mean(SD)(min;max) and number of variables included for Cohen's kappa, Fleiss's kappa, ICC, Pearson's correlation coefficient, and Spearman's rho correlation coefficient.

Dimensions^a^	Cohens kappa^b^	Fleiss kappa^c^	ICC^d^	Pearson's R^e^	Spearman's rho^f^
Demog	0.95(0.09) (0.76;1.00):8	–	1.00(5e-05) (1.00;1.00):4	1.00(6e-05) (1.00;1.00):3	1.00(NA) (1.00;1.00):1
Diet	0.82(0.31) (−0.03;1.00):46	1.00(0.00) (1.00;1.00):2	0.92(NA) (0.92;0.92):1	–	0.93(NA) (0.93;0.93):1
Phys	0.89(0.16) (0.66;1.00):4	0.94(0.06) (0.88;1.00):3	–	–	–
FreeT	0.93(0.07) (0.80;1.00):10	0.91(0.002) (0.91;0.91):2	–	–	–
Selfc	0.83(0.23) (−0.02;1.00):19	–	–	–	–
CsMed	0.61(0.47) (0.07;0.89):3	–	–	–	–
CsNat	0.98(0.04) (0.91;1.00):4	–	–	–	–
CPsych	0.98(0.03) (0.95;1.00):4	1.00(NA) (1.00;1.00):1	–	–	–
EconR	0.97(0.05) (0.87;1.00):15	0.97(0.03) (0.94;1.00):3	1.00(0.01) (0.95;1.00):17	–	0.99(0.02) (0.93;1.00):17
StrsV	0.97(0.08) (0.83;1.00):5	0.99(0.02) (0.97;1.00):2	–	–	–
Sleep	0.78(0.08) (0.66;0.85):5	1.00(NA) (1.00;1.00):1	–	–	–
WrkEn	0.79(0.27) (0.48;1.00):3	0.97(0.04) (0.94;1.00):2	–	–	–
Pollt	0.95(0.08) (0.79;1.00):9	–	–	–	–
Sednt	–	0.97(0.04) (0.95;1.00):2	–	–	–
CCI	0.94(0.07) (0.84;1.00):8	–	–	–	–
Health	0.85(0.28) (0.00;1.00):16	0.95(NA) (0.95;0.95):1	–	–	–
CVD_CP	0.94(0.06) (0.83;1.00):15	–	–	–	–
General	0.99(0.02) (0.96;1.00):4	1.00(NA) (1.00;1.00):1	–	–	–
Overall	0.88(0.22) (−0.03;1.00):178	0.97(0.04) (0.88;1.00):20	0.99(0.02) (0.92;1.00):22	1.00(6e-05) (1.00;1.00):3	0.99(0.02) (0.93;1.00):19

^a^Demog, Demographic; Diet, negative nutritional habits; Phys, Physical activity; FreeT, healthy free time activities; Selfc, self care; CsMed, Excessive consumption of medicines; CsNat, consumption of natural products; CPsych, consumption of psychoactive products; EconR, economic restrictions; StrsV, exposure to stress and violence; Sleep, sleep quality; WrkEn, work environment risks; Pollt, pollution; Sednt, sedentary behaviour; CCI, comorbidities; General, other convenience variables; Health, health related variable; CVD_CP, COVID-19 clinical picture (symptoms and signs); Overall, all included variables.

^b^Cohen's kappa coefficients were calculated for categorical variables.

^c^Fleiss's kappa coefficients were calculated for ordinal variables.

^d^ICC-Intraclass correlation coefficient were calculated for numeric variables.

^e^Pearson's product-moment correlation coefficients (*R*) were calculated for numeric variables with normal distribution.

^f^Spearman's rank correlation (rho) were calculated for numeric variables with non-normal distribution. Shapiro test was used for assessing normality.

In the demographic dimension, eight categorical and four numerical items were included. With a mean Cohen's kappa coefficient of 0.95 (ranging from 0.76 to 1.00) for categorical variables and a mean correlation of 1.00 (0.84–1.00) for continuous variables, a high level of agreement was observed. In the diet and nutritional habits domain, a mean Cohen's kappa of 0.82 (0.03–1) was obtained for all 46 items. Fleiss's kappa for two ordinal variables was 1.00 (0.0). Items with low values were revised for ambiguous meaning: eating dinner (0.0), the use of dehydrated condiments/condimentos deshidratados (0.0), any condiments/condimentos (−0.029), consumption of chicken meat/ tipo_carne_pollo (−0.023), and consumption of meat at least once per week/1vs_carne (0.0). Only one continuous variable was included in this dimension and displayed a high correlation of 0.93.

Low agreement was observed in three items across different dimensions. Specifically, the “take vehicle with driver under alcohol effect” item in the self-care dimension (kappa = −0.017), the “consumption of medicines” question in the consumption of medicines dimension (kappa = 0.068), and the “acute medical conditions in the last month” item in the general dimension (kappa = 0.00) exhibited poor agreement.

### 3.5 Confirmatory factor analysis

Before conducting CFA, items with correlations approximately equal to 1 were revised to remove unnecessary duplicates. In addition, one question (consumption of more than three alcohol drinks per week) with more than 30% missing values was also removed.

The dimensions considered in this study included between 2 and 33 items. The KMO test was used to evaluate the adequacy, for factor analysis, of the items included in each dimension (Supplementary Table S4.1). The 13 dimensions exhibited MSA values equal to or above 0.5. In addition, Bartlett's test was significant for 12 of the 13 dimensions. The exception was sedentary behavior [$\chi_{\left(df:1\right)}^{2}=0.047$, *p* = 0.828], a dimension consisting of only two items.

Considering the large number of items and latent factors in the CELF-Q, and the relatively small sample size (*N* = 309), separate unidimensional confirmatory factor analyses were performed. In these analyses, the items included in each dimension loaded onto a single factor representing the dimension. This approach, using several unidimensional models instead of one large first-order, 13-factor model, helped identify potential issues leading to convergence problems within the constraints of the sample size (Supplementary material S4).

The majority of the models converged between 2 and 74 iterations. Only two dimensions required more than 50 iterations: consumption of natural products and sleep quality.

Fit measures were reliably computed for 11 of the 13 dimensions, as these dimensions contained more than three items (df > 0). The exceptions were the two dimensions (of 13) that included three or fewer items: sedentary behavior and consumption of medicines (Supplementary Table S4.2).

Good fit values were observed for the consumption of natural products and sleep quality dimensions, which were supported by agreement across all indicators (χ^2^ *p* > 0.05, CFI > 0.95, TLI > 0.95, RMSEA < 0.1, and SRMR < 0.1).

Partial agreement among the fit indices was observed for the physical activity, consumption of psychoactive substances, pollution, self-care behavior, exposure to stress and violence, and work environment risks dimensions. For these dimensions, the CFI and TLI were above 0.95, while the RMSEA was below 0.1. Nevertheless, the χ^2^ *p*-value below 0.05 and the SRMR > 0.1 suggest that there is still room for model improvement.

Finally, the diet quality, economic deprivation, and free-time activities dimensions showed poor fit values, suggesting that a more thorough revision of the items included in these dimensions is required.

The amount of variance captured by each dimension was also evaluated (Supplementary Table S4.3). In total, five of 13 dimensions exhibited average variance extracted (AVE) values above 0.5: physical activity (0.71), sedentary behavior (0.63), consumption of natural products (0.66), sleep quality (0.53), and pollution (0.51). The latent constructs of these dimensions explained more than 50% of the indicator variance (38).

In each dimension, items with relatively poor loadings (Supplementary Table S4.4) and/or items with high residual variance were inspected to identify potential sources of poor model fit (Supplementary Table S4.5).

### 3.6 Internal consistency

The internal consistency values for each domain are shown in Table 6. Cronbach's alpha values ranged between 0.02 and 0.79. The self-care behavior, socioeconomic restrictions, and diet dimensions showed acceptable Cronbach's alpha values of 0.79, 0.78, and 0.71, respectively. In total, five dimensions showed unacceptable Cronbach's alpha values below 0.6 (comorbidities, sedentary behavior, excessive consumption of medicines, consumption of psychoactive substances, and exposure to stress and violence). In particular, comorbidities and sedentary behavior showed the lowest Cronbach's alpha values (0.08 and 0.02, respectively). An overall Cronbach's alpha of 0.80 was obtained across the items from the following dimensions: diet, physical activity, free-time activities, self-care, consumption of medicines, consumption of natural products, consumption of psychoactive products, socioeconomic restrictions, exposure to stress and violence, sleep quality, work environmental risks, pollution, and sedentary behavior.

**Table 6:** Cronbach's alfa and 95% CI per dimension.

Dimensions^a^	No. of items	Alpha	Lower CI	Upper CI
Diet	33	0.71	0.66	0.76
Phys	5	0.69	0.63	0.74
FreeT	12	0.65	0.59	0.71
Selfc	12	0.79	0.75	0.82
CsMed	3	0.42	0.30	0.52
CsNat	4	0.67	0.60	0.72
CPsych	7	0.40	0.29	0.49
EconR	20	0.78	0.74	0.81
StrsV	7	0.57	0.49	0.64
Sleep	6	0.67	0.61	0.73
WrkEn	6	0.60	0.53	0.67
Pollt	12	0.65	0.59	0.70
Sednt	2	0.02	−0.22	0.22
CCI	14	0.08	−0.07	0.23

^a^Diet, negative nutritional habits; Phys, Physical activity; FreeT, healthy free time activities; Selfc, self care; CsMed, Excessive consumption of medicines; CsNat, consumption of natural products; CPsych, consumption of psychoactive products; EconR, economic restrictions; StrsV, exposure to stress and violence; Sleep, sleep quality; WrkEn, work environment risks; Pollt, pollution; Sednt, sedentary behaviour; CCI, comorbidities.

## 4 Discussion

Environmental and lifestyle factors are associated with clinical outcomes in several emerging and re-emerging infectious diseases, including dengue, COVID-19, and influenza (3, 13, 14, 46). The COVID-19 pandemic raised awareness of the influence of previously identified environmental and behavioral factors on clinical outcomes after SARS-CoV-2 infection (13–16, 18–21, 47). In this context, we developed the CELF-Q to collect data on factors influencing health outcomes in infectious diseases, using SARS-CoV-2 infection as a model. The questionnaire included multi-item dimensions evaluating diet quality (including both healthy and unhealthy eating habits), comorbidities, physical activity levels, sedentary behavior, healthy free-time activities, self-care practices, consumption of medicines, consumption of natural products or supplements, socioeconomic characteristics, work environment risks, exposure to pollutants/contaminants, exposure to stress and violence, consumption of psychoactive substances, and sleep quality among the enrolled participants. COVID-19 outcomes and sociodemographic data were also collected.

The CELF-Q was meticulously revised to make it culturally relevant and improve comprehensibility for Cuban individuals. After an extensive review process, the questionnaire was evaluated by a panel of 15 professionals with diverse backgrounds in the health field. Content validity was deemed acceptable across several dimensions based on the results of the content validity ratio (CVR), content validity indices (I-CVI and S-CVI), and Aiken's *V*. Only a few items were identified for revision using this quantitative method. In addition, the experts provided qualitative comments and suggestions for the modification of the items included in the questionnaire.

A test–retest analysis was performed to evaluate the reproducibility of the questionnaire (48). This analysis showed an overall mean kappa coefficient of 0.89 (SD: 0.21) and a mean overall correlation coefficient of 0.99 (0.02), indicating high to almost perfect agreement. To improve reliability, we recommended rephrasing items that exhibited very low agreement between the two applications. Specifically, these included the “take vehicle with driver under alcohol effect” item (self-care behavior), the “consumption of medicines” item (consumption of medicines), and the “acute medical conditions in the last month” item (general).

The CFA results indicated that the unidimensional models for the consumption of natural products and sleep quality constructs provided a good fit to the data. Other models for dimensions such as physical activity, consumption of psychoactive substances, pollution, self-care behavior, exposure to stress and violence, and work environment risks may be improved by carefully revising the variables presenting high residual variance or inspecting modification indices as a guide. Nevertheless, more complex models with multiple factors and their correlations were not evaluated, given the relatively small sample size of the present study. In the context of CFA, a ratio of 5 to 10 subjects per item, up to a total of 300 subjects, has been previously suggested (49). A more recent approach bases the calculation of the sample size on the *N*:*q* ratio, where *N* represents the number of subjects and *q* the number of parameters in the model, recommending a ratio of 20:1 (50). Additional studies with larger samples may be needed for a more thorough evaluation of the constructs included in the CELF-Q questionnaire.

The internal consistency of the CELF-Q was also assessed. The majority of the dimensions had acceptable Cronbach's alpha values, in agreement with the classification proposed by DeVellis and Thorpe (43). In particular, the self-care behavior, socioeconomic restrictions, and diet and nutritional habits dimensions achieved acceptable Cronbach's alpha values (between 0.7 and 0.8). A total of five dimensions were regarded as minimally acceptable (physical activity, free-time activities, consumption of natural products, sleep quality, and exposure to pollutants), and one was considered acceptable but with undesirable consistency (work environment risks) (44, 51). The dimensions with Cronbach's alpha values below .60 (classified as unacceptable) were revised. Notably, comorbidities and sedentary behavior, which included items measuring discordant aspects, exhibited the lowest values (0.08 and 0.02, respectively). In future applications of the CELF-Q, these dimensions will be considered for the construction of indexes rather than scales, following Streiner's suggestion that an index, regarded as the sum of unrelated items, does not have to demonstrate internal consistency (52, 53). Overall, the Cronbach's alpha value obtained (0.80) did not achieve the value reported for the original questionnaire (0.87), but both values can be classified as very good (23, 44).

The CELF-Q has some limitations that need to be acknowledged. First, as with any questionnaire-based instrument, there is potential for recall bias. Some exposure factors may be hard to identify and remember, many disease conditions may remain undiagnosed, and participants may not recall milder forms of existing comorbidities. To overcome this, we included in the CELF-Q a list of common exposure factors and comorbid conditions relevant to the Cuban population.

The list of factors included in the questionnaire was limited to avoid an excessively long instrument, which may lead to an unintentional loss of precision and potentially introduce information and attrition bias. Nevertheless, participants had the opportunity to add other factors and diseases not mentioned in the questionnaire. In addition, to mitigate this limitation, the CELF-Q was administered with the assistance of a member of the research group. Based on the initial testing, future versions may consider including a more comprehensive list based on prevalence or reporting frequency.

The aforementioned constraint related to sample size should be acknowledged. However, fitting separate models for each construct allows the identification of potential causes of misfit and model convergence problems, thereby facilitating the development of models with multiple constructs and dimensions. In addition, the identification of items with poor factor loadings can lead to the creation of more parsimonious models and questionnaires.

Finally, the study population was composed of residents from the cities of Havana and Pinar del Rio, Cuba, which may limit the generalizability of the findings to rural populations or the rest of the country. Nevertheless, the research team does not consider that this limitation has a negative impact on the validation of the questionnaire. However, future studies using the CELF-Q in other populations and in other infectious diseases with pandemic potential (e.g., arbovirosis, influenza, and pneumonia), which have an impact on tropical populations, are strongly encouraged.

Despite these limitations, we believe that the CELF-Q, being a reliable and valid measure of individual risk exposure, has utility as a tool for identifying and quantifying environmental and behavioral factors influencing COVID-19 outcomes and other infectious diseases. This is the first questionnaire to comprehensively evaluate factors influencing infectious disease outcomes in the Cuban population. A potential application of this questionnaire is the development of a risk score or index (for severe outcomes of infectious diseases) based on individual factors and multi-item scales.

Moreover, since biological, socioeconomic, behavioral, and environmental factors could have a positive or negative effect on the risk of SARS-CoV-2 infection and the risk of a severe course of COVID-19, the CELF-Q may potentially contribute to enhance the understanding of the relationship between lifestyle factors and susceptibility to COVID-19 in the Cuban population. Appropriate lifestyle changes toward healthier behaviors could shift the population distribution of infection risk and help prevent severe COVID-19. Promoting a healthy lifestyle could be an effective strategy for controlling the pandemic and mitigating the impact of infectious diseases (3, 14, 54).

## 5 Conclusion

The CELF-Q was developed through an iterative process of desk review and cultural adaptation. The pre-test analysis helped ensure content and face validity, with Cronbach's alpha and Pearson correlation indicating that the questionnaire was internally consistent and reliable in this setting. While some categories proved to be more reliable than others, overall reliability and reproducibility were substantial. The CELF-Q's utility in identifying risk factors for unfavorable COVID-19 clinical outcomes offers a valuable opportunity for the Cuban health system to design and implement early intervention strategies to mitigate life-threatening complications of SARS-CoV-2 and other pathogens.

## Data Availability

The datasets presented in this article are not readily available because restrictions may apply due to reasons of sensitivity. Data are, however, available from the authors upon reasonable request and with permission from the Institute of Tropical Medicine (IPK), Havana, Cuba. Requests to access the datasets should be directed to Waldemar Baldoquín-Rodríguez, wbaldoquin@infomed.sld.cu.

## References

[1] Global Burden of Disease Collaborative Network. Global Burden of Disease Study 2023 (GBD 2023) Risk Exposure Estimates 1990-2023. Seattle, WA: Institute for Health Metrics and Evaluation (IHME) (2025).

[2] Wood S Harrison SE Judd N Bellis MA Hughes K Jones A. The impact of behavioural risk factors on communicable diseases: a systematic review of reviews. BMC Public Health. (2021) 21:2110. doi: 10.1186/s12889-021-12148-y 34789209 PMC8596356

[3] Flegr J Flegr P Príplatová L. The effects of 105 biological, socioeconomic, behavioral, and environmental factors on the risk of SARS-CoV-2 infection and a severe course of COVID-19: a prospective, explorative cohort study. Biol Methods Protocol. (2022) 7:bpac030. doi: 10.1093/biomethods/bpac030 36530561 PMC9750789

[4] Hamer M O'Donovan G Stamatakis E. Lifestyle risk factors, obesity and infectious disease mortality in the general population: linkage study of 97,844 adults from England and Scotland. Prevent Med. (2019) 123:65–70. doi: 10.1016/j.ypmed.2019.03.002 30844499

[5] Zhang JJ Dong X Liu GH Gao YD. Risk and protective factors for COVID-19 morbidity, severity, and mortality. Clin Rev Allergy Immunol. (2023) 64:90–107. doi: 10.1007/s12016-022-08921-5 35044620 PMC8767775

[6] Ho SM Johnson A Tarapore P Janakiram V Zhang X Leung YK. Environmental epigenetics and its implication on disease risk and health outcomes. ILAR J. (2012) 53:289–305. doi: 10.1093/ilar.53.3-4.289 23744968 PMC4021822

[7] Farhud DD. Impact of lifestyle on health. Iran J Public Health. (2015) 44:1442–4. 26744700 PMC4703222

[8] School HM. How to Boost Your Immune System. Boston, MA: Harvard Health Publishing (2021).

[9] Burroughs Peña MS Patel D Rodríguez Leyva D Khan BV Sperling L. Lifestyle risk factors and cardiovascular disease in Cubans and Cuban Americans. Cardiol Res Pract. (2012) 2012:470705. doi: 10.1155/2012/470705 22203917 PMC3235660

[10] Porrata C. Cubans' deadly diet: a wakeup call. MEDICC Rev. (2008) 10:52. doi: 10.37757/MR2008.V10.N2.11 21483370

[11] Thomson B Rojas NA Lacey B Burrett JA Varona-Pérez P Martínez MC . Association of childhood smoking and adult mortality: prospective study of 120 000 Cuban adults. Lancet Glob Health. (2020) 8:e850–7. doi: 10.1016/S2214-109X(20)30221-7 32446350 PMC7248573

[12] Ordunez P Campbell NR. Smoking tobacco, the major cause of death and disability in Cuba. Lancet Glob Health. (2020) 8:e752–3. doi: 10.1016/S2214-109X(20)30226-6 32446341

[13] Wang J Sato T Sakuraba A. Worldwide association of lifestyle-related factors and COVID-19 mortality. Ann Med. (2021) 53:1528–33. doi: 10.1101/2021.08.14.21257136 34435518 PMC8405104

[14] Ko Y Ngai ZN Koh RY Chye SM. Association among lifestyle and risk factors with SARS-CoV-2 infection. Tuberc Respir Dis. (2023) 86:102–10. doi: 10.4046/trd.2022.0125 36597582 PMC10073606

[15] Kapusta J Chudzik M Kałuzińska-Kołat Ż Kołat D Burzyńska M Jankowski P . Do selected lifestyle parameters affect the severity and symptoms of COVID-19 among elderly patients? The retrospective evaluation of individuals from the STOP-COVID registry of the PoLoCOV study. J Infect Public Health. (2023) 16:143–53. doi: 10.1016/j.jiph.2022.12.008 36521330 PMC9743693

[16] Mohsin FM Nahrin R Tonmon TT Nesa M Tithy SA Saha S . Lifestyle and Comorbidity-Related Risk Factors of Severe and Critical COVID-19 Infection: A Comparative Study Among Survived COVID-19 Patients in Bangladesh. Infect Drug Resist. (2021) 14:4057–66. doi: 10.2147/IDR.S331470 34616163 PMC8489920

[17] Tessier AJ Moyen A Lawson C Rappaport AI Yousif H Fleurent-Grégoire C . Lifestyle behavior changes and associated risk factors during the COVID-19 pandemic: results from the canadian COVIDiet online cohort study. JMIR Public Health Surveill. (2023) 9:e43786. doi: 10.2196/43786 36848226 PMC10131911

[18] Hamer M Kivimäki M Gale CR Batty GD. Lifestyle risk factors, inflammatory mechanisms, and COVID-19 hospitalization: a community-based cohort study of 387,109 adults in UK. Brain Behav Immun. (2020) 87:184–7. doi: 10.1016/j.bbi.2020.05.059 32454138 PMC7245300

[19] Tavakol Z Ghannadi S Tabesh MR Halabchi F Noormohammadpour P Akbarpour S . Relationship between physical activity, healthy lifestyle and COVID-19 disease severity; a cross-sectional study. Z Gesundh Wiss. (2023) 31:267–75. doi: 10.1007/s10389-020-01468-9 33558839 PMC7858040

[20] Gao C Zhao Z Li F Liu JL Xu H Zeng Y . The impact of individual lifestyle and status on the acquisition of COVID-19: a case-Control study. PLoS ONE. (2020) 15:e0241540. doi: 10.1371/journal.pone.0241540 33152004 PMC7643946

[21] Aouissi HA Kechebar MSA Ababsa M Roufayel R Neji B Petrisor AI . The importance of behavioral and native factors on COVID-19 infection and severity: insights from a preliminary cross-sectional study. Healthcare. (2022) 10:1341. doi: 10.3390/healthcare10071341 35885867 PMC9323463

[22] Arrivillaga M Salazar IC Correa D. Creencias sobre la salud y su relación con las prácticas de riesgo o de protección en jóvenes universitarios. Colomb Méd. (2003) 34:186–95. doi: 10.25100/cm.v34i.4.273

[23] Arrivillaga M Salazar IC. Creencias relacionadas con el estilo de vida de jóvenes latinoamericanos. Behav Psychol. (2005) 13:19–36.

[24] C. D. Autores. Protocolo de actuación nacional para el enfrentamiento a la COVID 19, Versión 1.4. La Habana: MINSAP (2020).

[25] Zamanzadeh V Ghahramanian A Rassouli M Abbaszadeh A Alavi-Majd H Nikanfar AR. Design and implementation content validity study: development of an instrument for measuring patient-centered communication. J. Caring Sci. (2015) 4:165. doi: 10.15171/jcs.2015.017 26161370 PMC4484991

[26] Rubio DM Berg-Weger M Tebb SS Lee ES Rauch S. Objectifying content validity: conducting a content validity study in social work research. Soc Work Res. (2003) 27:94–104. doi: 10.1093/swr/27.2.94

[27] Almanasreh E Moles R Chen TF. Evaluation of methods used for estimating content validity. Res. Soc. Adm. Pharm. (2019) 15:214–21. doi: 10.1016/j.sapharm.2018.03.066 29606610

[28] Aiken LR. Three coefficients for analyzing the reliability and validity of ratings. Educ Psychol Meas. (1985) 45:131–42. doi: 10.1177/0013164485451012

[29] Lawshe CH. A quantitative approach to content validity. Pers. Psychol. (1975) 28:563–75. doi: 10.1111/j.1744-6570.1975.tb01393.x

[30] Penfield RD Giacobbi PR Jr. Applying a score confidence interval to Aiken's item content-relevance index. Meas Phys Educ Exerc Sci. (2004) 8:213–25. doi: 10.1207/s15327841mpee0804_3

[31] Gilbert GE Prion S. Making sense of methods and measurement: Lawshe's content validity index. Clin Simul Nurs. (2016) 12:530–1. doi: 10.1016/j.ecns.2016.08.002

[32] Davis LL. Instrument review: getting the most from a panel of experts. Appl Nurs Res. (1992) 5:194–7. doi: 10.1016/S0897-1897(05)80008-4

[33] Bartlett MS. The effect of standardization on a χ2 approximation in factor analysis. Biometrika. (1951) 38:337–44. doi: 10.1093/biomet/38.3-4.337

[34] Kaiser HF Rice J. Little jiffy, mark iv. Educ Psychol Meas. (1974) 34:111–7. doi: 10.1177/001316447403400115

[35] Li CH. Confirmatory factor analysis with ordinal data: comparing robust maximum likelihood and diagonally weighted least squares. Behav Res. (2016) 48:936–49. doi: 10.3758/s13428-015-0619-7 26174714

[36] Rosseel Y. Lavaan: an r package for structural equation modeling. J Stat Softw. (2012) 48:1–36. doi: 10.18637/jss.v048.i02

[37] Rosseel Y Jorgensen Terrence D., De Wilde Luc. Lavaan: Latent Variable Analysis. R package version 0.6-20. Vienna: R Foundation for Statistical Computing (2025).

[38] Fornell C Larcker DF. Evaluating structural equation models with unobservable variables and measurement error. J Mark Res. (1981) 18:39–50. doi: 10.1177/002224378101800104

[39] Tucker LR Lewis C. A reliability coefficient for maximum likelihood factor analysis. Psychometrika. (1973) 38:1–10. doi: 10.1007/BF02291170

[40] Hu L Bentler PM. Cutoff criteria for fit indexes in covariance structure analysis: Conventional criteria versus new alternatives. Struct Equ Modeling. (1999) 6:1–55. doi: 10.1080/10705519909540118

[41] Brown TA. Confirmatory Factor Analysis for Applied Research. New York, NY: Guilford Publications (2015).

[42] Peterson PL Baker EL McGaw B. International Encyclopedia of Education, 3rd Edn. Oxford: Academic Press (2010).

[43] DeVellis RF Thorpe CT. Scale Development: Theory and Applications, 5th Edn. Thousand Oaks, CA: Sage publications, Inc. (2022).

[44] DeVellis RF Thorpe CT. Scale Development: Theory and Applications. Thousand Oaks, CA: Sage Publications (2021).

[45] R Core Team (2024). R: a language and environment for statistical computing. Vienna: R Foundation for Statistical Computing. Available online at: https://www.R-project.org/

[46] Tassakos A Kloppman A Louie JCY. The impact of diet quality on COVID-19 severity and outcomes-a scoping review. Curr Nutr Rep. (2025) 14:27. doi: 10.1007/s13668-025-00618-3 39891806 PMC11787171

[47] Li S Hua X. Modifiable lifestyle factors and severe COVID-19 risk: a Mendelian randomisation study. BMC Med Genomics. (2021) 14:38. doi: 10.1186/s12920-021-00887-1 33536004 PMC7856619

[48] Ranganathan P Caduff C Frampton CMA. Designing and validating a research questionnaire - Part 2. Perspect Clin Res. (2024) 15:42–5. doi: 10.4103/picr.picr_318_23 38282630 PMC10810057

[49] Tinsley HEA Tinsley DJ. Uses of factor analysis in counseling psychology research. J Couns Psychol. (1987) 34:414–24. doi: 10.1037/0022-0167.34.4.414

[50] Kline RB. Principles and Practice of Structural Equation Modeling, 4th Edn. New York, NY: The Guilford Press (2016).

[51] Robertson O Scott Evans M%JPQ. Just how reliable is your internal reliability? An overview of Cronbach's alpha (α). PsyPag Q. (2020) 1:23–7. doi: 10.53841/bpspag.2020.1.115.23

[52] Streiner DL. Being inconsistent about consistency: when coefficient alpha does and doesn't matter. J Pers Assess. (2003) 80:217–22. doi: 10.1207/S15327752JPA8003_01 12763696

[53] Stefana A Damiani S Granziol U Provenzani U Solmi M Youngstrom EA . Psychological, psychiatric, and behavioral sciences measurement scales: best practice guidelines for their development and validation. Front Psychol. (2025) 15:1494261. doi: 10.3389/fpsyg.2024.1494261 39916786 PMC11798685

[54] Wang Y Su B Alcalde-Herraiz M Barclay NL Tian Y Li C . Modifiable lifestyle factors and the risk of post-COVID-19 multisystem sequelae, hospitalization, and death. Nat Commun. (2024) 15:6363. doi: 10.1038/s41467-024-50495-7 39075060 PMC11286928

