# Supplementary material for: Development and validation of a questionnaire to assess environmental and lifestyle factors influencing infectious disease outcomes: application to SARS-CoV-2-infected individuals in Cuba
Source: Front Public Health. 2026 Jun 29;14:1805212. doi: 10.3389/fpubh.2026.1805212 (PMC13357406; doi:10.3389/fpubh.2026.1805212)
Supplement: Supplementary file 1 [file Table_1.docx]

Supplementary Material

# Questionnaire to assess Cuban population´s environmental/lifestyle’s factors - Spanish version

**CUESTIONARIO SOBRE HÁBITOS, ESTILOS DE VIDA E INFLUENCIA MEDIOAMBIENTE**

**Section 1. Socio- demographic data.**

1. **Sexo (seleccione uno)**

- Mujer (1);
- Hombre (2);

1. **Color de la piel (seleccione uno)**

- Blanco (1);
- Mestizo (2);
- Negro (3);

1. **Edad**

|_|_|_| años cumplidos

1. **Peso estimado:**

|_|_|_| Kg

1. **Estatura estimada:**

|_|.|_|_| m

1. **¿Estado civil? (seleccione 1)**

- Soltero/a (1);
- Casado/a (2);
- Viudo/a (3);
- Divorciado/a (4);
- Unión consensuada (5);

1. **¿Tiene hijos?**

- Si (1); ⇒ Continue with question 7 a.
- No (2);
- 7 a. ¿Cuántos? |_|_|

1. **Ocupación (ahora o en caso de retirado: su ocupación cuando estaba trabajando):**

(seleccione 1)

- Desocupado (1);
- Obrero (2);
- Campesino (3);
- Ama de casa (4);
- Estudiante (5);
- Militar (6);
- Trabajador por cuenta propia (7);
- Profesional (8); ⇒ Please, complete question 8 a.
- Otro (9); ⇒ Please, Respond question 8 b.
- If Ocupación = Profesional (8) was selected, please respond question 8 a.
- 8 a. Tipo de trabajo: _______________________________
- If Ocupación = Otro (9) was selected, please respond question 8 b.
- 8 b. ¿Cuál? ______________________________________

1. **¿Ciudad o pueblo en la que permanece la mayor parte del tiempo?**

______________

1. **¿Usted también vivía en este lugar durante el año 2018? (seleccione 1)**

- Si (1);
- No (2);

1. **¿Ocurrió algún cambio en el área adonde vives en los últimos dos años (nuevas construcciones, nuevas fábricas, movimiento de personas (si aumentó o disminuyó), tráfico de vehículos (si aumentó o disminuyó), otros….)?**

- Si (1); ⇒ continue with question 11 a.
- No (2);
- 11 a. ¿Cuál? _____________________________
- 11 b. nuevas construcciones ☐ Si (1); ☐ No (2);
- 11 c. nuevas fábricas ☐ Si (1); ☐ No (2);
- 11 d. movimiento de personas ☐ Si (1); ☐ No (2);
- 11 e. aumento del tráfico vehicular ☐ Si (1); ☐ No (2);

**Section 2. Health related.**

1. **En general Usted diría que su salud es:**

**(seleccione 1)**

- Buena (1); ☐ Regular (2); ☐ Mala (3);

1. **¿Ha padecido alguna enfermedad, en el último mes? (seleccione 1)**

- Si (1); ⇒ continue with question 13 a.
- No (2);
- 13 a. ¿Cuál? ____________________

1. **¿Padece alguna enfermedad crónica?**

- Si (1); ⇒ continue with questions 14 a- 14g, below.
- No (2);
- ¿Cuál?
- 14 a. Diabetes ☐ Si (1); ☐ No (2);
- 14 b. Asma ☐ Si (1); ☐ No (2);
- 14 c. Hipertensión ☐ Si (1); ☐ No (2);
- 14 d. Colesterol /triglicéridos altos ☐ Si (1); ☐ No (2);
- 14 e. Alergia ☐ Si (1); ☐ No (2);
- 14 f. Cáncer ☐ Si (1); ☐ No (2);
- 14 g. Otro: > ___________________________________

1. **¿Toma algún medicamento en este momento?**

- Si (1); ⇒ Continue with questions 15 a.
- No (2);
- 15 a. ¿Cuál? ___________________
- 15 b. ¿En qué dosis? ___________________

1. **¿Usted ha tomado muchos medicamentos, en su vida?**

- Si (1); ⇒ Continue with questions 16 a – c.
- No (2);

Diga los 3 que más ha tomado:

Medicamento ____ Período de consumo ____ Dosis

16 a1 _______________ 16 a2 ______________ 16 a3 _____________

16 b1 _______________ 16 b2 ______________ 16 b3 _____________

16 c1 _______________ 16 c2 ______________ 16 c3 _____________

1. **¿Usted se automedica cuando se siente mal? (por ejemplo un dolor de cabeza, catarro, gripe o problema digestivo)**

- Si (1); ⇒ Continue with question 17 a.
- No (2);
- 17 a. ¿Qué medicamento toma? ________________________
- 17 b. ¿Dosis? ______________

1. **¿Usted utiliza la medicina natural (te, cocimientos de plantas, mieles u otros)cuando se siente mal? (por ejemplo un dolor de cabeza, catarro, gripe o problema digestivo)**

- Sí (1); ⇒ Continue with question 18 a.
- No (2);
- 18 a. ¿Cuál? > _________________________________

1. **¿Usted consume algún suplemento?**

- Sí (1); ⇒ continue with questions 19 a-d.
- No (2);
- ¿Cuál?
- 19 a. vitamínico ☐ Si (1); ☐ No (2);
- 19 b. nutricional ☐ Si (1); ☐ No (2);
- 19 c. antioxidante ☐ Si (1); ☐ No (2);
- 19 d. otro: _____________

1. **¿Ha sufrido alguna operación quirúrgica en su vida?**

- Sí (1); ⇒ Continue with question 20 a.
- No (2);
- 20 a. ¿Cuál? ____________________________

1. ¿Ha padecido usted enfermedades respiratorias a repetición?

- Sí (1); ⇒ Continue with question 21 a.
- No (2);
- 21 a. ¿En qué año se enfermó? |_|_|_|_|
- 21 b. ¿Estuvo enfermo durante más de 10 días? ☐ Si (1); ☐ No (2);
- 21 c. ¿Estuvo hospitalizado? ☐ Si (1); ☐ No (2);
- 21 d. ¿Se ha quedado con algún síntoma o padecimiento durante más de 3 meses?
- Sí (1) ; ⇒ continue to question 21 e.
- No (2);
- 21 e. ¿Cuál? ____________________

1. **¿Alguien de su familia (Su padres o hermanos) padecen alguna enfermedad crónica?**

- Sí (1); ⇒ continue with questions 22 a- 22g, below.
- No (2);
- ¿Cuál?
- 22 a. Diabetes ☐ Si (1); ☐ No (2);
- 22 b. Asma ☐ Si (1); ☐ No (2);
- 22 c. Hipertensión ☐ Si (1); ☐ No (2);
- 22 d. Colesterol /triglicéridos altos: ☐ Si (1); ☐ No (2);
- 22 e. alergia ☐ Si (1); ☐ No (2);
- 22 f. Cáncer ☐ Si (1); ☐ No (2);
- 22 g. otro: > ________________________________

1. **¿Alguien de su familia ha padecido COVID- 19?**

- Si (1); ☐ No (2);

1. **Alguien de su familia ha estado grave por COVID-19?**

- Si (1); ☐ No (2);

1. **¿Dónde vivía su madre antes de tenerlo a usted?**

- en el campo (1); ☐ en la ciudad (2);

1. **¿Dónde vivía su padre antes de tenerlo a usted?**

- en el campo (1) ; ☐ en la ciudad (2);

1. **¿Cuál era la edad de su madre cuando lo tuvo?**

- <20 años (1); ☐ entre 20 y 30 años(2); ☐ entre 30 y 40 años(3); ☐ >40 años (4);

**Section 3. Physical activity**

1. **¿Hace ejercicio, como por ejemplo caminar, trotar, correr, andar** **en bicicleta, aeróbicos, va a gimnasio, otros?**

- Sí (1); ⇒ Continue to question 28 a.
- No (2);
- 28 a. ¿Con qué frecuencia?
- Todos los días (1); ☐ Al menos cinco días por semana (2); ☐ Tres veces por semana (3); ☐ Una o dos veces por semana (4);

1. **¿Realiza ejercicios para mejorar su estado físico (por ejemplo funcionamiento cardiaco)?**

- Sí (1); ⇒ Continue to question 29 a.
- No (2);
- 29 a ¿Con qué frecuencia?
- Todos los días (1); ☐ Al menos cinco días por semana (2);
- Tres veces por semana (3); ☐ Una o dos veces por semana (4);

1. **¿Practica ejercicios que le ayuden a relajarse y/o equilibrarse (por ejemplo taichi, kun fu, yoga, danza, meditación, relajación)?**

- Sí (1); ⇒ Continue to question 30 a.
- No (2);
- 30 a ¿Con qué frecuencia?
- Todos los días (1); ☐ Al menos cinco días por semana (2);
- Tres veces por semana (3); ☐ Una o dos veces por semana (4);

1. **¿Participa en programas o actividades de ejercicio físico bajo supervisión de alguno de los siguientes especialistas?**

- 31 a. un entrenador
- Si (1); ☐ No (2);
- 31 b. un fisioterapeuta
- Si (1); ☐ No (2);

**Section 4. Free time activities**

1. **¿De cuánto tiempo libre (para descansar o disfrutar de actividades que le gustan) usted dispone en la semana (en horas, como promedio)?**

- 48 horas (1);
- 24 horas (2);
- 12 horas (3);
- entre 4 y 6 horas (4);
- Menos de 4 horas (5);

1. **¿De cuánto tiempo libre (para descansar o disfrutar de actividades que le gustan) usted dispone cada día (en horas, como promedio)?**

- 8 horas (1);
- 6 horas (2);
- 4 horas (3);
- Menos de 4 horas/ día en promedio (4);

1. **¿Qué prefiere hacer en su tiempo libre?**

- 34 a. ir al cine ☐ Si (1); ☐ No (2);
- 34 b. oír música ☐ Si (1); ☐ No (2);
- 34 c. pasear ☐ Si (1); ☐ No (2);
- 34 d. ver TV ☐ Si (1); ☐ No (2);
- 34 e. estar con amigos/familia ☐ Si (1); ☐ No (2);
- 34 f. estudiar ☐ Si (1); ☐ No (2);
- 34 g. hacer deporte ☐ Si (1); ☐ No (2);
- 34 h. leer ☐ Si (1); ☐ No (2);
- 34 i. hacer nada ☐ Si (1); ☐ No (2);
- 34 j. jugar con mis nietos/hijos ☐ Si (1); ☐ No (2);
- 34 k. otro: __________________

**Section 5. Selfcare**

1. **¿Consulta al dentista de forma preventiva por lo menos una vez al año?**

- Sí (1); ☐ No (2);

1. **¿Consulta al médico de forma preventiva por lo menos una vez al año?**

- Sí (1); ☐ No (2);

1. **¿Evita las exposiciones prolongadas al sol?**

- Sí (1); ☐ No (2);

1. **¿Usa protectores solares cuando se expone al sol?**

- Sí (1); ⇒ continue with questions 38 a – 38 f.
- No (2);
- ¿Cuál tipo de protección?
- 38 a. Cremas ☐ Sí (1); ☐ No (2);
- 38 b. ropas ☐ Sí (1); ☐ No (2);
- 38 c. gorras ☐ Sí (1); ☐ No (2);
- 38 d. sombreros ☐ Sí (1); ☐ No (2);
- 38 e. gafas ☐ Sí (1); ☐ No (2);
- 38 f. otros: _______________

1. **¿Chequea al menos una vez al año su presión arterial?**

- Sí (1); ☐ No (2);

1. **¿Se realiza exámenes de colesterol o triglicéridos por lo menos una vez al año?**

- Sí (1); ☐ No (2);

1. **¿Se realiza exámenes de glicemia por lo menos una vez al año?**

- Sí (1); ☐ No (2);

1. **¿Observa su cuerpo con detenimiento para detectar cambios físicos?**

- Sí (1); ☐ No (2);

1. **PARA MUJERES: ¿Se examina los senos en búsqueda de nódulos u otros cambios, al menos una vez al mes?**

- Sí (1); ☐ No (2);

1. **PARA MUJERES mayores de 25 años: ¿Se realiza la prueba citológica 1 vez al año?**

- Sí (1); ☐ No (2);

1. **PARA HOMBRES MAYORES DE 40 AÑOS: ¿Se realiza exámenes de próstata anualmente (antígeno prostático y/o tacto renal)?**

- Sí (1); ☐ No (2);

1. **¿Aborda un vehículo manejado por algún conductor bajo efectos del alcohol u otras sustancias psicoactivas?**

- Sí (1); ☐ No (2);

1. **¿Obedece las leyes de tránsito, sea peatón o conductor?**

- Siempre (1); ☐ la mayoría del tiempo (2);
- pocas veces (3); ☐ nunca (4);

1. **¿Usa cinturón de seguridad cuando va como conductor o pasajero en un auto?**

- Siempre (1); ☐ la mayoría del tiempo (2);
- pocas veces (3); ☐ nunca (4);

**Section 6. Health practices**

1. **¿Cuántas horas diarias trabajas en un día laboral en promedio?**

- < 1 hora/día laboral en promedio (1);
- entre 1 y 3 horas/día laboral en promedio (2);
- entre 4 y 7 horas/día laboral en promedio (3);
- > 7 horas/día laboral en promedio (4);

1. **¿Cuánto tiempo usted está sentado en su trabajo/estudio (en promedio por día)?**

- Nunca (1);
- entre 1 y 2 horas/día (2);
- entre 3 y 5 horas/día (3);
- entre 6 y 8 horas/día (4);
- Siempre (aclare cuentas horas trabaja) (5);

1. **¿Dónde se encuentra su lugar de trabajo/estudio?**

- al aire libre (1);
- Bien ventilado (2);
- poco ventilado (3);
- cerrado con aire acondicionado (4);
- cerrado sin aire acondicionado (5);
- Otro: (6); ⇒ continue to question 51 a.
- 51 a. Otro? Especifique > ________________________________

1. **¿Cómo usted percibe la actividad física en su trabajo?**

- trabajo físico fuerte/intenso (1);
- trabajo físico intermedio (2);
- trabajo físico liviano (3);
- ningún trabajo físico (4);

1. **El ambiente en su trabajo es:**

- amable/cordial (1);
- con algunas discusiones/tensiones (2);
- con muchas discusiones/tensiones (3);
- no es nada cordial ni amable (4);

1. **¿Usted está acostumbrado de tomar un descanso en el día laboral?**

- Si (1); ⇒ continue with question 54 a.
- No (2);

54 a. ¿Cuánto tiempo?

- Menor de 1 hora diaria (1);
- entre 1 y 2 horas diarias (2);
- más de 2 horas diarias (3);

1. **Considera usted que su trabajo le genera:**

- ningún estrés (1);
- poco estrés (2);
- bastante estrés (3);
- mucho estrés (4);
- demasiado estrés (5);

1. **¿Usted disfruta de su trabajo?**

- Siempre (1);
- la mayoría del tiempo (2);
- pocas veces (3);
- nunca (4);

1. **¿Usa algún medio de protección para evitar riesgo en su práctica laboral?**

- Si (1); ☐ No (2);

1. **¿Ha tenido algún accidente laboral?**

- Sí (1); ⇒ continue with question 58 a.
- No (2);
- 58 a. ¿Cuál? _____________________

1. **¿Usted siente que hay mucho estrés en su casa actual?**

- Si (1); ☐ No (2);

1. **¿Tuvo mucho estrés en tu casa cuando usted vivió con sus padres?**

- Si (1); ☐ No (2);

1. **¿Hubo violencia verbal o física por parte de sus padres?**

- Si (1); ☐ No (2);

1. **¿Hay violencia en su entorno (manzana, escuela, trabajo, etc.)?**

- Si (1); ☐ No (2);

1. **¿Usted aplica productos químicos en su casa (como para matar insectos, mosquitos, moscas, cucarachas, etc.)?**

- Si (1); ☐ No (2);

**Section 7. Dietary habits**

1. **¿Qué cantidad de agua Ingiere al día?**

- < 4 vasos (1); ☐ entre 4 y 8 vasos (2); ☐ Mas de 8 vasos (3);

1. **¿Mantiene un horario regular en las comidas?**

- Si (1); ☐ No (2);

1. **¿Desayuna antes de iniciar su actividad diaria?**

- Si (1); ☐ No (2);

1. **¿Realiza dietas y/o métodos que le provoquen una rápida y fácil pérdida de peso?**

- Si (1); ☐ No (2);

1. **¿Consume más de tres veces por semana comidas rápidas (pizza, hamburguesa, perro caliente, frituras)?**

- Si (1); ☐ No (2);

1. **¿Tiene hábitos de ingerir sus alimentos con mucha sal?**

- Si (1); ☐ No (2);

1. **¿Les gustan los alimentos dulces?**

- Si (1); ☐ No (2);

1. **¿Cuál de estas comidas ingiere durante el día?**

- 71 a. desayuno ☐ Si (1); ☐ No (2);
- 71 b. merienda en la mañana ☐ Si (1); ☐ No (2);
- 71 c. almuerzo ☐ Si (1); ☐ No (2);
- 71 d. merienda en la tarde ☐ Si (1); ☐ No (2);
- 71 e. cena ☐ Si (1); ☐ No (2);
- 71 f. merienda después de la cena ☐ Si (1); ☐ No (2);

1. **¿Toma café?**

- Sí (1); ⇒ Continue with question 72 a -b.
- No (2);
- 72 a. ¿cuántas tazas/día?: |_|_|
- 72 b. ¿con azúcar?: ☐ Si (1); ☐ No (2);

1. **¿Consume refrescos enlatados o en polvo más de cuatro veces en la** **semana?**

- Si (1); ☐ No (2);

1. **¿Consume dulces, helados y pasteles más de dos veces en la** **semana?**

- Si (1); ☐ No (2);

1. **¿Utiliza algún condimento para preparar las comidas?**

- Sí (1); ⇒ Continue with questions 75 a-d
- No (2);
- ¿cuál?
- 75 a. sal ☐ Si (1); ☐ No (2);
- 75 b. condimentos deshidratados ☐ Si (1); ☐ No (2);
- 75 c. condimentos naturales ☐ Si (1); ☐ No (2);
- 75 d. cuadraditos, pastillitas, sobres o sazón completo ☐ Si (1); ☐ No (2);

1. **¿Ingiere al menos una vez por semana alguno de estos alimentos?** **indique cuales:**

- 76 a. vegetales ☐ Si (1); ☐ No (2);
- 76 b. frutas ☐ Si (1); ☐ No (2);
- 76 c. panes ☐ Si (1); ☐ No (2);
- 76 d. cereales ☐ Si (1); ☐ No (2);
- 76 e. productos lácteos ☐ Si (1); ☐ No (2);
- 76 f. granos enteros ☐ Si (1); ☐ No (2);
- 76 g. carne ☐ Si (1); ☐ No (2);
- 76 h. pescado ☐ Si (1); ☐ No (2);
- 76 i. huevos ☐ Si (1); ☐ No (2);

1. **¿Ingiere algunos de estos alimentos al menos 1 vez por semana?**

- 77 a. Mantequilla ☐ Si (1); ☐ No (2);
- 77 b. queso crema ☐ Si (1); ☐ No (2);
- 77 c. carnes de cerdo ☐ Si (1); ☐ No (2);
- 77 d. mayonesas ☐ Si (1); ☐ No (2);
- 77 e. salsas en general ☐ Si (1); ☐ No (2);
- 77 f. chicharrones ☐ Si (1); ☐ No (2);

1. **¿Cuántas veces come carne en la semana?**

- 1 vez (1); ☐ 2 veces (2); ☐ Más de 2 veces (3);

1. **¿Qué carnes come habitualmente?**

- 79 a. pescado ☐ Si (1); ☐ No (2);
- 79 b. pollo ☐ Si (1); ☐ No (2);
- 79 c. cerdo ☐ Si (1); ☐ No (2);
- 79 d. res ☐ Si (1); ☐ No (2);
- 79 e. carnero ☐ Si (1); ☐ No (2);

1. **¿Consume productos ahumados más que una vez por semana?**

- Sí (1); ⇒ Continue with questions 80 a-d
- No (2);
- ¿cuál?
- 80 a. Lomo ahumado ☐ Si (1); ☐ No (2);
- 80 b. Salchichas ☐ Si (1); ☐ No (2);
- 80 c. Jamón ☐ Si (1); ☐ No (2);
- 80 d. Chorizos ☐ Si (1); ☐ No (2);

**Section 8. Psycoactive substances.**

1. **¿Fuma cigarrillo o tabaco?**

- Si (1); ⇒ Continue with question 82.
- No (2);
- 81 a. ¿Fuma más de 10 cigarrillos al día? ☐ Si (1); ☐ No (2);
- 81 b. ¿Permite que fumen en su presencia? ☐ Si (1); ☐ No (2);

1. **¿Cuántas veces consume alcohol durante la semana?**

- Nunca (1);
- < 1 vez/semana (2);
- cada día (3);
- solamente el fin de semana (4);
- casi cada día (5);

1. **¿Consume algún psicofármaco (diazepam, clorodiazepoxido, nitrazepan, alprazolam) u otros) cuando se enfrenta a situaciones de angustia o problemas en su vida?**

- Si (1); ☐ No (2);

1. **¿Ha consumido alguna vez sustancias psicoactivas (marihuana, cocaína, basuco, éxtasis, u otras)?**

- Si (1); ☐ No (2);

1. **¿Uno o ambos de sus padres fuman?**

- Si (1); ☐ No (2);

1. **¿Uno o ambos de sus padres toman alcohol cada día?**

- Si (1); ☐ No (2);

**Section 9. Sleep**

1. **¿Cuál es el promedio de horas que duerme diariamente?**

- Menos de 6 horas (1);
- entre 6 y 8 horas (2);
- Más de 8 horas (3);

1. **¿Duerme bien y se levanta descansado habitualmente?**

- Si (1); ☐ No (2);

1. **¿Le cuesta trabajo quedarse dormido habitualmente?**

- Sí (1); No (2);

1. **¿Se despierta en varias ocasiones durante la noche habitualmente?**

- Sí (1); No (2);

1. **¿Se mantiene habitualmente con sueño durante el día?**

- Sí (1); No (2);

1. **¿Utiliza habitualmente pastillas para dormir?**

- Sí (1); No (2);

**Section 10. Socio-economics**

1. **¿Qué fuente de abasto de agua usa habitualmente?**

- 95 a. Acueducto ☐ Si (1); ☐ No (2);
- 95 b. Pozo ☐ Si (1); ☐ No (2);
- 95 c. Rio ☐ Si (1); ☐ No (2);
- 95 d. otro: ☐ Si (1); ☐ No (2);
- 95 e. Cual otro _________________________________

1. **¿Utiliza algún método de desinfección del agua?**

- Si (1); ⇒ Continue with question 94 a.
- No (2);
- **¿Cuál método utiliza para la desinfección del agua?**
- 94 a. Ebullición (hervir) ☐ Si (1); ☐ No (2);
- 94 b. Hipoclorito ☐ Si (1); ☐ No (2);
- 94 c. Otros
- Sí (1); ⇒ continue with question 94 d.
- No (2);
- 94 d. Cual otro? _______________________

1. **¿Con qué frecuencia reciben el agua habitualmente?**

- Diario (1); ☐ En días alternos (2); ☐ Dos veces por semana (3);
- Una vez por semana (4); ☐ Menos de una vez por semana (5);

1. **¿Qué cantidad de personas conviven en su vivienda? (Incluyéndolo a Ud.)** |_|_|
2. **¿Cuántas habitaciones (en total, incluyendo sala, cocina, baño, etc.) posee la vivienda?** |_|_|.

- 97 a. **¿Cuántas habitaciones se dedican exclusivamente a dormitorio?** (excluyendo cocina, baño, pasillos, etc.) |_|_|.

1. **De las personas que viven en la vivienda, puede decirnos cuántas**

- **Sexo:**
- 98 a1. |_|_| Masculino 98 a2. |_|_| Femenino
- **Edad:**
- 98 b1. |_|_| < 5 años 98 b2. |_|_| 5 -14 años 98 b3. |_|_| 15 – 24 años
- 98 b4. |_|_|25-44 años 98 b5. |_|_| 45 – 64 años 98 b6. |_|_| 65 y más años
- **Escolaridad:**
- 98 c1. |_|_| Primaria 98 c2. |_|_| Secundaria 98 c3. |_|_| Pre-Universitario/Técnico 98 c4. |_|_| Universidad
- **Ocupación**:
- 98 d1. |_|_| Obrero 98 d2. |_|_| Campesino 98 d3. |_|_| Ama de casa
- 98 d4. |_|_| Estudiante 98 d5 |_|_| Militar 98 d6. |_|_| Profesional
- 98 d7. |_|_| Desocupado 98 d8. |_|_| Trabajador por cuenta propia
- 98 d9. |_|_| Otro: 98 d10. ¿cuál otro?____________

1. **¿El promedio de gastos aproximado de su familia en un mes (en el último año) puede ser considerado en alguno de estos rangos?**

| **99 a. Moneda nacional** | **99 b. Moneda convertible** |
| --- | --- |
| ☐ menos de 200 pesos (1) | ☐ menos de 10 CUC (1); |
| ☐ 201-400 pesos (2) | ☐ 11-50 CUC (2); |
| ☐ 401-600 pesos (3) | ☐ 51-100 CUC (3); |
| ☐ 601-800 pesos (4) | ☐ 101-300 CUC (4); |
| ☐ 801-1000 pesos (5) | ☐ 301-500 CUC (5); |
| ☐ más 1001 pesos (6) | ☐ más de 501 CUC (6); |
| ☐ No sabe (7) | ☐ no sabe (7); |
| ☐ No responde (8) | ☐ no responde (8); |

1. **¿De los siguientes equipos electrodomésticos y medios de transporte, puede decirnos cuales tiene Ud. en su vivienda (funcionando adecuadamente)?**

| **item** | **Marque sí o no** |
| --- | --- |
| 100 a. Automóvil o moto | ☐ Si (1); ☐ No (2); |
| 100 b. Bicicleta | ☐ Si (1); ☐ No (2); |
| 100 c. Aire acondicionado | ☐ Si (1); ☐ No (2); |
| 100 d. Refrigerador | ☐ Si (1); ☐ No (2); |
| 100 e. Horno microwave | ☐ Si (1); ☐ No (2); |
| 100 f. Lavadora | ☐ Si (1); ☐ No (2); |
| 100 g. Computadora | ☐ Si (1); ☐ No (2); |
| 100 h. Ventilador | ☐ Si (1); ☐ No (2); |
| 100 i. Televisor Pantalla plana | ☐ Si (1); ☐ No (2); |
| 100 j. Batidora | ☐ Si (1); ☐ No (2); |
| 100 k. Televisor | ☐ Si (1); ☐ No (2); |
| 100 l. Cocina Gas | ☐ Si (1); ☐ No (2); |

**Section 11. Pollution**

1. **¿Hay contaminantes ambientales en la manzana (desechos domésticos sólidos, desechos constructivos, mucho tráfico de vehículos, químicos o tóxicos de fábrica o industria)?**

- Si (1); ⇒ continue with questions 108 a-b.
- No (2);
- 101 a. ¿Cuál?: ______
- 101 b. Cantidad:
- muchos contaminantes (1); ☐ pocos contaminantes (2);
- 101 c. desechos domésticos sólidos ☐ Si (1); ☐ No (2);
- 101 d. desechos constructivos, ☐ Si (1); ☐ No (2);
- 101 e. mucho tráfico de vehículos, ☐ Si (1); ☐ No (2);
- 101 f. químicos o tóxicos de fábrica o industria ☐ Si (1); ☐ No (2);

1. **¿Hay contaminantes ambientales en el entorno del centro de trabajo (desechos domésticos sólidos, desechos constructivos, mucho tráfico de vehículos, químicos o tóxicos de fábrica o industria)?**

- Si (1); ⇒ continue with questions 108 a-b.
- No (2);
- 102 a. ¿Cuál?: ______
- 102 b. Cantidad:
- muchos contaminantes (1); ☐ pocos contaminantes (2);
- 102 c. desechos domésticos sólidos ☐ Si (1); ☐ No (2);
- 102 d. desechos constructivos, ☐ Si (1); ☐ No (2);
- 102 e. mucho tráfico de vehículos, ☐ Si (1); ☐ No (2);
- 102 f. químicos o tóxicos de fábrica o industria ☐ Si (1); ☐ No (2);

**Section 12. Covid-19 disease and symptoms**

1. **Síntomas relacionados con COVID-19**

- 103 a. Fiebre ☐ Si (1); ☐ No (2);
- 103 b. Tos ☐ Si (1); ☐ No (2);
- 103 c. Disnea ☐ Si (1); ☐ No (2);
- 103 d. Odinofagia ☐ Si (1); ☐ No (2);
- 103 e. Perdida del gusto ☐ Si (1); ☐ No (2);
- 103 f. Pérdida del olfato ☐ Si (1); ☐ No (2);
- 103 g. Síntomas generales ☐ Si (1); ☐ No (2);
- 103 h. Otros síntomas ☐ Si (1); ☐ No (2);
- 103 i. Cuáles: _________________

1. **Síntomas relacionados con el tratamiento para la COVID-19:**

- 104 a. ha tenido síntomas relacionados con el tratamiento: ☐ Si (1); ☐ No (2);
- 104 b. Fiebre ☐ Si (1); ☐ No (2);
- 104 c. Rash ☐ Si (1); ☐ No (2);
- 104 d. Diarrea ☐ Si (1); ☐ No (2);
- 104 e. Vómito ☐ Si (1); ☐ No (2);
- 104 f. Lipotimia ☐ Si (1); ☐ No (2);
- 104 g. Escalofríos ☐ Si (1); ☐ No (2);
- 104 h. Dolores musculares ☐ Si (1); ☐ No (2);
- 104 i. Trastornos dispépticos ☐ Si (1); ☐ No (2);
- 104 j. Otros síntomas? _______________

1. **¿Cómo ha transcurrido su enfermedad COVID-19?**

- 105 a. Asintomatica ☐ (1)
- 105 b. Leve ☐ (2)
- 105 c. Severa ☐ (3)
