# Supplementary material for: Development and validation of a questionnaire to assess environmental and lifestyle factors influencing infectious disease outcomes: application to SARS-CoV-2-infected individuals in Cuba
Source: Front Public Health. 2026 Jun 29;14:1805212. doi: 10.3389/fpubh.2026.1805212 (PMC13357406; doi:10.3389/fpubh.2026.1805212)
Supplement: Supplementary file 2 [file Table_2.doc]

**SOLICITUD DE PARTICIPACIÓN COMO EXPERTO EN LA VALIDACIÓN DE UN CUESTIONARIO**

Por su experiencia académica y científica le solicitamos que participe en calidad de “experto(a)” en la validación del cuestionario propuesto para cumplimentar los objetivos del **Proyecto:** Impacto de los factores epigenéticos en la evolución clínica de la COVID 19 en población cubana.

**Nombre del Cuestionario motivo de evaluación:** Cuestionario sobre hábitos, estilos de vida e influencia medioambiental en población cubana

**Antecedentes que motivan la investigación:**

La COVID 19 es una enfermedad declarada pandemia en 2020 que está afectando a todo el planeta incluido nuestro país, Cuba, constituyendo una emergencia de salud. El Instituto de Medicina Tropical “Pedro Kourí“ (IPK), Centro Cubano de Referencia Nacional y Regional para enfermedades virales, está desarrollando estudios de importancia científica y social sobre esta enfermedad. En el presente proyecto se emplea una técnica novedosa, la epigenética, que estudia como nuestros hábitos y el medioambiente pueden influir en los genes y modificar la salud y nuestra respuesta a las enfermedades. Para ello se elaboró un cuestionario con 105 preguntas que abarcan todos los aspectos antes mencionados.

**Criterios de evaluación:**

- Pertinencia: Si la pregunta en el cuestionario se responde con las opciones de respuestas, ¿es relevante para el objetivo de investigación?
- Adecuación: Si el cuestionario está adaptado a las características de las personas a las que se le va a solicitar información ¿está formulado con el lenguaje apropiado y correctamente redactado?
- Organización: Si en las preguntas que integran el cuestionario existe una organización lógica.

Le ofrecemos dos tablas para facilitar la evaluación. Todos los criterios propuestos podrán evaluarse en una escala de 1 a 5. El valor ¨1¨corresponderá al menos pertinente, menos adecuado y menos organizado. Usted debe seleccionar, para cada pregunta del cuestionario y para el cuestionario en su totalidad, la opción que se ajuste a su criterio. También agradeceríamos que nos comunicara sus comentarios. Puede hacerlos a cada una de las preguntas y al cuestionario como un todo utilizando tantas hojas como necesite.

Necesitamos sus valiosas sugerencias, Gracias

Nombre y apellidos del experto(a): __________________________________________

Fecha: _________________ Firma:

ANÁLISIS DEL CUESTIONARIO

| **Preguntas del cuestionario** | **PERTINENCIA** | | | | | **ADECUACUACIÓN** | | | | | **ORGANIZACIÓN** | | | | |
| --- | --- | --- | --- | --- | --- | --- | --- | --- | --- | --- | --- | --- | --- | --- | --- |
|  | **1** | **2** | **3** | **4** | **5** | **1** | **2** | **3** | **4** | **5** | **1** | **2** | **3** | **4** | **5** |
| 1 |  |  |  |  |  |  |  |  |  |  |  |  |  |  |  |
| 2 |  |  |  |  |  |  |  |  |  |  |  |  |  |  |  |
| 3 |  |  |  |  |  |  |  |  |  |  |  |  |  |  |  |
| 4 |  |  |  |  |  |  |  |  |  |  |  |  |  |  |  |
| 5 |  |  |  |  |  |  |  |  |  |  |  |  |  |  |  |
| 6 |  |  |  |  |  |  |  |  |  |  |  |  |  |  |  |
| 7 |  |  |  |  |  |  |  |  |  |  |  |  |  |  |  |
| 8 |  |  |  |  |  |  |  |  |  |  |  |  |  |  |  |
| 9 |  |  |  |  |  |  |  |  |  |  |  |  |  |  |  |
| 10 |  |  |  |  |  |  |  |  |  |  |  |  |  |  |  |
| 11 |  |  |  |  |  |  |  |  |  |  |  |  |  |  |  |
| 12 |  |  |  |  |  |  |  |  |  |  |  |  |  |  |  |
| 13 |  |  |  |  |  |  |  |  |  |  |  |  |  |  |  |
| 14 |  |  |  |  |  |  |  |  |  |  |  |  |  |  |  |
| 15 |  |  |  |  |  |  |  |  |  |  |  |  |  |  |  |
| 16 |  |  |  |  |  |  |  |  |  |  |  |  |  |  |  |
| 17 |  |  |  |  |  |  |  |  |  |  |  |  |  |  |  |
| 18 |  |  |  |  |  |  |  |  |  |  |  |  |  |  |  |
| 19 |  |  |  |  |  |  |  |  |  |  |  |  |  |  |  |
| 20 |  |  |  |  |  |  |  |  |  |  |  |  |  |  |  |
| 21 |  |  |  |  |  |  |  |  |  |  |  |  |  |  |  |
| 22 |  |  |  |  |  |  |  |  |  |  |  |  |  |  |  |
| 23 |  |  |  |  |  |  |  |  |  |  |  |  |  |  |  |
| 24 |  |  |  |  |  |  |  |  |  |  |  |  |  |  |  |
| 25 |  |  |  |  |  |  |  |  |  |  |  |  |  |  |  |
| 26 |  |  |  |  |  |  |  |  |  |  |  |  |  |  |  |
| 27 |  |  |  |  |  |  |  |  |  |  |  |  |  |  |  |
| 28 |  |  |  |  |  |  |  |  |  |  |  |  |  |  |  |
| 29 |  |  |  |  |  |  |  |  |  |  |  |  |  |  |  |
| 30 |  |  |  |  |  |  |  |  |  |  |  |  |  |  |  |
| 31 |  |  |  |  |  |  |  |  |  |  |  |  |  |  |  |
| 32 |  |  |  |  |  |  |  |  |  |  |  |  |  |  |  |
| 33 |  |  |  |  |  |  |  |  |  |  |  |  |  |  |  |
| 34 |  |  |  |  |  |  |  |  |  |  |  |  |  |  |  |
| 35 |  |  |  |  |  |  |  |  |  |  |  |  |  |  |  |
| 36 |  |  |  |  |  |  |  |  |  |  |  |  |  |  |  |
| 37 |  |  |  |  |  |  |  |  |  |  |  |  |  |  |  |
| 38 |  |  |  |  |  |  |  |  |  |  |  |  |  |  |  |
| **Preguntas del cuestionario** | **PERTINENCIA** | | | | | **ADECUACUACIÓN** | | | | | **ORGANIZACIÓN** | | | | |
|  | **1** | **2** | **3** | **4** | **5** | **1** | **2** | **3** | **4** | **5** | **1** | **2** | **3** | **4** | **5** |
| 39 |  |  |  |  |  |  |  |  |  |  |  |  |  |  |  |
| 40 |  |  |  |  |  |  |  |  |  |  |  |  |  |  |  |
| 41 |  |  |  |  |  |  |  |  |  |  |  |  |  |  |  |
| 42 |  |  |  |  |  |  |  |  |  |  |  |  |  |  |  |
| 43 |  |  |  |  |  |  |  |  |  |  |  |  |  |  |  |
| 44 |  |  |  |  |  |  |  |  |  |  |  |  |  |  |  |
| 45 |  |  |  |  |  |  |  |  |  |  |  |  |  |  |  |
| 46 |  |  |  |  |  |  |  |  |  |  |  |  |  |  |  |
| 47 |  |  |  |  |  |  |  |  |  |  |  |  |  |  |  |
| 48 |  |  |  |  |  |  |  |  |  |  |  |  |  |  |  |
| 49 |  |  |  |  |  |  |  |  |  |  |  |  |  |  |  |
| 50 |  |  |  |  |  |  |  |  |  |  |  |  |  |  |  |
| 51 |  |  |  |  |  |  |  |  |  |  |  |  |  |  |  |
| 52 |  |  |  |  |  |  |  |  |  |  |  |  |  |  |  |
| 53 |  |  |  |  |  |  |  |  |  |  |  |  |  |  |  |
| 54 |  |  |  |  |  |  |  |  |  |  |  |  |  |  |  |
| 55 |  |  |  |  |  |  |  |  |  |  |  |  |  |  |  |
| 56 |  |  |  |  |  |  |  |  |  |  |  |  |  |  |  |
| 57 |  |  |  |  |  |  |  |  |  |  |  |  |  |  |  |
| 58 |  |  |  |  |  |  |  |  |  |  |  |  |  |  |  |
| 59 |  |  |  |  |  |  |  |  |  |  |  |  |  |  |  |
| 60 |  |  |  |  |  |  |  |  |  |  |  |  |  |  |  |
| 61 |  |  |  |  |  |  |  |  |  |  |  |  |  |  |  |
| 62 |  |  |  |  |  |  |  |  |  |  |  |  |  |  |  |
| 63 |  |  |  |  |  |  |  |  |  |  |  |  |  |  |  |
| 64 |  |  |  |  |  |  |  |  |  |  |  |  |  |  |  |
| 65 |  |  |  |  |  |  |  |  |  |  |  |  |  |  |  |
| 66 |  |  |  |  |  |  |  |  |  |  |  |  |  |  |  |
| 67 |  |  |  |  |  |  |  |  |  |  |  |  |  |  |  |
| 68 |  |  |  |  |  |  |  |  |  |  |  |  |  |  |  |
| 69 |  |  |  |  |  |  |  |  |  |  |  |  |  |  |  |
| 70 |  |  |  |  |  |  |  |  |  |  |  |  |  |  |  |
| 71 |  |  |  |  |  |  |  |  |  |  |  |  |  |  |  |
| 72 |  |  |  |  |  |  |  |  |  |  |  |  |  |  |  |
| 73 |  |  |  |  |  |  |  |  |  |  |  |  |  |  |  |
| 74 |  |  |  |  |  |  |  |  |  |  |  |  |  |  |  |
| 75 |  |  |  |  |  |  |  |  |  |  |  |  |  |  |  |
| 76 |  |  |  |  |  |  |  |  |  |  |  |  |  |  |  |
| **Preguntas del cuestionario** | **PERTINENCIA** | | | | | **ADECUACUACIÓN** | | | | | **ORGANIZACIÓN** | | | | |
|  | **1** | **2** | **3** | **4** | **5** | **1** | **2** | **3** | **4** | **5** | **1** | **2** | **3** | **4** | **5** |
| 77 |  |  |  |  |  |  |  |  |  |  |  |  |  |  |  |
| 78 |  |  |  |  |  |  |  |  |  |  |  |  |  |  |  |
| 79 |  |  |  |  |  |  |  |  |  |  |  |  |  |  |  |
| 80 |  |  |  |  |  |  |  |  |  |  |  |  |  |  |  |
| 81 |  |  |  |  |  |  |  |  |  |  |  |  |  |  |  |
| 82 |  |  |  |  |  |  |  |  |  |  |  |  |  |  |  |
| 83 |  |  |  |  |  |  |  |  |  |  |  |  |  |  |  |
| 84 |  |  |  |  |  |  |  |  |  |  |  |  |  |  |  |
| 85 |  |  |  |  |  |  |  |  |  |  |  |  |  |  |  |
| 86 |  |  |  |  |  |  |  |  |  |  |  |  |  |  |  |
| 87 |  |  |  |  |  |  |  |  |  |  |  |  |  |  |  |
| 88 |  |  |  |  |  |  |  |  |  |  |  |  |  |  |  |
| 89 |  |  |  |  |  |  |  |  |  |  |  |  |  |  |  |
| 90 |  |  |  |  |  |  |  |  |  |  |  |  |  |  |  |
| 91 |  |  |  |  |  |  |  |  |  |  |  |  |  |  |  |
| 92 |  |  |  |  |  |  |  |  |  |  |  |  |  |  |  |
| 93 |  |  |  |  |  |  |  |  |  |  |  |  |  |  |  |
| 94 |  |  |  |  |  |  |  |  |  |  |  |  |  |  |  |
| 95 |  |  |  |  |  |  |  |  |  |  |  |  |  |  |  |
| 96 |  |  |  |  |  |  |  |  |  |  |  |  |  |  |  |
| 97 |  |  |  |  |  |  |  |  |  |  |  |  |  |  |  |
| 98 |  |  |  |  |  |  |  |  |  |  |  |  |  |  |  |
| 99 |  |  |  |  |  |  |  |  |  |  |  |  |  |  |  |
| 100 |  |  |  |  |  |  |  |  |  |  |  |  |  |  |  |
| 101 |  |  |  |  |  |  |  |  |  |  |  |  |  |  |  |
| 102 |  |  |  |  |  |  |  |  |  |  |  |  |  |  |  |
| 103 |  |  |  |  |  |  |  |  |  |  |  |  |  |  |  |
| 104 |  |  |  |  |  |  |  |  |  |  |  |  |  |  |  |
| 105 |  |  |  |  |  |  |  |  |  |  |  |  |  |  |  |

| **Evaluación integral del cuestionario** | **PERTINENCIA** | | | | | **ADECUACUACIÓN** | | | | | **ORGANIZACIÓN** | | | | |
| --- | --- | --- | --- | --- | --- | --- | --- | --- | --- | --- | --- | --- | --- | --- | --- |
|  | **1** | **2** | **3** | **4** | **5** | **1** | **2** | **3** | **4** | **5** | **1** | **2** | **3** | **4** | **5** |
|  |  |  |  |  |  |  |  |  |  |  |  |  |  |  |  |

Comentarios (puede incluir todas las modificaciones que considere necesarios)
