# Supplementary material for: Development and validation of a questionnaire to assess environmental and lifestyle factors influencing infectious disease outcomes: application to SARS-CoV-2-infected individuals in Cuba
Source: Front Public Health. 2026 Jun 29;14:1805212. doi: 10.3389/fpubh.2026.1805212 (PMC13357406; doi:10.3389/fpubh.2026.1805212)
Supplement: Supplementary file 3 [file Table_3.docx]

Supplementary Material

| Table S3.1: Question-level (n=105) content validity indices for pertinence, adequacy and organization.   \| **Dimensions^a^** \| **item** \| **CVRp** \| **CVIp** \| **Vp(CI)** \| **CVRa** \| **CVIa** \| **Va(CI)** \| **CVRo** \| **CVIo** \| **Vo(CI)** \| \| --- \| --- \| --- \| --- \| --- \| --- \| --- \| --- \| --- \| --- \| --- \| \| Demg \| 1 \| 0.87 \| 0.93 \| 0.95 (0.92;0.97) \| 0.87 \| 0.93 \| 0.97 (0.94;0.98) \| 1.00 \| 1.00 \| 1 (0.99;1) \| \| Demg \| 2 \| 1.00 \| 1.00 \| 0.97 (0.94;0.98) \| 1.00 \| 1.00 \| 0.97 (0.94;0.98) \| 1.00 \| 1.00 \| 1 (0.99;1) \| \| Demg \| 3 \| 1.00 \| 1.00 \| 1 (0.99;1) \| 1.00 \| 1.00 \| 1 (0.99;1) \| 1.00 \| 1.00 \| 1 (0.99;1) \| \| Demg \| 4 \| 1.00 \| 1.00 \| 0.98 (0.96;0.99) \| 1.00 \| 1.00 \| 0.98 (0.96;0.99) \| 1.00 \| 1.00 \| 1 (0.99;1) \| \| Demg \| 5 \| 0.87 \| 0.93 \| 0.97 (0.94;0.98) \| 1.00 \| 1.00 \| 0.98 (0.96;0.99) \| 1.00 \| 1.00 \| 1 (0.99;1) \| \| Demg \| 6 \| 1.00 \| 1.00 \| 0.97 (0.94;0.98) \| 0.87 \| 0.93 \| 0.95 (0.92;0.97) \| 0.87 \| 0.93 \| 0.95 (0.92;0.97) \| \| Demg \| 7 \| 1.00 \| 1.00 \| 0.98 (0.96;0.99) \| 0.87 \| 0.93 \| 0.95 (0.92;0.97) \| 0.87 \| 0.93 \| 0.97 (0.94;0.98) \| \| Demg \| 8 \| 1.00 \| 1.00 \| 0.97 (0.94;0.98) \| 0.87 \| 0.93 \| 0.93 (0.89;0.95) \| 0.87 \| 0.93 \| 0.97 (0.94;0.98) \| \| Demg \| 9 \| 1.00 \| 1.00 \| 0.98 (0.96;0.99) \| 0.73 \| 0.87 \| 0.92 (0.88;0.95) \| 1.00 \| 1.00 \| 0.98 (0.96;0.99) \| \| Demg \| 10 \| 0.33 \| 0.67 \| 0.75 (0.69;0.8) \| 0.60 \| 0.80 \| 0.85 (0.8;0.89) \| 0.87 \| 0.93 \| 0.95 (0.92;0.97) \| \| Pollt \| 11 \| 0.73 \| 0.87 \| 0.87 (0.82;0.91) \| 0.73 \| 0.87 \| 0.88 (0.84;0.91) \| 0.87 \| 0.93 \| 0.9 (0.86;0.93) \| \| CCI \| 12 \| 0.87 \| 0.93 \| 0.95 (0.92;0.97) \| 0.87 \| 0.93 \| 0.95 (0.92;0.97) \| 0.87 \| 0.93 \| 0.95 (0.92;0.97) \| \| CCI \| 13 \| 1.00 \| 1.00 \| 0.98 (0.96;0.99) \| 0.87 \| 0.93 \| 0.92 (0.88;0.95) \| 0.73 \| 0.87 \| 0.9 (0.86;0.93) \| \| outc \| 14 \| 1.00 \| 1.00 \| 1 (0.99;1) \| 0.73 \| 0.87 \| 0.93 (0.89;0.95) \| 0.87 \| 0.93 \| 0.95 (0.92;0.97) \| \| outc \| 15 \| 1.00 \| 1.00 \| 0.95 (0.92;0.97) \| 0.73 \| 0.87 \| 0.87 (0.82;0.91) \| 0.87 \| 0.93 \| 0.93 (0.89;0.95) \| \| CCI \| 16 \| 1.00 \| 1.00 \| 0.98 (0.96;0.99) \| 1.00 \| 1.00 \| 0.95 (0.92;0.97) \| 0.73 \| 0.87 \| 0.92 (0.88;0.95) \| \| CsMed \| 17 \| 0.73 \| 0.87 \| 0.92 (0.88;0.95) \| 0.60 \| 0.80 \| 0.85 (0.8;0.89) \| 0.87 \| 0.93 \| 0.95 (0.92;0.97) \| \| outc \| 18 \| 0.87 \| 0.93 \| 0.95 (0.92;0.97) \| 0.87 \| 0.93 \| 0.87 (0.82;0.91) \| 1.00 \| 1.00 \| 1 (0.99;1) \| \| CsMed \| 19 \| 0.73 \| 0.87 \| 0.9 (0.86;0.93) \| 0.73 \| 0.87 \| 0.9 (0.86;0.93) \| 1.00 \| 1.00 \| 0.95 (0.92;0.97) \| \| CsMed \| 20 \| 0.87 \| 0.93 \| 0.92 (0.88;0.95) \| 0.87 \| 0.93 \| 0.95 (0.92;0.97) \| 1.00 \| 1.00 \| 0.97 (0.94;0.98) \| \| CsNat \| 21 \| 0.87 \| 0.93 \| 0.93 (0.89;0.95) \| 1.00 \| 1.00 \| 0.95 (0.92;0.97) \| 1.00 \| 1.00 \| 0.98 (0.96;0.99) \| \| CsNat \| 22 \| 0.87 \| 0.93 \| 0.95 (0.92;0.97) \| 1.00 \| 1.00 \| 0.97 (0.94;0.98) \| 1.00 \| 1.00 \| 0.98 (0.96;0.99) \| \| CCI \| 23 \| 0.73 \| 0.87 \| 0.93 (0.89;0.95) \| 0.87 \| 0.93 \| 0.95 (0.92;0.97) \| 1.00 \| 1.00 \| 0.98 (0.96;0.99) \| \| CCI \| 24 \| 0.87 \| 0.93 \| 0.92 (0.88;0.95) \| 0.60 \| 0.80 \| 0.83 (0.78;0.87) \| 0.87 \| 0.93 \| 0.95 (0.92;0.97) \| \| CCI \| 25 \| 0.87 \| 0.93 \| 0.97 (0.94;0.98) \| 1.00 \| 1.00 \| 0.95 (0.92;0.97) \| 0.73 \| 0.87 \| 0.92 (0.88;0.95) \| \| CCI \| 26 \| 0.73 \| 0.87 \| 0.9 (0.86;0.93) \| 0.87 \| 0.93 \| 0.92 (0.88;0.95) \| 0.73 \| 0.87 \| 0.92 (0.88;0.95) \| \| CCI \| 27 \| 0.87 \| 0.93 \| 0.92 (0.88;0.95) \| 1.00 \| 1.00 \| 0.93 (0.89;0.95) \| 0.87 \| 0.93 \| 0.93 (0.89;0.95) \| \| Demg \| 28 \| 0.87 \| 0.93 \| 0.95 (0.92;0.97) \| 1.00 \| 1.00 \| 0.98 (0.96;0.99) \| 0.60 \| 0.80 \| 0.87 (0.82;0.91) \| \| Demg \| 29 \| 0.73 \| 0.87 \| 0.87 (0.82;0.91) \| 1.00 \| 1.00 \| 0.98 (0.96;0.99) \| 0.60 \| 0.80 \| 0.87 (0.82;0.91) \| \| CCI \| 30 \| 0.60 \| 0.80 \| 0.85 (0.8;0.89) \| 0.87 \| 0.93 \| 0.93 (0.89;0.95) \| 0.60 \| 0.80 \| 0.82 (0.77;0.86) \| \| Phys \| 31 \| 0.60 \| 0.80 \| 0.87 (0.82;0.91) \| 0.87 \| 0.93 \| 0.93 (0.89;0.95) \| 1.00 \| 1.00 \| 0.95 (0.92;0.97) \| \| Phys \| 32 \| 0.73 \| 0.87 \| 0.88 (0.84;0.91) \| 1.00 \| 1.00 \| 0.97 (0.94;0.98) \| 1.00 \| 1.00 \| 0.97 (0.94;0.98) \| \| Phys \| 33 \| 0.73 \| 0.87 \| 0.9 (0.86;0.93) \| 0.87 \| 0.93 \| 0.93 (0.89;0.95) \| 0.87 \| 0.93 \| 0.92 (0.88;0.95) \| \| Phys \| 34 \| 0.60 \| 0.80 \| 0.85 (0.8;0.89) \| 0.73 \| 0.87 \| 0.85 (0.8;0.89) \| 1.00 \| 1.00 \| 1 (0.99;1) \| \| FreeT \| 35 \| 0.87 \| 0.93 \| 0.97 (0.94;0.98) \| 1.00 \| 1.00 \| 0.97 (0.94;0.98) \| 1.00 \| 1.00 \| 0.98 (0.96;0.99) \| \| FreeT \| 36 \| 0.87 \| 0.93 \| 0.97 (0.94;0.98) \| 1.00 \| 1.00 \| 0.98 (0.96;0.99) \| 1.00 \| 1.00 \| 0.98 (0.96;0.99) \| \| FreeT \| 37 \| 0.87 \| 0.93 \| 0.95 (0.92;0.97) \| 1.00 \| 1.00 \| 0.98 (0.96;0.99) \| 1.00 \| 1.00 \| 0.97 (0.94;0.98) \| \| SelfC \| 38 \| 0.60 \| 0.80 \| 0.88 (0.84;0.91) \| 1.00 \| 1.00 \| 1 (0.99;1) \| 0.87 \| 0.93 \| 0.95 (0.92;0.97) \| \| SelfC \| 39 \| 1.00 \| 1.00 \| 0.98 (0.96;0.99) \| 0.87 \| 0.93 \| 0.92 (0.88;0.95) \| 1.00 \| 1.00 \| 0.97 (0.94;0.98) \| \| SelfC \| 40 \| 0.87 \| 0.93 \| 0.95 (0.92;0.97) \| 0.87 \| 0.93 \| 0.93 (0.89;0.95) \| 0.87 \| 0.93 \| 0.93 (0.89;0.95) \| \| SelfC \| 41 \| 0.87 \| 0.93 \| 0.97 (0.94;0.98) \| 1.00 \| 1.00 \| 0.98 (0.96;0.99) \| 0.87 \| 0.93 \| 0.95 (0.92;0.97) \| \| SelfC \| 42 \| 1.00 \| 1.00 \| 1 (0.99;1) \| 1.00 \| 1.00 \| 0.98 (0.96;0.99) \| 1.00 \| 1.00 \| 0.98 (0.96;0.99) \| \| SelfC \| 43 \| 1.00 \| 1.00 \| 1 (0.99;1) \| 1.00 \| 1.00 \| 0.98 (0.96;0.99) \| 1.00 \| 1.00 \| 0.98 (0.96;0.99) \| \| SelfC \| 44 \| 1.00 \| 1.00 \| 1 (0.99;1) \| 0.87 \| 0.93 \| 0.95 (0.92;0.97) \| 1.00 \| 1.00 \| 0.98 (0.96;0.99) \| \| SelfC \| 45 \| 1.00 \| 1.00 \| 1 (0.99;1) \| 1.00 \| 1.00 \| 0.98 (0.96;0.99) \| 0.87 \| 0.93 \| 0.95 (0.92;0.97) \| \| SelfC \| 46 \| 0.73 \| 0.87 \| 0.9 (0.86;0.93) \| 1.00 \| 1.00 \| 0.98 (0.96;0.99) \| 1.00 \| 1.00 \| 0.98 (0.96;0.99) \| \| SelfC \| 47 \| 0.73 \| 0.87 \| 0.9 (0.86;0.93) \| 1.00 \| 1.00 \| 0.95 (0.92;0.97) \| 1.00 \| 1.00 \| 0.98 (0.96;0.99) \| \| SelfC \| 48 \| 0.87 \| 0.93 \| 0.92 (0.88;0.95) \| 0.87 \| 0.93 \| 0.93 (0.89;0.95) \| 1.00 \| 1.00 \| 0.98 (0.96;0.99) \| \| SelfC \| 49 \| 0.60 \| 0.80 \| 0.87 (0.82;0.91) \| 0.47 \| 0.73 \| 0.8 (0.75;0.84) \| 0.73 \| 0.87 \| 0.87 (0.82;0.91) \| \| SelfC \| 50 \| 0.73 \| 0.87 \| 0.87 (0.82;0.91) \| 0.60 \| 0.80 \| 0.88 (0.84;0.91) \| 0.87 \| 0.93 \| 0.92 (0.88;0.95) \| \| SelfC \| 51 \| 0.87 \| 0.93 \| 0.93 (0.89;0.95) \| 1.00 \| 1.00 \| 0.97 (0.94;0.98) \| 1.00 \| 1.00 \| 0.97 (0.94;0.98) \| \| WrkEn \| 52 \| 0.87 \| 0.93 \| 0.95 (0.92;0.97) \| 1.00 \| 1.00 \| 0.95 (0.92;0.97) \| 1.00 \| 1.00 \| 0.97 (0.94;0.98) \| \| Sednt \| 53 \| 0.87 \| 0.93 \| 0.93 (0.89;0.95) \| 1.00 \| 1.00 \| 0.95 (0.92;0.97) \| 1.00 \| 1.00 \| 0.93 (0.89;0.95) \| \| WrkEn \| 54 \| 0.87 \| 0.93 \| 0.95 (0.92;0.97) \| 1.00 \| 1.00 \| 0.95 (0.92;0.97) \| 1.00 \| 1.00 \| 0.97 (0.94;0.98) \| \| WrkEn \| 55 \| 0.87 \| 0.93 \| 0.97 (0.94;0.98) \| 1.00 \| 1.00 \| 0.98 (0.96;0.99) \| 0.87 \| 0.93 \| 0.95 (0.92;0.97) \| \| StrsV \| 56 \| 0.87 \| 0.93 \| 0.95 (0.92;0.97) \| 1.00 \| 1.00 \| 0.95 (0.92;0.97) \| 0.87 \| 0.93 \| 0.95 (0.92;0.97) \| \| Sednt \| 57 \| 0.87 \| 0.93 \| 0.95 (0.92;0.97) \| 1.00 \| 1.00 \| 0.95 (0.92;0.97) \| 0.87 \| 0.93 \| 0.95 (0.92;0.97) \| \| StrsV \| 58 \| 0.87 \| 0.93 \| 0.97 (0.94;0.98) \| 1.00 \| 1.00 \| 0.95 (0.92;0.97) \| 1.00 \| 1.00 \| 0.97 (0.94;0.98) \| \| StrsV \| 59 \| 1.00 \| 1.00 \| 0.98 (0.96;0.99) \| 0.87 \| 0.93 \| 0.93 (0.89;0.95) \| 1.00 \| 1.00 \| 0.97 (0.94;0.98) \| \| WrkEn \| 60 \| 0.87 \| 0.93 \| 0.92 (0.88;0.95) \| 0.73 \| 0.87 \| 0.87 (0.82;0.91) \| 1.00 \| 1.00 \| 0.98 (0.96;0.99) \| \| WrkEn \| 61 \| 0.87 \| 0.93 \| 0.97 (0.94;0.98) \| 0.87 \| 0.93 \| 0.95 (0.92;0.97) \| 1.00 \| 1.00 \| 0.98 (0.96;0.99) \| \| StrsV \| 62 \| 0.87 \| 0.93 \| 0.95 (0.92;0.97) \| 0.87 \| 0.93 \| 0.95 (0.92;0.97) \| 0.87 \| 0.93 \| 0.92 (0.88;0.95) \| \| StrsV \| 63 \| 0.87 \| 0.93 \| 0.97 (0.94;0.98) \| 1.00 \| 1.00 \| 0.95 (0.92;0.97) \| 0.87 \| 0.93 \| 0.93 (0.89;0.95) \| \| StrsV \| 64 \| 0.87 \| 0.93 \| 0.97 (0.94;0.98) \| 1.00 \| 1.00 \| 0.98 (0.96;0.99) \| 0.87 \| 0.93 \| 0.93 (0.89;0.95) \| \| StrsV \| 65 \| 1.00 \| 1.00 \| 0.97 (0.94;0.98) \| 1.00 \| 1.00 \| 0.97 (0.94;0.98) \| 0.73 \| 0.87 \| 0.88 (0.84;0.91) \| \| Pollt \| 66 \| 0.87 \| 0.93 \| 0.93 (0.89;0.95) \| 0.87 \| 0.93 \| 0.93 (0.89;0.95) \| 0.87 \| 0.93 \| 0.95 (0.92;0.97) \| \| Diet \| 67 \| 0.87 \| 0.93 \| 0.97 (0.94;0.98) \| 1.00 \| 1.00 \| 1 (0.99;1) \| 1.00 \| 1.00 \| 1 (0.99;1) \| \| Diet \| 68 \| 0.87 \| 0.93 \| 0.97 (0.94;0.98) \| 1.00 \| 1.00 \| 0.98 (0.96;0.99) \| 1.00 \| 1.00 \| 1 (0.99;1) \| \| Diet \| 69 \| 0.87 \| 0.93 \| 0.97 (0.94;0.98) \| 1.00 \| 1.00 \| 0.98 (0.96;0.99) \| 1.00 \| 1.00 \| 0.98 (0.96;0.99) \| \| Diet \| 70 \| 0.87 \| 0.93 \| 0.97 (0.94;0.98) \| 1.00 \| 1.00 \| 1 (0.99;1) \| 1.00 \| 1.00 \| 1 (0.99;1) \| \| Diet \| 71 \| 1.00 \| 1.00 \| 0.98 (0.96;0.99) \| 0.87 \| 0.93 \| 0.95 (0.92;0.97) \| 1.00 \| 1.00 \| 1 (0.99;1) \| \| Diet \| 72 \| 1.00 \| 1.00 \| 0.97 (0.94;0.98) \| 0.87 \| 0.93 \| 0.97 (0.94;0.98) \| 1.00 \| 1.00 \| 1 (0.99;1) \| \| Diet \| 73 \| 0.87 \| 0.93 \| 0.95 (0.92;0.97) \| 1.00 \| 1.00 \| 0.98 (0.96;0.99) \| 1.00 \| 1.00 \| 0.98 (0.96;0.99) \| \| Diet \| 74 \| 0.73 \| 0.87 \| 0.92 (0.88;0.95) \| 1.00 \| 1.00 \| 1 (0.99;1) \| 1.00 \| 1.00 \| 1 (0.99;1) \| \| Diet \| 75 \| 0.87 \| 0.93 \| 0.93 (0.89;0.95) \| 1.00 \| 1.00 \| 0.98 (0.96;0.99) \| 1.00 \| 1.00 \| 1 (0.99;1) \| \| Diet \| 76 \| 0.87 \| 0.93 \| 0.97 (0.94;0.98) \| 1.00 \| 1.00 \| 1 (0.99;1) \| 1.00 \| 1.00 \| 1 (0.99;1) \| \| Diet \| 77 \| 0.87 \| 0.93 \| 0.93 (0.89;0.95) \| 1.00 \| 1.00 \| 0.98 (0.96;0.99) \| 1.00 \| 1.00 \| 0.97 (0.94;0.98) \| \| Diet \| 78 \| 0.73 \| 0.87 \| 0.87 (0.82;0.91) \| 0.87 \| 0.93 \| 0.95 (0.92;0.97) \| 1.00 \| 1.00 \| 0.98 (0.96;0.99) \| \| Diet \| 79 \| 0.87 \| 0.93 \| 0.92 (0.88;0.95) \| 1.00 \| 1.00 \| 0.97 (0.94;0.98) \| 1.00 \| 1.00 \| 1 (0.99;1) \| \| Diet \| 80 \| 0.87 \| 0.93 \| 0.93 (0.89;0.95) \| 1.00 \| 1.00 \| 0.98 (0.96;0.99) \| 1.00 \| 1.00 \| 1 (0.99;1) \| \| Diet \| 81 \| 0.87 \| 0.93 \| 0.97 (0.94;0.98) \| 1.00 \| 1.00 \| 1 (0.99;1) \| 1.00 \| 1.00 \| 1 (0.99;1) \| \| Diet \| 82 \| 1.00 \| 1.00 \| 0.95 (0.92;0.97) \| 1.00 \| 1.00 \| 0.98 (0.96;0.99) \| 1.00 \| 1.00 \| 1 (0.99;1) \| \| Diet \| 83 \| 1.00 \| 1.00 \| 1 (0.99;1) \| 1.00 \| 1.00 \| 0.98 (0.96;0.99) \| 1.00 \| 1.00 \| 1 (0.99;1) \| \| CsPsc \| 84 \| 1.00 \| 1.00 \| 1 (0.99;1) \| 0.87 \| 0.93 \| 0.95 (0.92;0.97) \| 1.00 \| 1.00 \| 0.98 (0.96;0.99) \| \| CsPsc \| 85 \| 1.00 \| 1.00 \| 0.98 (0.96;0.99) \| 1.00 \| 1.00 \| 0.98 (0.96;0.99) \| 1.00 \| 1.00 \| 1 (0.99;1) \| \| CsPsc \| 86 \| 0.87 \| 0.93 \| 0.97 (0.94;0.98) \| 0.87 \| 0.93 \| 0.95 (0.92;0.97) \| 0.87 \| 0.93 \| 0.97 (0.94;0.98) \| \| CsPsc \| 87 \| 0.87 \| 0.93 \| 0.97 (0.94;0.98) \| 0.87 \| 0.93 \| 0.95 (0.92;0.97) \| 1.00 \| 1.00 \| 1 (0.99;1) \| \| CsPsc \| 88 \| 1.00 \| 1.00 \| 0.97 (0.94;0.98) \| 1.00 \| 1.00 \| 0.95 (0.92;0.97) \| 1.00 \| 1.00 \| 0.98 (0.96;0.99) \| \| CsPsc \| 89 \| 0.87 \| 0.93 \| 0.97 (0.94;0.98) \| 0.87 \| 0.93 \| 0.93 (0.89;0.95) \| 1.00 \| 1.00 \| 0.98 (0.96;0.99) \| \| Sleep \| 90 \| 0.73 \| 0.87 \| 0.88 (0.84;0.91) \| 1.00 \| 1.00 \| 0.97 (0.94;0.98) \| 1.00 \| 1.00 \| 0.98 (0.96;0.99) \| \| Sleep \| 91 \| 0.87 \| 0.93 \| 0.92 (0.88;0.95) \| 1.00 \| 1.00 \| 0.97 (0.94;0.98) \| 1.00 \| 1.00 \| 1 (0.99;1) \| \| Sleep \| 92 \| 1.00 \| 1.00 \| 0.98 (0.96;0.99) \| 1.00 \| 1.00 \| 1 (0.99;1) \| 1.00 \| 1.00 \| 1 (0.99;1) \| \| Sleep \| 93 \| 1.00 \| 1.00 \| 0.98 (0.96;0.99) \| 1.00 \| 1.00 \| 1 (0.99;1) \| 1.00 \| 1.00 \| 1 (0.99;1) \| \| Sleep \| 94 \| 1.00 \| 1.00 \| 1 (0.99;1) \| 1.00 \| 1.00 \| 1 (0.99;1) \| 1.00 \| 1.00 \| 1 (0.99;1) \| \| Sleep \| 95 \| 1.00 \| 1.00 \| 1 (0.99;1) \| 1.00 \| 1.00 \| 0.98 (0.96;0.99) \| 1.00 \| 1.00 \| 1 (0.99;1) \| \| EconR \| 96 \| 1.00 \| 1.00 \| 1 (0.99;1) \| 1.00 \| 1.00 \| 1 (0.99;1) \| 1.00 \| 1.00 \| 1 (0.99;1) \| \| EconR \| 97 \| 1.00 \| 1.00 \| 0.98 (0.96;0.99) \| 1.00 \| 1.00 \| 1 (0.99;1) \| 1.00 \| 1.00 \| 1 (0.99;1) \| \| EconR \| 98 \| 1.00 \| 1.00 \| 0.98 (0.96;0.99) \| 1.00 \| 1.00 \| 0.98 (0.96;0.99) \| 1.00 \| 1.00 \| 1 (0.99;1) \| \| EconR \| 99 \| 1.00 \| 1.00 \| 0.97 (0.94;0.98) \| 0.87 \| 0.93 \| 0.92 (0.88;0.95) \| 1.00 \| 1.00 \| 0.98 (0.96;0.99) \| \| EconR \| 100 \| 0.73 \| 0.87 \| 0.93 (0.89;0.95) \| 1.00 \| 1.00 \| 0.97 (0.94;0.98) \| 1.00 \| 1.00 \| 1 (0.99;1) \| \| EconR \| 101 \| 0.60 \| 0.80 \| 0.83 (0.78;0.87) \| 0.73 \| 0.87 \| 0.88 (0.84;0.91) \| 0.87 \| 0.93 \| 0.95 (0.92;0.97) \| \| EconR \| 102 \| 1.00 \| 1.00 \| 0.97 (0.94;0.98) \| 0.73 \| 0.87 \| 0.92 (0.88;0.95) \| 1.00 \| 1.00 \| 1 (0.99;1) \| \| EconR \| 103 \| 1.00 \| 1.00 \| 0.97 (0.94;0.98) \| 1.00 \| 1.00 \| 0.98 (0.96;0.99) \| 1.00 \| 1.00 \| 0.98 (0.96;0.99) \| \| Pollt \| 104 \| 1.00 \| 1.00 \| 0.98 (0.96;0.99) \| 1.00 \| 1.00 \| 0.95 (0.92;0.97) \| 1.00 \| 1.00 \| 0.98 (0.96;0.99) \| \| Pollt \| 105 \| 0.87 \| 0.93 \| 0.97 (0.94;0.98) \| 0.87 \| 0.93 \| 0.93 (0.89;0.95) \| 0.87 \| 0.93 \| 0.97 (0.94;0.98) \| \| ^a^ Note: Demg - Demography, Diet - negative nutritional habits, Phys - Physical activity, FreeT - healthy free time activities, Selfc - self care, CsMed - Excessive consumption of medicines, CsNat - consumption of natural products, CPsych - consumption of psychoactive products, EconR - economic restrictions, StrsV - exposure to stress and violence, Sleep - sleep quality, WrkEn - work environment risks, Pollt - pollution, Sednt - sedentary behaviour, CCI - comorbidities. \| \| \| \| \| \| \| \| \| \| \| \| |
| --- | --- | --- | --- | --- | --- | --- | --- | --- | --- | --- | --- | --- | --- | --- | --- | --- | --- | --- | --- | --- | --- | --- | --- | --- | --- | --- | --- | --- | --- | --- | --- | --- | --- | --- | --- | --- | --- | --- | --- | --- | --- | --- | --- | --- | --- | --- | --- | --- | --- | --- | --- | --- | --- | --- | --- | --- | --- | --- | --- | --- | --- | --- | --- | --- | --- | --- | --- | --- | --- | --- | --- | --- | --- | --- | --- | --- | --- | --- | --- | --- | --- | --- | --- | --- | --- | --- | --- | --- | --- | --- | --- | --- | --- | --- | --- | --- | --- | --- | --- | --- | --- | --- | --- | --- | --- | --- | --- | --- | --- | --- | --- | --- | --- | --- | --- | --- | --- | --- | --- | --- | --- | --- | --- | --- | --- | --- | --- | --- | --- | --- | --- | --- | --- | --- | --- | --- | --- | --- | --- | --- | --- | --- | --- | --- | --- | --- | --- | --- | --- | --- | --- | --- | --- | --- | --- | --- | --- | --- | --- | --- | --- | --- | --- | --- | --- | --- | --- | --- | --- | --- | --- | --- | --- | --- | --- | --- | --- | --- | --- | --- | --- | --- | --- | --- | --- | --- | --- | --- | --- | --- | --- | --- | --- | --- | --- | --- | --- | --- | --- | --- | --- | --- | --- | --- | --- | --- | --- | --- | --- | --- | --- | --- | --- | --- | --- | --- | --- | --- | --- | --- | --- | --- | --- | --- | --- | --- | --- | --- | --- | --- | --- | --- | --- | --- | --- | --- | --- | --- | --- | --- | --- | --- | --- | --- | --- | --- | --- | --- | --- | --- | --- | --- | --- | --- | --- | --- | --- | --- | --- | --- | --- | --- | --- | --- | --- | --- | --- | --- | --- | --- | --- | --- | --- | --- | --- | --- | --- | --- | --- | --- | --- | --- | --- | --- | --- | --- | --- | --- | --- | --- | --- | --- | --- | --- | --- | --- | --- | --- | --- | --- | --- | --- | --- | --- | --- | --- | --- | --- | --- | --- | --- | --- | --- | --- | --- | --- | --- | --- | --- | --- | --- | --- | --- | --- | --- | --- | --- | --- | --- | --- | --- | --- | --- | --- | --- | --- | --- | --- | --- | --- | --- | --- | --- | --- | --- | --- | --- | --- | --- | --- | --- | --- | --- | --- | --- | --- | --- | --- | --- | --- | --- | --- | --- | --- | --- | --- | --- | --- | --- | --- | --- | --- | --- | --- | --- | --- | --- | --- | --- | --- | --- | --- | --- | --- | --- | --- | --- | --- | --- | --- | --- | --- | --- | --- | --- | --- | --- | --- | --- | --- | --- | --- | --- | --- | --- | --- | --- | --- | --- | --- | --- | --- | --- | --- | --- | --- | --- | --- | --- | --- | --- | --- | --- | --- | --- | --- | --- | --- | --- | --- | --- | --- | --- | --- | --- | --- | --- | --- | --- | --- | --- | --- | --- | --- | --- | --- | --- | --- | --- | --- | --- | --- | --- | --- | --- | --- | --- | --- | --- | --- | --- | --- | --- | --- | --- | --- | --- | --- | --- | --- | --- | --- | --- | --- | --- | --- | --- | --- | --- | --- | --- | --- | --- | --- | --- | --- | --- | --- | --- | --- | --- | --- | --- | --- | --- | --- | --- | --- | --- | --- | --- | --- | --- | --- | --- | --- | --- | --- | --- | --- | --- | --- | --- | --- | --- | --- | --- | --- | --- | --- | --- | --- | --- | --- | --- | --- | --- | --- | --- | --- | --- | --- | --- | --- | --- | --- | --- | --- | --- | --- | --- | --- | --- | --- | --- | --- | --- | --- | --- | --- | --- | --- | --- | --- | --- | --- | --- | --- | --- | --- | --- | --- | --- | --- | --- | --- | --- | --- | --- | --- | --- | --- | --- | --- | --- | --- | --- | --- | --- | --- | --- | --- | --- | --- | --- | --- | --- | --- | --- | --- | --- | --- | --- | --- | --- | --- | --- | --- | --- | --- | --- | --- | --- | --- | --- | --- | --- | --- | --- | --- | --- | --- | --- | --- | --- | --- | --- | --- | --- | --- | --- | --- | --- | --- | --- | --- | --- | --- | --- | --- | --- | --- | --- | --- | --- | --- | --- | --- | --- | --- | --- | --- | --- | --- | --- | --- | --- | --- | --- | --- | --- | --- | --- | --- | --- | --- | --- | --- | --- | --- | --- | --- | --- | --- | --- | --- | --- | --- | --- | --- | --- | --- | --- | --- | --- | --- | --- | --- | --- | --- | --- | --- | --- | --- | --- | --- | --- | --- | --- | --- | --- | --- | --- | --- | --- | --- | --- | --- | --- | --- | --- | --- | --- | --- | --- | --- | --- | --- | --- | --- | --- | --- | --- | --- | --- | --- | --- | --- | --- | --- | --- | --- | --- | --- | --- | --- | --- | --- | --- | --- | --- | --- | --- | --- | --- | --- | --- | --- | --- | --- | --- | --- | --- | --- | --- | --- | --- | --- | --- | --- | --- | --- | --- | --- | --- | --- | --- | --- | --- | --- | --- | --- | --- | --- | --- | --- | --- | --- | --- | --- | --- | --- | --- | --- | --- | --- | --- | --- | --- | --- | --- | --- | --- | --- | --- | --- | --- | --- | --- | --- | --- | --- | --- | --- | --- | --- | --- | --- | --- | --- | --- | --- | --- | --- | --- | --- | --- | --- | --- | --- | --- | --- | --- | --- | --- | --- | --- | --- | --- | --- | --- | --- | --- | --- | --- | --- | --- | --- | --- | --- | --- | --- | --- | --- | --- | --- | --- | --- | --- | --- | --- | --- | --- | --- | --- | --- | --- | --- | --- | --- | --- | --- | --- | --- | --- | --- | --- | --- | --- | --- | --- | --- | --- | --- | --- | --- | --- | --- | --- | --- | --- | --- | --- | --- | --- | --- | --- | --- | --- | --- | --- | --- | --- | --- | --- | --- | --- | --- | --- | --- | --- | --- | --- | --- | --- | --- | --- | --- | --- | --- | --- | --- | --- | --- | --- | --- | --- | --- | --- | --- | --- | --- | --- | --- | --- | --- | --- | --- | --- | --- | --- | --- | --- | --- | --- | --- | --- | --- | --- | --- | --- | --- | --- | --- | --- | --- | --- | --- | --- | --- | --- | --- | --- | --- | --- | --- | --- | --- | --- | --- | --- | --- | --- | --- | --- | --- | --- | --- | --- | --- | --- | --- | --- | --- | --- | --- | --- | --- | --- | --- | --- | --- | --- | --- | --- | --- | --- | --- | --- | --- | --- | --- | --- | --- | --- | --- | --- | --- | --- | --- | --- | --- | --- | --- | --- | --- | --- | --- | --- | --- | --- | --- | --- | --- | --- | --- | --- | --- | --- | --- | --- | --- | --- | --- | --- | --- | --- | --- | --- | --- | --- | --- | --- | --- | --- | --- | --- | --- | --- | --- | --- | --- | --- | --- | --- | --- | --- | --- | --- | --- | --- | --- | --- | --- | --- | --- | --- | --- | --- | --- | --- | --- | --- | --- | --- | --- | --- | --- | --- | --- | --- | --- | --- | --- | --- | --- | --- | --- | --- | --- | --- | --- | --- | --- | --- | --- | --- | --- | --- | --- | --- | --- | --- | --- | --- | --- | --- | --- | --- | --- | --- | --- | --- | --- | --- | --- | --- | --- | --- | --- | --- | --- | --- | --- | --- | --- | --- | --- | --- | --- | --- | --- | --- | --- | --- | --- | --- | --- | --- | --- | --- | --- | --- | --- | --- | --- | --- | --- | --- | --- | --- | --- | --- | --- | --- | --- | --- | --- | --- | --- | --- | --- | --- | --- | --- | --- | --- | --- | --- | --- | --- | --- | --- | --- | --- | --- | --- | --- | --- | --- | --- | --- | --- | --- | --- | --- | --- | --- | --- | --- | --- | --- | --- | --- | --- | --- | --- | --- |
