# Supplementary material for: Development and validation of a questionnaire to assess environmental and lifestyle factors influencing infectious disease outcomes: application to SARS-CoV-2-infected individuals in Cuba
Source: Front Public Health. 2026 Jun 29;14:1805212. doi: 10.3389/fpubh.2026.1805212 (PMC13357406; doi:10.3389/fpubh.2026.1805212)
Supplement: Supplementary file 4 [file Table_4.docx]

Supplementary Material

| Table S3.2: Item level (n=244) test-retest reliability - Cohen’s kappa, Fleiss kappa, Pearson’s correlation coefficient, Spearman’s rank correlation coefficient and Intra-class correlation coefficient (ICC). Normality test (Shapiro test) for numeric items.   \| **Dimensions^a^** \| **item** \| **type of variable** \| **nominal_ordinal** \| **Shapiro^b^** \| **Sh.p.value^c^** \| **Cohens_kappa^d^** \| **Fleiss_kappa^e^** \| **Pearsons_R^f^** \| **Spearmans_rho^g^** \| **ICC^h^** \| \| --- \| --- \| --- \| --- \| --- \| --- \| --- \| --- \| --- \| --- \| --- \| \| CCI \| enf_cron_alergia \| categorical \| dicot \| NA \| NA \| 0.86 \| NA \| NA \| NA \| NA \| \| CCI \| enf_cron_asma \| categorical \| dicot \| NA \| NA \| 0.90 \| NA \| NA \| NA \| NA \| \| CCI \| enf_cron_cancer \| categorical \| dicot \| NA \| NA \| 1.00 \| NA \| NA \| NA \| NA \| \| CCI \| enf_cron_coltri_alto \| categorical \| dicot \| NA \| NA \| 1.00 \| NA \| NA \| NA \| NA \| \| CCI \| enf_cron_diabetes \| categorical \| dicot \| NA \| NA \| 1.00 \| NA \| NA \| NA \| NA \| \| CCI \| enf_cron_hipert \| categorical \| dicot \| NA \| NA \| 1.00 \| NA \| NA \| NA \| NA \| \| CCI \| enf_cron_otra \| categorical \| dicot \| NA \| NA \| 0.84 \| NA \| NA \| NA \| NA \| \| CCI \| enf_cronica \| categorical \| dicot \| NA \| NA \| 0.93 \| NA \| NA \| NA \| NA \| \| CPsych \| cons_psicofarmaco \| categorical \| dicot \| NA \| NA \| 0.95 \| NA \| NA \| NA \| NA \| \| CPsych \| cons_sust_psicoact \| categorical \| dicot \| NA \| NA \| NA \| NA \| NA \| NA \| NA \| \| CPsych \| cuantas_v_alcohol_sem \| categorical \| ordinal \| NA \| NA \| NA \| 1.00 \| NA \| NA \| NA \| \| CPsych \| fuma \| categorical \| dicot \| NA \| NA \| 1.00 \| NA \| NA \| NA \| NA \| \| CPsych \| fuma_m10cdia \| categorical \| dicot \| NA \| NA \| 1.00 \| NA \| NA \| NA \| NA \| \| CPsych \| permite_fumen \| categorical \| dicot \| NA \| NA \| 0.96 \| NA \| NA \| NA \| NA \| \| CVD_CP \| covid19_evolucion \| categorical \| dicot \| NA \| NA \| 1.00 \| NA \| NA \| NA \| NA \| \| CVD_CP \| covid19_sintomas \| categorical \| dicot \| NA \| NA \| NA \| NA \| NA \| NA \| NA \| \| CVD_CP \| covid19_sintomas_disnea \| categorical \| dicot \| NA \| NA \| 0.90 \| NA \| NA \| NA \| NA \| \| CVD_CP \| covid19_sintomas_fiebre \| categorical \| dicot \| NA \| NA \| 0.95 \| NA \| NA \| NA \| NA \| \| CVD_CP \| covid19_sintomas_generales \| categorical \| dicot \| NA \| NA \| 1.00 \| NA \| NA \| NA \| NA \| \| CVD_CP \| covid19_sintomas_odinofagia \| categorical \| dicot \| NA \| NA \| 1.00 \| NA \| NA \| NA \| NA \| \| CVD_CP \| covid19_sintomas_otros \| categorical \| dicot \| NA \| NA \| 0.86 \| NA \| NA \| NA \| NA \| \| CVD_CP \| covid19_sintomas_otros_cual \| categorical \| NA \| NA \| NA \| NA \| NA \| NA \| NA \| NA \| \| CVD_CP \| covid19_sintomas_perdgusto \| categorical \| dicot \| NA \| NA \| 0.95 \| NA \| NA \| NA \| NA \| \| CVD_CP \| covid19_sintomas_perdolfato \| categorical \| dicot \| NA \| NA \| 0.91 \| NA \| NA \| NA \| NA \| \| CVD_CP \| covid19_sintomas_tos \| categorical \| dicot \| NA \| NA \| 0.95 \| NA \| NA \| NA \| NA \| \| CVD_CP \| medic_covid_sintom_diarrea \| categorical \| dicot \| NA \| NA \| 0.96 \| NA \| NA \| NA \| NA \| \| CVD_CP \| medic_covid_sintom_dolor_musc \| categorical \| dicot \| NA \| NA \| 1.00 \| NA \| NA \| NA \| NA \| \| CVD_CP \| medic_covid_sintom_escalof \| categorical \| dicot \| NA \| NA \| 1.00 \| NA \| NA \| NA \| NA \| \| CVD_CP \| medic_covid_sintom_fiebre \| categorical \| dicot \| NA \| NA \| 0.92 \| NA \| NA \| NA \| NA \| \| CVD_CP \| medic_covid_sintom_lipotimia \| categorical \| dicot \| NA \| NA \| NA \| NA \| NA \| NA \| NA \| \| CVD_CP \| medic_covid_sintom_rash \| categorical \| dicot \| NA \| NA \| NA \| NA \| NA \| NA \| NA \| \| CVD_CP \| medic_covid_sintom_tras_disp \| categorical \| dicot \| NA \| NA \| NA \| NA \| NA \| NA \| NA \| \| CVD_CP \| medic_covid_sintom_vomito \| categorical \| dicot \| NA \| NA \| 0.88 \| NA \| NA \| NA \| NA \| \| CVD_CP \| medic_covid_sintomas \| categorical \| dicot \| NA \| NA \| 0.83 \| NA \| NA \| NA \| NA \| \| CVD_CP \| medic_covid_sintomas_cual \| categorical \| NA \| NA \| NA \| NA \| NA \| NA \| NA \| NA \| \| CVD_CP \| medic_covid_sintomas_sintom \| categorical \| NA \| NA \| NA \| NA \| NA \| NA \| NA \| NA \| \| CsMed \| muchos_medicam \| categorical \| dicot \| NA \| NA \| 0.87 \| NA \| NA \| NA \| NA \| \| CsMed \| se_automedica \| categorical \| dicot \| NA \| NA \| 0.89 \| NA \| NA \| NA \| NA \| \| CsMed \| toma_medicam \| categorical \| dicot \| NA \| NA \| 0.07 \| NA \| NA \| NA \| NA \| \| CsNat \| mnt \| categorical \| dicot \| NA \| NA \| 1.00 \| NA \| NA \| NA \| NA \| \| CsNat \| suplem_antioxi \| categorical \| dicot \| NA \| NA \| NA \| NA \| NA \| NA \| NA \| \| CsNat \| suplem_nutric \| categorical \| dicot \| NA \| NA \| 0.91 \| NA \| NA \| NA \| NA \| \| CsNat \| suplem_vitam \| categorical \| dicot \| NA \| NA \| 1.00 \| NA \| NA \| NA \| NA \| \| CsNat \| suplemento \| categorical \| dicot \| NA \| NA \| 1.00 \| NA \| NA \| NA \| NA \| \| Demog \| ciudad_pueblo \| categorical \| politom \| NA \| NA \| 0.76 \| NA \| NA \| NA \| NA \| \| Demog \| color_piel \| categorical \| politom \| NA \| NA \| 1.00 \| NA \| NA \| NA \| NA \| \| Demog \| edad \| numeric \| NA \| 0.98 \| 0.33 \| NA \| NA \| 1 \| NA \| 1.00 \| \| Demog \| en_la_ciudad_2018 \| categorical \| dicot \| NA \| NA \| 1.00 \| NA \| NA \| NA \| NA \| \| Demog \| estado_civil \| categorical \| politom \| NA \| NA \| 1.00 \| NA \| NA \| NA \| NA \| \| Demog \| estatura \| numeric \| NA \| 0.96 \| 0.06 \| NA \| NA \| 1 \| NA \| 1.00 \| \| Demog \| num_hijos \| numeric \| NA \| 0.89 \| 0.00 \| NA \| NA \| NA \| 1.00 \| 1.00 \| \| Demog \| ocup_profesional_tipo \| categorical \| politom \| NA \| NA \| 0.86 \| NA \| NA \| NA \| NA \| \| Demog \| ocupacion \| categorical \| politom \| NA \| NA \| 1.00 \| NA \| NA \| NA \| NA \| \| Demog \| peso \| numeric \| NA \| 0.98 \| 0.28 \| NA \| NA \| 1 \| NA \| 1.00 \| \| Demog \| sexo \| categorical \| dicot \| NA \| NA \| 1.00 \| NA \| NA \| NA \| NA \| \| Demog \| tiene_hijos \| categorical \| dicot \| NA \| NA \| 1.00 \| NA \| NA \| NA \| NA \| \| Diet \| ahumados_chorizos \| categorical \| dicot \| NA \| NA \| 1.00 \| NA \| NA \| NA \| NA \| \| Diet \| ahumados_jamon \| categorical \| dicot \| NA \| NA \| 1.00 \| NA \| NA \| NA \| NA \| \| Diet \| ahumados_lomo \| categorical \| dicot \| NA \| NA \| 1.00 \| NA \| NA \| NA \| NA \| \| Diet \| ahumados_m1vps \| categorical \| dicot \| NA \| NA \| 1.00 \| NA \| NA \| NA \| NA \| \| Diet \| ahumados_salchichas \| categorical \| dicot \| NA \| NA \| 1.00 \| NA \| NA \| NA \| NA \| \| Diet \| alim_dulces \| categorical \| dicot \| NA \| NA \| 0.92 \| NA \| NA \| NA \| NA \| \| Diet \| almuerzo \| categorical \| dicot \| NA \| NA \| 0.79 \| NA \| NA \| NA \| NA \| \| Diet \| cafe_con_azucar \| categorical \| dicot \| NA \| NA \| 1.00 \| NA \| NA \| NA \| NA \| \| Diet \| cantid_agua_toma_dia \| categorical \| ordinal \| NA \| NA \| NA \| 1.00 \| NA \| NA \| NA \| \| Diet \| cena \| categorical \| dicot \| NA \| NA \| 0.00 \| NA \| NA \| NA \| NA \| \| Diet \| com_rapida_m3vps \| categorical \| dicot \| NA \| NA \| 0.93 \| NA \| NA \| NA \| NA \| \| Diet \| comid_horario_regul \| categorical \| dicot \| NA \| NA \| 0.89 \| NA \| NA \| NA \| NA \| \| Diet \| condim_deshidratado \| categorical \| dicot \| NA \| NA \| 0.00 \| NA \| NA \| NA \| NA \| \| Diet \| condim_naturales \| categorical \| dicot \| NA \| NA \| 0.48 \| NA \| NA \| NA \| NA \| \| Diet \| condim_procesados \| categorical \| dicot \| NA \| NA \| 0.97 \| NA \| NA \| NA \| NA \| \| Diet \| condim_sal \| categorical \| dicot \| NA \| NA \| 0.84 \| NA \| NA \| NA \| NA \| \| Diet \| condimento \| categorical \| dicot \| NA \| NA \| -0.03 \| NA \| NA \| NA \| NA \| \| Diet \| cons_dulces_helados \| categorical \| dicot \| NA \| NA \| 0.88 \| NA \| NA \| NA \| NA \| \| Diet \| cons_refr_enlat \| categorical \| dicot \| NA \| NA \| 1.00 \| NA \| NA \| NA \| NA \| \| Diet \| cuantas_tazas_cafe \| numeric \| NA \| 0.90 \| 0.00 \| NA \| NA \| NA \| 0.93 \| 0.92 \| \| Diet \| cuantas_v_carne \| categorical \| ordinal \| NA \| NA \| NA \| 1.00 \| NA \| NA \| NA \| \| Diet \| desayuna_antes \| categorical \| dicot \| NA \| NA \| 0.92 \| NA \| NA \| NA \| NA \| \| Diet \| desayuno \| categorical \| dicot \| NA \| NA \| 0.87 \| NA \| NA \| NA \| NA \| \| Diet \| dieta_p_perd_peso \| categorical \| dicot \| NA \| NA \| 0.91 \| NA \| NA \| NA \| NA \| \| Diet \| merienda_desp_cena \| categorical \| dicot \| NA \| NA \| 0.93 \| NA \| NA \| NA \| NA \| \| Diet \| merienda_manana \| categorical \| dicot \| NA \| NA \| 0.93 \| NA \| NA \| NA \| NA \| \| Diet \| merienda_tarde \| categorical \| dicot \| NA \| NA \| 0.93 \| NA \| NA \| NA \| NA \| \| Diet \| mucha_sal \| categorical \| dicot \| NA \| NA \| 1.00 \| NA \| NA \| NA \| NA \| \| Diet \| tipo_carne_carnero \| categorical \| dicot \| NA \| NA \| 1.00 \| NA \| NA \| NA \| NA \| \| Diet \| tipo_carne_cerdo \| categorical \| dicot \| NA \| NA \| 0.85 \| NA \| NA \| NA \| NA \| \| Diet \| tipo_carne_pescado \| categorical \| dicot \| NA \| NA \| 0.93 \| NA \| NA \| NA \| NA \| \| Diet \| tipo_carne_pollo \| categorical \| dicot \| NA \| NA \| -0.02 \| NA \| NA \| NA \| NA \| \| Diet \| tipo_carne_res \| categorical \| dicot \| NA \| NA \| 0.93 \| NA \| NA \| NA \| NA \| \| Diet \| toma_cafe \| categorical \| dicot \| NA \| NA \| 0.96 \| NA \| NA \| NA \| NA \| \| Diet \| x1vs_carne \| categorical \| dicot \| NA \| NA \| 0.00 \| NA \| NA \| NA \| NA \| \| Diet \| x1vs_carne_cerdo \| categorical \| dicot \| NA \| NA \| 0.84 \| NA \| NA \| NA \| NA \| \| Diet \| x1vs_cereales \| categorical \| dicot \| NA \| NA \| 0.96 \| NA \| NA \| NA \| NA \| \| Diet \| x1vs_chicharrones \| categorical \| dicot \| NA \| NA \| 1.00 \| NA \| NA \| NA \| NA \| \| Diet \| x1vs_frutas \| categorical \| dicot \| NA \| NA \| 1.00 \| NA \| NA \| NA \| NA \| \| Diet \| x1vs_granos \| categorical \| dicot \| NA \| NA \| 0.79 \| NA \| NA \| NA \| NA \| \| Diet \| x1vs_huevo \| categorical \| dicot \| NA \| NA \| 0.71 \| NA \| NA \| NA \| NA \| \| Diet \| x1vs_lacteos \| categorical \| dicot \| NA \| NA \| 1.00 \| NA \| NA \| NA \| NA \| \| Diet \| x1vs_mantequilla \| categorical \| dicot \| NA \| NA \| 0.90 \| NA \| NA \| NA \| NA \| \| Diet \| x1vs_mayonesa \| categorical \| dicot \| NA \| NA \| 0.83 \| NA \| NA \| NA \| NA \| \| Diet \| x1vs_panes \| categorical \| dicot \| NA \| NA \| 0.86 \| NA \| NA \| NA \| NA \| \| Diet \| x1vs_pescado \| categorical \| dicot \| NA \| NA \| 0.93 \| NA \| NA \| NA \| NA \| \| Diet \| x1vs_queso_crema \| categorical \| dicot \| NA \| NA \| 0.86 \| NA \| NA \| NA \| NA \| \| Diet \| x1vs_salsas \| categorical \| dicot \| NA \| NA \| 1.00 \| NA \| NA \| NA \| NA \| \| Diet \| x1vs_vegetales \| categorical \| dicot \| NA \| NA \| 1.00 \| NA \| NA \| NA \| NA \| \| EconR \| cant_edad_15a24 \| numeric \| NA \| 0.43 \| 0.00 \| NA \| NA \| NA \| 1.00 \| 1.00 \| \| EconR \| cant_edad_25a44 \| numeric \| NA \| 0.39 \| 0.00 \| NA \| NA \| NA \| 1.00 \| 1.00 \| \| EconR \| cant_edad_45a64 \| numeric \| NA \| 0.56 \| 0.00 \| NA \| NA \| NA \| 1.00 \| 1.00 \| \| EconR \| cant_edad_5a14 \| numeric \| NA \| 0.61 \| 0.00 \| NA \| NA \| NA \| 1.00 \| 1.00 \| \| EconR \| cant_edad_65ym \| numeric \| NA \| 0.53 \| 0.00 \| NA \| NA \| NA \| 1.00 \| 1.00 \| \| EconR \| cant_edad_m5a \| numeric \| NA \| NA \| NA \| NA \| NA \| NA \| NA \| NA \| \| EconR \| cant_escol_pre_tec \| numeric \| NA \| 0.55 \| 0.00 \| NA \| NA \| NA \| 0.93 \| 0.95 \| \| EconR \| cant_escol_prim \| numeric \| NA \| 0.63 \| 0.00 \| NA \| NA \| NA \| 1.00 \| 1.00 \| \| EconR \| cant_escol_sec \| numeric \| NA \| 0.66 \| 0.00 \| NA \| NA \| NA \| 1.00 \| 1.00 \| \| EconR \| cant_escol_univ \| numeric \| NA \| 0.65 \| 0.00 \| NA \| NA \| NA \| 1.00 \| 1.00 \| \| EconR \| cant_habitac_vivienda \| numeric \| NA \| 0.94 \| 0.01 \| NA \| NA \| NA \| 1.00 \| 1.00 \| \| EconR \| cant_ocup_amacasa \| numeric \| NA \| 0.43 \| 0.00 \| NA \| NA \| NA \| 1.00 \| 1.00 \| \| EconR \| cant_ocup_campesino \| numeric \| NA \| NA \| NA \| NA \| NA \| NA \| NA \| NA \| \| EconR \| cant_ocup_desocup \| numeric \| NA \| NA \| NA \| NA \| NA \| NA \| NA \| NA \| \| EconR \| cant_ocup_estudiante \| numeric \| NA \| 0.60 \| 0.00 \| NA \| NA \| NA \| 1.00 \| 1.00 \| \| EconR \| cant_ocup_militar \| numeric \| NA \| NA \| NA \| NA \| NA \| NA \| NA \| NA \| \| EconR \| cant_ocup_obrero \| numeric \| NA \| 0.63 \| 0.00 \| NA \| NA \| NA \| 1.00 \| 1.00 \| \| EconR \| cant_ocup_otro \| numeric \| NA \| NA \| NA \| NA \| NA \| NA \| NA \| NA \| \| EconR \| cant_ocup_profesional \| numeric \| NA \| 0.66 \| 0.00 \| NA \| NA \| NA \| 1.00 \| 1.00 \| \| EconR \| cant_ocup_tcp \| numeric \| NA \| NA \| NA \| NA \| NA \| NA \| NA \| NA \| \| EconR \| cant_personas_vivienda \| numeric \| NA \| 0.90 \| 0.00 \| NA \| NA \| NA \| 1.00 \| 1.00 \| \| EconR \| cant_sexo_fem \| numeric \| NA \| 0.78 \| 0.00 \| NA \| NA \| NA \| 1.00 \| 1.00 \| \| EconR \| cant_sexo_masc \| numeric \| NA \| 0.81 \| 0.00 \| NA \| NA \| NA \| 0.98 \| 0.99 \| \| EconR \| equipos_viv_aireac \| categorical \| dicot \| NA \| NA \| 1.00 \| NA \| NA \| NA \| NA \| \| EconR \| equipos_viv_auto_moto \| categorical \| dicot \| NA \| NA \| 1.00 \| NA \| NA \| NA \| NA \| \| EconR \| equipos_viv_batidora \| categorical \| dicot \| NA \| NA \| 1.00 \| NA \| NA \| NA \| NA \| \| EconR \| equipos_viv_bicicleta \| categorical \| dicot \| NA \| NA \| 1.00 \| NA \| NA \| NA \| NA \| \| EconR \| equipos_viv_cocina_gas \| categorical \| dicot \| NA \| NA \| 0.88 \| NA \| NA \| NA \| NA \| \| EconR \| equipos_viv_comput \| categorical \| dicot \| NA \| NA \| 1.00 \| NA \| NA \| NA \| NA \| \| EconR \| equipos_viv_lavadora \| categorical \| dicot \| NA \| NA \| NA \| NA \| NA \| NA \| NA \| \| EconR \| equipos_viv_microwav \| categorical \| dicot \| NA \| NA \| 1.00 \| NA \| NA \| NA \| NA \| \| EconR \| equipos_viv_refrig \| categorical \| dicot \| NA \| NA \| NA \| NA \| NA \| NA \| NA \| \| EconR \| equipos_viv_tv \| categorical \| dicot \| NA \| NA \| 1.00 \| NA \| NA \| NA \| NA \| \| EconR \| equipos_viv_tv_plana \| categorical \| dicot \| NA \| NA \| 1.00 \| NA \| NA \| NA \| NA \| \| EconR \| equipos_viv_ventilador \| categorical \| dicot \| NA \| NA \| 1.00 \| NA \| NA \| NA \| NA \| \| EconR \| frecuenca_agua \| categorical \| ordinal \| NA \| NA \| NA \| 1.00 \| NA \| NA \| NA \| \| EconR \| fuente_agua_acued \| categorical \| dicot \| NA \| NA \| 0.87 \| NA \| NA \| NA \| NA \| \| EconR \| fuente_agua_pozo \| categorical \| dicot \| NA \| NA \| 0.96 \| NA \| NA \| NA \| NA \| \| EconR \| fuente_agua_rio \| categorical \| dicot \| NA \| NA \| NA \| NA \| NA \| NA \| NA \| \| EconR \| gastos_fam_ano_cuc \| categorical \| ordinal \| NA \| NA \| NA \| 0.94 \| NA \| NA \| NA \| \| EconR \| gastos_fam_ano_mn \| categorical \| ordinal \| NA \| NA \| NA \| 0.98 \| NA \| NA \| NA \| \| EconR \| met_desinf_hervir \| categorical \| dicot \| NA \| NA \| 1.00 \| NA \| NA \| NA \| NA \| \| EconR \| met_desinf_hipoclor \| categorical \| dicot \| NA \| NA \| 0.97 \| NA \| NA \| NA \| NA \| \| EconR \| metodo_desinf_agua \| categorical \| dicot \| NA \| NA \| 0.90 \| NA \| NA \| NA \| NA \| \| FreeT \| pref_ami_fami \| categorical \| dicot \| NA \| NA \| 0.85 \| NA \| NA \| NA \| NA \| \| FreeT \| pref_cine \| categorical \| dicot \| NA \| NA \| 1.00 \| NA \| NA \| NA \| NA \| \| FreeT \| pref_deporte \| categorical \| dicot \| NA \| NA \| 1.00 \| NA \| NA \| NA \| NA \| \| FreeT \| pref_estudiar \| categorical \| dicot \| NA \| NA \| 1.00 \| NA \| NA \| NA \| NA \| \| FreeT \| pref_jugaconnh \| categorical \| dicot \| NA \| NA \| 1.00 \| NA \| NA \| NA \| NA \| \| FreeT \| pref_leer \| categorical \| dicot \| NA \| NA \| 0.96 \| NA \| NA \| NA \| NA \| \| FreeT \| pref_musica \| categorical \| dicot \| NA \| NA \| 0.80 \| NA \| NA \| NA \| NA \| \| FreeT \| pref_nada \| categorical \| dicot \| NA \| NA \| 0.95 \| NA \| NA \| NA \| NA \| \| FreeT \| pref_pasear \| categorical \| dicot \| NA \| NA \| 0.85 \| NA \| NA \| NA \| NA \| \| FreeT \| pref_tv \| categorical \| dicot \| NA \| NA \| 0.91 \| NA \| NA \| NA \| NA \| \| FreeT \| t_libre_dia \| categorical \| ordinal \| NA \| NA \| NA \| 0.91 \| NA \| NA \| NA \| \| FreeT \| t_libre_semana \| categorical \| ordinal \| NA \| NA \| NA \| 0.91 \| NA \| NA \| NA \| \| General \| edad_madre \| categorical \| ordinal \| NA \| NA \| NA \| 1.00 \| NA \| NA \| NA \| \| General \| padres_alcohol_diariam \| categorical \| dicot \| NA \| NA \| 1.00 \| NA \| NA \| NA \| NA \| \| General \| padres_fuman \| categorical \| dicot \| NA \| NA \| 1.00 \| NA \| NA \| NA \| NA \| \| General \| resid_madre \| categorical \| dicot \| NA \| NA \| 0.96 \| NA \| NA \| NA \| NA \| \| General \| resid_padre \| categorical \| dicot \| NA \| NA \| 1.00 \| NA \| NA \| NA \| NA \| \| Health \| antec_arbovir \| categorical \| dicot \| NA \| NA \| 0.77 \| NA \| NA \| NA \| NA \| \| Health \| antec_cirugia \| categorical \| dicot \| NA \| NA \| 1.00 \| NA \| NA \| NA \| NA \| \| Health \| apf_arbovir \| categorical \| dicot \| NA \| NA \| 0.96 \| NA \| NA \| NA \| NA \| \| Health \| apf_cron_alergia \| categorical \| dicot \| NA \| NA \| 1.00 \| NA \| NA \| NA \| NA \| \| Health \| apf_cron_asma \| categorical \| dicot \| NA \| NA \| 1.00 \| NA \| NA \| NA \| NA \| \| Health \| apf_cron_cancer \| categorical \| dicot \| NA \| NA \| 0.83 \| NA \| NA \| NA \| NA \| \| Health \| apf_cron_coltri_alto \| categorical \| dicot \| NA \| NA \| 1.00 \| NA \| NA \| NA \| NA \| \| Health \| apf_cron_diabetes \| categorical \| dicot \| NA \| NA \| 0.96 \| NA \| NA \| NA \| NA \| \| Health \| apf_cron_hipert \| categorical \| dicot \| NA \| NA \| 1.00 \| NA \| NA \| NA \| NA \| \| Health \| apf_cron_otra \| categorical \| NA \| NA \| NA \| NA \| NA \| NA \| NA \| NA \| \| Health \| apf_enf_cron \| categorical \| dicot \| NA \| NA \| 0.45 \| NA \| NA \| NA \| NA \| \| Health \| arbov_ano \| categorical \| politom \| NA \| NA \| 1.00 \| NA \| NA \| NA \| NA \| \| Health \| arbov_hospitalizado \| categorical \| dicot \| NA \| NA \| 1.00 \| NA \| NA \| NA \| NA \| \| Health \| arbov_mas10d \| categorical \| dicot \| NA \| NA \| 1.00 \| NA \| NA \| NA \| NA \| \| Health \| arbov_sint_m3m \| categorical \| dicot \| NA \| NA \| 1.00 \| NA \| NA \| NA \| NA \| \| Health \| calif_salud \| categorical \| ordinal \| NA \| NA \| NA \| 0.95 \| NA \| NA \| NA \| \| Health \| cual_sint_m3m \| categorical \| NA \| NA \| NA \| NA \| NA \| NA \| NA \| NA \| \| Health \| hijo_den_grave \| categorical \| dicot \| NA \| NA \| 0.65 \| NA \| NA \| NA \| NA \| \| Health \| padecim_ult_mes \| categorical \| dicot \| NA \| NA \| 0.00 \| NA \| NA \| NA \| NA \| \| Phys \| ej_fis_supervis_entrenador \| categorical \| dicot \| NA \| NA \| 0.91 \| NA \| NA \| NA \| NA \| \| Phys \| ej_fis_supervis_fisiot \| categorical \| dicot \| NA \| NA \| NA \| NA \| NA \| NA \| NA \| \| Phys \| ejerc_fisico_cardio \| categorical \| dicot \| NA \| NA \| 1.00 \| NA \| NA \| NA \| NA \| \| Phys \| ejerc_fisico_relaj \| categorical \| dicot \| NA \| NA \| 0.66 \| NA \| NA \| NA \| NA \| \| Phys \| ejercicio_fisico \| categorical \| dicot \| NA \| NA \| 1.00 \| NA \| NA \| NA \| NA \| \| Phys \| frec_ej_fisico \| categorical \| ordinal \| NA \| NA \| NA \| 0.94 \| NA \| NA \| NA \| \| Phys \| frec_ej_fisico_cardio \| categorical \| ordinal \| NA \| NA \| NA \| 0.88 \| NA \| NA \| NA \| \| Phys \| frec_ej_fisico_relaj \| categorical \| ordinal \| NA \| NA \| NA \| 1.00 \| NA \| NA \| NA \| \| Pollt \| cambio_aumento_trafico_veh \| categorical \| dicot \| NA \| NA \| NA \| NA \| NA \| NA \| NA \| \| Pollt \| cambio_donde_vives \| categorical \| dicot \| NA \| NA \| 1.00 \| NA \| NA \| NA \| NA \| \| Pollt \| cambio_mov_personas \| categorical \| dicot \| NA \| NA \| 1.00 \| NA \| NA \| NA \| NA \| \| Pollt \| cambio_nuevas_construcc \| categorical \| dicot \| NA \| NA \| 1.00 \| NA \| NA \| NA \| NA \| \| Pollt \| cambio_nuevas_fabricas \| categorical \| dicot \| NA \| NA \| NA \| NA \| NA \| NA \| NA \| \| Pollt \| contam_amb_desconst \| categorical \| dicot \| NA \| NA \| 0.79 \| NA \| NA \| NA \| NA \| \| Pollt \| contam_amb_dessol \| categorical \| dicot \| NA \| NA \| 1.00 \| NA \| NA \| NA \| NA \| \| Pollt \| contam_amb_mucho \| categorical \| dicot \| NA \| NA \| 1.00 \| NA \| NA \| NA \| NA \| \| Pollt \| contam_amb_traficvh \| categorical \| dicot \| NA \| NA \| 1.00 \| NA \| NA \| NA \| NA \| \| Pollt \| contam_ambiental \| categorical \| dicot \| NA \| NA \| 0.91 \| NA \| NA \| NA \| NA \| \| Pollt \| usa_quimicos_casa \| categorical \| dicot \| NA \| NA \| 0.89 \| NA \| NA \| NA \| NA \| \| Sednt \| cuanto_descanso \| categorical \| ordinal \| NA \| NA \| NA \| 1.00 \| NA \| NA \| NA \| \| Sednt \| horas_sentado \| categorical \| ordinal \| NA \| NA \| NA \| 0.95 \| NA \| NA \| NA \| \| Selfc \| aborda_veh_chof_alcohol \| categorical \| dicot \| NA \| NA \| -0.02 \| NA \| NA \| NA \| NA \| \| Selfc \| ac_estomat1va \| categorical \| dicot \| NA \| NA \| 0.97 \| NA \| NA \| NA \| NA \| \| Selfc \| ac_evita_sol \| categorical \| dicot \| NA \| NA \| 1.00 \| NA \| NA \| NA \| NA \| \| Selfc \| ac_med1va \| categorical \| dicot \| NA \| NA \| 1.00 \| NA \| NA \| NA \| NA \| \| Selfc \| ac_usa_prot_solar \| categorical \| dicot \| NA \| NA \| 0.81 \| NA \| NA \| NA \| NA \| \| Selfc \| cheq_glic_1va \| categorical \| dicot \| NA \| NA \| 0.90 \| NA \| NA \| NA \| NA \| \| Selfc \| cheq_mama_1va \| categorical \| dicot \| NA \| NA \| 0.93 \| NA \| NA \| NA \| NA \| \| Selfc \| cheq_obs_cuerpo \| categorical \| dicot \| NA \| NA \| 0.88 \| NA \| NA \| NA \| NA \| \| Selfc \| cheq_pr_citol_1va \| categorical \| dicot \| NA \| NA \| 0.66 \| NA \| NA \| NA \| NA \| \| Selfc \| cheq_prost_1va \| categorical \| dicot \| NA \| NA \| 0.91 \| NA \| NA \| NA \| NA \| \| Selfc \| cheq_ta_1va \| categorical \| dicot \| NA \| NA \| 1.00 \| NA \| NA \| NA \| NA \| \| Selfc \| cheq_tricol_1va \| categorical \| dicot \| NA \| NA \| 0.97 \| NA \| NA \| NA \| NA \| \| Selfc \| obed_ley_trans \| categorical \| dicot \| NA \| NA \| 0.78 \| NA \| NA \| NA \| NA \| \| Selfc \| prot_solar_crema \| categorical \| dicot \| NA \| NA \| 0.79 \| NA \| NA \| NA \| NA \| \| Selfc \| prot_solar_gafas \| categorical \| dicot \| NA \| NA \| 0.92 \| NA \| NA \| NA \| NA \| \| Selfc \| prot_solar_gorra \| categorical \| dicot \| NA \| NA \| 0.80 \| NA \| NA \| NA \| NA \| \| Selfc \| prot_solar_ropas \| categorical \| dicot \| NA \| NA \| 0.75 \| NA \| NA \| NA \| NA \| \| Selfc \| prot_solar_sombrero \| categorical \| dicot \| NA \| NA \| 1.00 \| NA \| NA \| NA \| NA \| \| Selfc \| usa_cint_seguridad \| categorical \| dicot \| NA \| NA \| 0.74 \| NA \| NA \| NA \| NA \| \| Sleep \| despierta_multiplesv \| categorical \| dicot \| NA \| NA \| 0.66 \| NA \| NA \| NA \| NA \| \| Sleep \| dificul_dormir \| categorical \| dicot \| NA \| NA \| 0.83 \| NA \| NA \| NA \| NA \| \| Sleep \| levanta_descansado \| categorical \| dicot \| NA \| NA \| 0.74 \| NA \| NA \| NA \| NA \| \| Sleep \| prom_horas_sueno_dia \| categorical \| ordinal \| NA \| NA \| NA \| 1.00 \| NA \| NA \| NA \| \| Sleep \| sueno_durante_dia \| categorical \| dicot \| NA \| NA \| 0.85 \| NA \| NA \| NA \| NA \| \| Sleep \| usa_pastillas_dormir \| categorical \| dicot \| NA \| NA \| 0.84 \| NA \| NA \| NA \| NA \| \| StrsV \| amb_trabajo \| categorical \| dicot \| NA \| NA \| 1.00 \| NA \| NA \| NA \| NA \| \| StrsV \| clasif_trabajo \| categorical \| ordinal \| NA \| NA \| NA \| 0.97 \| NA \| NA \| NA \| \| StrsV \| disfruta_trabajo \| categorical \| ordinal \| NA \| NA \| NA \| 1.00 \| NA \| NA \| NA \| \| StrsV \| stress_casa \| categorical \| dicot \| NA \| NA \| 0.83 \| NA \| NA \| NA \| NA \| \| StrsV \| stress_casa_padres \| categorical \| dicot \| NA \| NA \| 1.00 \| NA \| NA \| NA \| NA \| \| StrsV \| violenc_entorno \| categorical \| dicot \| NA \| NA \| 1.00 \| NA \| NA \| NA \| NA \| \| StrsV \| violenc_verb_fis_xpadrs \| categorical \| dicot \| NA \| NA \| 1.00 \| NA \| NA \| NA \| NA \| \| WrkEn \| act_fisica_trabajo \| categorical \| ordinal \| NA \| NA \| NA \| 1.00 \| NA \| NA \| NA \| \| WrkEn \| antec_accid_laboral \| categorical \| dicot \| NA \| NA \| NA \| NA \| NA \| NA \| NA \| \| WrkEn \| descanso_en_trabajo \| categorical \| dicot \| NA \| NA \| 1.00 \| NA \| NA \| NA \| NA \| \| WrkEn \| horas_trabajo \| categorical \| ordinal \| NA \| NA \| NA \| 0.94 \| NA \| NA \| NA \| \| WrkEn \| medio_protec \| categorical \| dicot \| NA \| NA \| 0.48 \| NA \| NA \| NA \| NA \| \| WrkEn \| ubicac_lug_trabajo \| categorical \| politom \| NA \| NA \| 0.89 \| NA \| NA \| NA \| NA \| \| ^a^ Demog - Demographic, Diet - negative nutritional habits, Phys - Physical activity, FreeT - healthy free time activities, Selfc - self care, CsMed - Excessive consumption of medicines, CsNat - consumption of natural products, CPsych - consumption of psychoactive products, EconR - economic restrictions, StrsV - exposure to stress and violence, Sleep - sleep quality, WrkEn - work environment risks, Pollt - pollution, Sednt - sedentary behaviour, CCI - comorbidities, General - other convenience variables, Overall - all included variables. \| \| \| \| \| \| \| \| \| \| \| \| ^b^ Shapiro-Wilk test of normality statistic \| \| \| \| \| \| \| \| \| \| \| \| ^c^ p-value for the Shapiro-Wilk test of normality \| \| \| \| \| \| \| \| \| \| \| \| ^d^ Cohen’s kappa coefficients were calculated for categorical variables \| \| \| \| \| \| \| \| \| \| \| \| ^e^ Fleiss’s kappa coefficients were calculated for ordinal variables \| \| \| \| \| \| \| \| \| \| \| \| ^f^ Pearson’s product-moment correlation coefficients (R) were calculated for numeric variables with normal distribution. \| \| \| \| \| \| \| \| \| \| \| \| ^g^ Spearman’s rank correlation (rho) were calculated for numeric variables with non-normal distribution. Shapiro test was used for assessing normality. \| \| \| \| \| \| \| \| \| \| \| \| ^h^ ICC-Intraclass correlation coefficient were calculated for numeric variables \| \| \| \| \| \| \| \| \| \| \| |
| --- | --- | --- | --- | --- | --- | --- | --- | --- | --- | --- | --- | --- | --- | --- | --- | --- | --- | --- | --- | --- | --- | --- | --- | --- | --- | --- | --- | --- | --- | --- | --- | --- | --- | --- | --- | --- | --- | --- | --- | --- | --- | --- | --- | --- | --- | --- | --- | --- | --- | --- | --- | --- | --- | --- | --- | --- | --- | --- | --- | --- | --- | --- | --- | --- | --- | --- | --- | --- | --- | --- | --- | --- | --- | --- | --- | --- | --- | --- | --- | --- | --- | --- | --- | --- | --- | --- | --- | --- | --- | --- | --- | --- | --- | --- | --- | --- | --- | --- | --- | --- | --- | --- | --- | --- | --- | --- | --- | --- | --- | --- | --- | --- | --- | --- | --- | --- | --- | --- | --- | --- | --- | --- | --- | --- | --- | --- | --- | --- | --- | --- | --- | --- | --- | --- | --- | --- | --- | --- | --- | --- | --- | --- | --- | --- | --- | --- | --- | --- | --- | --- | --- | --- | --- | --- | --- | --- | --- | --- | --- | --- | --- | --- | --- | --- | --- | --- | --- | --- | --- | --- | --- | --- | --- | --- | --- | --- | --- | --- | --- | --- | --- | --- | --- | --- | --- | --- | --- | --- | --- | --- | --- | --- | --- | --- | --- | --- | --- | --- | --- | --- | --- | --- | --- | --- | --- | --- | --- | --- | --- | --- | --- | --- | --- | --- | --- | --- | --- | --- | --- | --- | --- | --- | --- | --- | --- | --- | --- | --- | --- | --- | --- | --- | --- | --- | --- | --- | --- | --- | --- | --- | --- | --- | --- | --- | --- | --- | --- | --- | --- | --- | --- | --- | --- | --- | --- | --- | --- | --- | --- | --- | --- | --- | --- | --- | --- | --- | --- | --- | --- | --- | --- | --- | --- | --- | --- | --- | --- | --- | --- | --- | --- | --- | --- | --- | --- | --- | --- | --- | --- | --- | --- | --- | --- | --- | --- | --- | --- | --- | --- | --- | --- | --- | --- | --- | --- | --- | --- | --- | --- | --- | --- | --- | --- | --- | --- | --- | --- | --- | --- | --- | --- | --- | --- | --- | --- | --- | --- | --- | --- | --- | --- | --- | --- | --- | --- | --- | --- | --- | --- | --- | --- | --- | --- | --- | --- | --- | --- | --- | --- | --- | --- | --- | --- | --- | --- | --- | --- | --- | --- | --- | --- | --- | --- | --- | --- | --- | --- | --- | --- | --- | --- | --- | --- | --- | --- | --- | --- | --- | --- | --- | --- | --- | --- | --- | --- | --- | --- | --- | --- | --- | --- | --- | --- | --- | --- | --- | --- | --- | --- | --- | --- | --- | --- | --- | --- | --- | --- | --- | --- | --- | --- | --- | --- | --- | --- | --- | --- | --- | --- | --- | --- | --- | --- | --- | --- | --- | --- | --- | --- | --- | --- | --- | --- | --- | --- | --- | --- | --- | --- | --- | --- | --- | --- | --- | --- | --- | --- | --- | --- | --- | --- | --- | --- | --- | --- | --- | --- | --- | --- | --- | --- | --- | --- | --- | --- | --- | --- | --- | --- | --- | --- | --- | --- | --- | --- | --- | --- | --- | --- | --- | --- | --- | --- | --- | --- | --- | --- | --- | --- | --- | --- | --- | --- | --- | --- | --- | --- | --- | --- | --- | --- | --- | --- | --- | --- | --- | --- | --- | --- | --- | --- | --- | --- | --- | --- | --- | --- | --- | --- | --- | --- | --- | --- | --- | --- | --- | --- | --- | --- | --- | --- | --- | --- | --- | --- | --- | --- | --- | --- | --- | --- | --- | --- | --- | --- | --- | --- | --- | --- | --- | --- | --- | --- | --- | --- | --- | --- | --- | --- | --- | --- | --- | --- | --- | --- | --- | --- | --- | --- | --- | --- | --- | --- | --- | --- | --- | --- | --- | --- | --- | --- | --- | --- | --- | --- | --- | --- | --- | --- | --- | --- | --- | --- | --- | --- | --- | --- | --- | --- | --- | --- | --- | --- | --- | --- | --- | --- | --- | --- | --- | --- | --- | --- | --- | --- | --- | --- | --- | --- | --- | --- | --- | --- | --- | --- | --- | --- | --- | --- | --- | --- | --- | --- | --- | --- | --- | --- | --- | --- | --- | --- | --- | --- | --- | --- | --- | --- | --- | --- | --- | --- | --- | --- | --- | --- | --- | --- | --- | --- | --- | --- | --- | --- | --- | --- | --- | --- | --- | --- | --- | --- | --- | --- | --- | --- | --- | --- | --- | --- | --- | --- | --- | --- | --- | --- | --- | --- | --- | --- | --- | --- | --- | --- | --- | --- | --- | --- | --- | --- | --- | --- | --- | --- | --- | --- | --- | --- | --- | --- | --- | --- | --- | --- | --- | --- | --- | --- | --- | --- | --- | --- | --- | --- | --- | --- | --- | --- | --- | --- | --- | --- | --- | --- | --- | --- | --- | --- | --- | --- | --- | --- | --- | --- | --- | --- | --- | --- | --- | --- | --- | --- | --- | --- | --- | --- | --- | --- | --- | --- | --- | --- | --- | --- | --- | --- | --- | --- | --- | --- | --- | --- | --- | --- | --- | --- | --- | --- | --- | --- | --- | --- | --- | --- | --- | --- | --- | --- | --- | --- | --- | --- | --- | --- | --- | --- | --- | --- | --- | --- | --- | --- | --- | --- | --- | --- | --- | --- | --- | --- | --- | --- | --- | --- | --- | --- | --- | --- | --- | --- | --- | --- | --- | --- | --- | --- | --- | --- | --- | --- | --- | --- | --- | --- | --- | --- | --- | --- | --- | --- | --- | --- | --- | --- | --- | --- | --- | --- | --- | --- | --- | --- | --- | --- | --- | --- | --- | --- | --- | --- | --- | --- | --- | --- | --- | --- | --- | --- | --- | --- | --- | --- | --- | --- | --- | --- | --- | --- | --- | --- | --- | --- | --- | --- | --- | --- | --- | --- | --- | --- | --- | --- | --- | --- | --- | --- | --- | --- | --- | --- | --- | --- | --- | --- | --- | --- | --- | --- | --- | --- | --- | --- | --- | --- | --- | --- | --- | --- | --- | --- | --- | --- | --- | --- | --- | --- | --- | --- | --- | --- | --- | --- | --- | --- | --- | --- | --- | --- | --- | --- | --- | --- | --- | --- | --- | --- | --- | --- | --- | --- | --- | --- | --- | --- | --- | --- | --- | --- | --- | --- | --- | --- | --- | --- | --- | --- | --- | --- | --- | --- | --- | --- | --- | --- | --- | --- | --- | --- | --- | --- | --- | --- | --- | --- | --- | --- | --- | --- | --- | --- | --- | --- | --- | --- | --- | --- | --- | --- | --- | --- | --- | --- | --- | --- | --- | --- | --- | --- | --- | --- | --- | --- | --- | --- | --- | --- | --- | --- | --- | --- | --- | --- | --- | --- | --- | --- | --- | --- | --- | --- | --- | --- | --- | --- | --- | --- | --- | --- | --- | --- | --- | --- | --- | --- | --- | --- | --- | --- | --- | --- | --- | --- | --- | --- | --- | --- | --- | --- | --- | --- | --- | --- | --- | --- | --- | --- | --- | --- | --- | --- | --- | --- | --- | --- | --- | --- | --- | --- | --- | --- | --- | --- | --- | --- | --- | --- | --- | --- | --- | --- | --- | --- | --- | --- | --- | --- | --- | --- | --- | --- | --- | --- | --- | --- | --- | --- | --- | --- | --- | --- | --- | --- | --- | --- | --- | --- | --- | --- | --- | --- | --- | --- | --- | --- | --- | --- | --- | --- | --- | --- | --- | --- | --- | --- | --- | --- | --- | --- | --- | --- | --- | --- | --- | --- | --- | --- | --- | --- | --- | --- | --- | --- | --- | --- | --- | --- | --- | --- | --- | --- | --- | --- | --- | --- | --- | --- | --- | --- | --- | --- | --- | --- | --- | --- | --- | --- | --- | --- | --- | --- | --- | --- | --- | --- | --- | --- | --- | --- | --- | --- | --- | --- | --- | --- | --- | --- | --- | --- | --- | --- | --- | --- | --- | --- | --- | --- | --- | --- | --- | --- | --- | --- | --- | --- | --- | --- | --- | --- | --- | --- | --- | --- | --- | --- | --- | --- | --- | --- | --- | --- | --- | --- | --- | --- | --- | --- | --- | --- | --- | --- | --- | --- | --- | --- | --- | --- | --- | --- | --- | --- | --- | --- | --- | --- | --- | --- | --- | --- | --- | --- | --- | --- | --- | --- | --- | --- | --- | --- | --- | --- | --- | --- | --- | --- | --- | --- | --- | --- | --- | --- | --- | --- | --- | --- | --- | --- | --- | --- | --- | --- | --- | --- | --- | --- | --- | --- | --- | --- | --- | --- | --- | --- | --- | --- | --- | --- | --- | --- | --- | --- | --- | --- | --- | --- | --- | --- | --- | --- | --- | --- | --- | --- | --- | --- | --- | --- | --- | --- | --- | --- | --- | --- | --- | --- | --- | --- | --- | --- | --- | --- | --- | --- | --- | --- | --- | --- | --- | --- | --- | --- | --- | --- | --- | --- | --- | --- | --- | --- | --- | --- | --- | --- | --- | --- | --- | --- | --- | --- | --- | --- | --- | --- | --- | --- | --- | --- | --- | --- | --- | --- | --- | --- | --- | --- | --- | --- | --- | --- | --- | --- | --- | --- | --- | --- | --- | --- | --- | --- | --- | --- | --- | --- | --- | --- | --- | --- | --- | --- | --- | --- | --- | --- | --- | --- | --- | --- | --- | --- | --- | --- | --- | --- | --- | --- | --- | --- | --- | --- | --- | --- | --- | --- | --- | --- | --- | --- | --- | --- | --- | --- | --- | --- | --- | --- | --- | --- | --- | --- | --- | --- | --- | --- | --- | --- | --- | --- | --- | --- | --- | --- | --- | --- | --- | --- | --- | --- | --- | --- | --- | --- | --- | --- | --- | --- | --- | --- | --- | --- | --- | --- | --- | --- | --- | --- | --- | --- | --- | --- | --- | --- | --- | --- | --- | --- | --- | --- | --- | --- | --- | --- | --- | --- | --- | --- | --- | --- | --- | --- | --- | --- | --- | --- | --- | --- | --- | --- | --- | --- | --- | --- | --- | --- | --- | --- | --- | --- | --- | --- | --- | --- | --- | --- | --- | --- | --- | --- | --- | --- | --- | --- | --- | --- | --- | --- | --- | --- | --- | --- | --- | --- | --- | --- | --- | --- | --- | --- | --- | --- | --- | --- | --- | --- | --- | --- | --- | --- | --- | --- | --- | --- | --- | --- | --- | --- | --- | --- | --- | --- | --- | --- | --- | --- | --- | --- | --- | --- | --- | --- | --- | --- | --- | --- | --- | --- | --- | --- | --- | --- | --- | --- | --- | --- | --- | --- | --- | --- | --- | --- | --- | --- | --- | --- | --- | --- | --- | --- | --- | --- | --- | --- | --- | --- | --- | --- | --- | --- | --- | --- | --- | --- | --- | --- | --- | --- | --- | --- | --- | --- | --- | --- | --- | --- | --- | --- | --- | --- | --- | --- | --- | --- | --- | --- | --- | --- | --- | --- | --- | --- | --- | --- | --- | --- | --- | --- | --- | --- | --- | --- | --- | --- | --- | --- | --- | --- | --- | --- | --- | --- | --- | --- | --- | --- | --- | --- | --- | --- | --- | --- | --- | --- | --- | --- | --- | --- | --- | --- | --- | --- | --- | --- | --- | --- | --- | --- | --- | --- | --- | --- | --- | --- | --- | --- | --- | --- | --- | --- | --- | --- | --- | --- | --- | --- | --- | --- | --- | --- | --- | --- | --- | --- | --- | --- | --- | --- | --- | --- | --- | --- | --- | --- | --- | --- | --- | --- | --- | --- | --- | --- | --- | --- | --- | --- | --- | --- | --- | --- | --- | --- | --- | --- | --- | --- | --- | --- | --- | --- | --- | --- | --- | --- | --- | --- | --- | --- | --- | --- | --- | --- | --- | --- | --- | --- | --- | --- | --- | --- | --- | --- | --- | --- | --- | --- | --- | --- | --- | --- | --- | --- | --- | --- | --- | --- | --- | --- | --- | --- | --- | --- | --- | --- | --- | --- | --- | --- | --- | --- | --- | --- | --- | --- | --- | --- | --- | --- | --- | --- | --- | --- | --- | --- | --- | --- | --- | --- | --- | --- | --- | --- | --- | --- | --- | --- | --- | --- | --- | --- | --- | --- | --- | --- | --- | --- | --- | --- | --- | --- | --- | --- | --- | --- | --- | --- | --- | --- | --- | --- | --- | --- | --- | --- | --- | --- | --- | --- | --- | --- | --- | --- | --- | --- | --- | --- | --- | --- | --- | --- | --- | --- | --- | --- | --- | --- | --- | --- | --- | --- | --- | --- | --- | --- | --- | --- | --- | --- | --- | --- | --- | --- | --- | --- | --- | --- | --- | --- | --- | --- | --- | --- | --- | --- | --- | --- | --- | --- | --- | --- | --- | --- | --- | --- | --- | --- | --- | --- | --- | --- | --- | --- | --- | --- | --- | --- | --- | --- | --- | --- | --- | --- | --- | --- | --- | --- | --- | --- | --- | --- | --- | --- | --- | --- | --- | --- | --- | --- | --- | --- | --- | --- | --- | --- | --- | --- | --- | --- | --- | --- | --- | --- | --- | --- | --- | --- | --- | --- | --- | --- | --- | --- | --- | --- | --- | --- | --- | --- | --- | --- | --- | --- | --- | --- | --- | --- | --- | --- | --- | --- | --- | --- | --- | --- | --- | --- | --- | --- | --- | --- | --- | --- | --- | --- | --- | --- | --- | --- | --- | --- | --- | --- | --- | --- | --- | --- | --- | --- | --- | --- | --- | --- | --- | --- | --- | --- | --- | --- | --- | --- | --- | --- | --- | --- | --- | --- | --- | --- | --- | --- | --- | --- | --- | --- | --- | --- | --- | --- | --- | --- | --- | --- | --- | --- | --- | --- | --- | --- | --- | --- | --- | --- | --- | --- | --- | --- | --- | --- | --- | --- | --- | --- | --- | --- | --- | --- | --- | --- | --- | --- | --- | --- | --- | --- | --- | --- | --- | --- | --- | --- | --- | --- | --- | --- | --- | --- | --- | --- | --- | --- | --- | --- | --- | --- | --- | --- | --- | --- | --- | --- | --- | --- | --- | --- | --- | --- | --- | --- | --- | --- | --- | --- | --- | --- | --- | --- | --- | --- | --- | --- | --- | --- | --- | --- | --- | --- | --- | --- | --- | --- | --- | --- | --- | --- | --- | --- | --- | --- | --- | --- | --- | --- | --- | --- | --- | --- | --- | --- | --- | --- | --- | --- | --- | --- | --- | --- | --- | --- | --- | --- | --- | --- | --- | --- | --- | --- | --- | --- | --- | --- | --- | --- | --- | --- | --- | --- | --- | --- | --- | --- | --- | --- | --- | --- | --- | --- | --- | --- | --- | --- | --- | --- | --- | --- | --- | --- | --- | --- | --- | --- | --- | --- | --- | --- | --- | --- | --- | --- | --- | --- | --- | --- | --- | --- | --- | --- | --- | --- | --- | --- | --- | --- | --- | --- | --- | --- | --- | --- | --- | --- | --- | --- | --- | --- | --- | --- | --- | --- | --- | --- | --- | --- | --- | --- | --- | --- | --- | --- | --- | --- | --- | --- | --- | --- | --- | --- | --- | --- | --- | --- | --- | --- | --- | --- | --- | --- | --- | --- | --- | --- | --- | --- | --- | --- | --- | --- | --- | --- | --- | --- | --- | --- | --- | --- | --- | --- | --- | --- | --- | --- | --- | --- | --- | --- | --- | --- | --- | --- | --- | --- | --- | --- | --- | --- | --- | --- | --- | --- | --- | --- | --- | --- | --- | --- | --- | --- | --- | --- | --- | --- | --- | --- | --- | --- | --- | --- | --- | --- | --- | --- | --- | --- | --- | --- | --- | --- | --- | --- | --- | --- | --- | --- | --- | --- | --- | --- | --- | --- | --- | --- | --- | --- | --- | --- | --- | --- | --- | --- | --- | --- | --- | --- | --- | --- | --- | --- | --- | --- | --- | --- | --- | --- | --- | --- | --- | --- | --- | --- | --- | --- | --- | --- | --- | --- | --- | --- | --- | --- | --- | --- | --- | --- | --- | --- | --- | --- | --- | --- | --- | --- | --- | --- | --- | --- | --- | --- | --- | --- | --- | --- | --- | --- | --- | --- | --- | --- | --- | --- | --- | --- | --- | --- | --- | --- | --- | --- | --- | --- | --- | --- | --- | --- | --- | --- | --- | --- | --- | --- | --- | --- | --- | --- | --- | --- | --- | --- | --- | --- | --- | --- | --- | --- | --- | --- | --- | --- | --- | --- | --- | --- | --- | --- | --- | --- | --- | --- | --- | --- | --- | --- | --- | --- | --- | --- | --- | --- | --- | --- | --- | --- | --- | --- | --- | --- | --- | --- | --- | --- | --- | --- | --- | --- | --- | --- | --- | --- | --- | --- | --- | --- | --- | --- | --- | --- | --- | --- | --- | --- | --- | --- | --- | --- | --- | --- | --- | --- | --- | --- | --- | --- | --- | --- | --- | --- | --- | --- | --- | --- | --- | --- | --- | --- | --- | --- | --- | --- | --- | --- | --- | --- | --- | --- | --- | --- | --- | --- | --- | --- | --- | --- | --- | --- | --- | --- | --- | --- | --- | --- | --- | --- | --- | --- | --- | --- | --- | --- | --- | --- | --- | --- | --- | --- | --- | --- | --- | --- | --- | --- | --- | --- | --- | --- | --- | --- | --- | --- | --- | --- | --- | --- | --- | --- | --- | --- | --- | --- | --- | --- | --- | --- | --- | --- | --- | --- | --- | --- | --- | --- | --- | --- | --- | --- | --- | --- | --- | --- | --- | --- | --- | --- | --- | --- | --- | --- | --- | --- | --- | --- | --- | --- | --- | --- | --- | --- | --- | --- | --- | --- | --- | --- | --- | --- | --- | --- | --- | --- | --- | --- | --- | --- | --- | --- | --- | --- | --- | --- | --- | --- | --- | --- | --- | --- | --- | --- | --- | --- | --- | --- | --- | --- | --- | --- | --- | --- | --- | --- | --- | --- | --- | --- | --- | --- | --- | --- | --- | --- | --- | --- | --- | --- | --- | --- | --- | --- | --- | --- | --- | --- | --- | --- | --- | --- | --- | --- | --- | --- | --- | --- | --- | --- | --- | --- | --- | --- | --- | --- | --- | --- | --- | --- | --- | --- | --- | --- | --- | --- | --- | --- | --- | --- | --- | --- | --- | --- | --- | --- | --- | --- | --- | --- | --- | --- | --- | --- | --- | --- | --- | --- | --- | --- | --- | --- | --- | --- | --- | --- | --- | --- |
