# Supplementary material for: Development and validation of a questionnaire to assess environmental and lifestyle factors influencing infectious disease outcomes: application to SARS-CoV-2-infected individuals in Cuba
Source: Front Public Health. 2026 Jun 29;14:1805212. doi: 10.3389/fpubh.2026.1805212 (PMC13357406; doi:10.3389/fpubh.2026.1805212)
Supplement: Supplementary file 5 [file Table_5.docx]

Supplementary Material 4: Confirmatory factor analysis

## Separate CFA for each dimension

| Table S4.1: Kaiser-Meyer-Olkin (KMO) test of factorial adequacy and Bartlett’s test. MSA: Measure of sampling adequacy (Kaiser,1970); df: degrees of freedom.   \| dimension \| items \| KMO (MSA) \| Bartlett's test \| \| \| \| --- \| --- \| --- \| --- \| --- \| --- \| \| $\chi^{2}$ \| df \| p-value \| \| Diet \| 33 \| 0.652 \| 2448.212 \| 528 \| 0.000 \| \| Physical \| 5 \| 0.742 \| 316.664 \| 10 \| 0.000 \| \| Sedentary \| 2 \| 0.500 \| 0.047 \| 1 \| 0.828 \| \| Freetime \| 12 \| 0.668 \| 572.130 \| 66 \| 0.000 \| \| Selfcare \| 12 \| 0.808 \| 1446.925 \| 66 \| 0.000 \| \| ConsMeds \| 3 \| 0.543 \| 40.904 \| 3 \| 0.000 \| \| ConsNat \| 4 \| 0.460 \| 700.989 \| 6 \| 0.000 \| \| ConsPsych \| 7 \| 0.534 \| 222.157 \| 21 \| 0.000 \| \| EconDepr \| 20 \| 0.715 \| 2000.832 \| 190 \| 0.000 \| \| Stress \| 7 \| 0.599 \| 372.615 \| 21 \| 0.000 \| \| Sleep \| 6 \| 0.758 \| 355.471 \| 15 \| 0.000 \| \| Workenv \| 6 \| 0.576 \| 309.125 \| 15 \| 0.000 \| \| Pollut \| 12 \| 0.480 \| 1212.406 \| 66 \| 0.000 \| |
| --- | --- | --- | --- | --- | --- | --- | --- | --- | --- | --- | --- | --- | --- | --- | --- | --- | --- | --- | --- | --- | --- | --- | --- | --- | --- | --- | --- | --- | --- | --- | --- | --- | --- | --- | --- | --- | --- | --- | --- | --- | --- | --- | --- | --- | --- | --- | --- | --- | --- | --- | --- | --- | --- | --- | --- | --- | --- | --- | --- | --- | --- | --- | --- | --- | --- | --- | --- | --- | --- | --- | --- | --- | --- | --- | --- | --- | --- | --- | --- | --- | --- | --- | --- | --- | --- | --- | --- |

| Table S4.2: Fit measures ($\chi^{2}$, TFI, CFI, TLI, RMSEA) by CFA model.   \| model \| $\chi^{2}$ \| df \| p-value \| CFI \| TLI \| RMSEA \| SRMR \| \| --- \| --- \| --- \| --- \| --- \| --- \| --- \| --- \| \| Diet \| 2520.6 (1656.7) \| 495 (495) \| NA (0.000) \| 0.810 (0.746) \| 0.797 (0.729) \| 0.115 (0.087) \| 0.208 \| \| Physical \| 9.1 (13) \| 5 (5) \| NA (0.024) \| 0.997 (0.991) \| 0.993 (0.983) \| 0.051 (0.072) \| 0.112 \| \| Seden-tary \| 0.0 (0) \| 0 (0) \| NA ( NA) \| 1.000 (1.000) \| 1.000 (1.000) \| 0.000 (0.000) \| 0.000 \| \| Cons-Med \| 0.0 (0) \| 0 (0) \| NA ( NA) \| 1.000 (1.000) \| 1.000 (1.000) \| 0.000 (0.000) \| 0.000 \| \| ConsNat \| 2.9 (3.6) \| 2 (2) \| NA (0.168) \| 1.000 (1.000) \| 1.000 (1.000) \| 0.038 (0.050) \| 0.066 \| \| Cons-Psych \| 7.4 (8.7) \| 9 (9) \| NA (0.463) \| 1.000 (1.000) \| 1.011 (1.003) \| 0.000 (0.000) \| 0.220 \| \| Econ-Depr \| 1091.9 (628) \| 170 (170) \| NA (0.000) \| 0.904 (0.874) \| 0.892 (0.859) \| 0.133 (0.094) \| 0.262 \| \| Freetime \| 171.1 (180.6) \| 54 (54) \| NA (0.000) \| 0.874 (0.796) \| 0.846 (0.750) \| 0.090 (0.093) \| 0.162 \| \| Pollut \| 217.9 (161.5) \| 54 (54) \| NA (0.000) \| 0.921 (0.897) \| 0.903 (0.874) \| 0.099 (0.080) \| 0.224 \| \| Selfcare \| 171.8 (202.5) \| 54 (54) \| NA (0.000) \| 0.992 (0.982) \| 0.990 (0.978) \| 0.084 (0.095) \| 0.152 \| \| Sleep \| 12.7 (18.8) \| 9 (9) \| NA (0.027) \| 0.996 (0.986) \| 0.993 (0.977) \| 0.037 (0.059) \| 0.076 \| \| Stress \| 34.7 (34.3) \| 14 (14) \| NA (0.002) \| 0.973 (0.964) \| 0.959 (0.946) \| 0.075 (0.074) \| 0.171 \| \| Workenv \| 23.8 (23.4) \| 9 (9) \| NA (0.005) \| 0.954 (0.941) \| 0.923 (0.902) \| 0.073 (0.072) \| 0.160 \| |
| --- | --- | --- | --- | --- | --- | --- | --- | --- | --- | --- | --- | --- | --- | --- | --- | --- | --- | --- | --- | --- | --- | --- | --- | --- | --- | --- | --- | --- | --- | --- | --- | --- | --- | --- | --- | --- | --- | --- | --- | --- | --- | --- | --- | --- | --- | --- | --- | --- | --- | --- | --- | --- | --- | --- | --- | --- | --- | --- | --- | --- | --- | --- | --- | --- | --- | --- | --- | --- | --- | --- | --- | --- | --- | --- | --- | --- | --- | --- | --- | --- | --- | --- | --- | --- | --- | --- | --- | --- | --- | --- | --- | --- | --- | --- | --- | --- | --- | --- | --- | --- | --- | --- | --- | --- | --- | --- | --- | --- | --- | --- | --- | --- |

Notes: CFA estimation method: Diagonally Weighted Least Square (DWLS) with delta parameterization. CFI = Comparative fit index, TLI = Tucker-Lewis index, RMSEA = Root Mean Square Error of Approximation. Values within parentheses are scaled statistics.

| Table S4.3: Average variance extracted by CFA model.   \| dimension \| AVE \| \| --- \| --- \| \| Diet \| 0.23 \| \| Physical \| 0.71 \| \| Sedentary \| 0.63 \| \| Freetime \| 0.36 \| \| Selfcare \| 0.46 \| \| ConsMeds \| 0.42 \| \| ConsNat \| 0.66 \| \| ConsPsych \| 0.31 \| \| EconDepr \| 0.39 \| \| Stress \| 0.43 \| \| Sleep \| 0.53 \| \| Workenv \| 0.42 \| \| Pollut \| 0.51 \| |
| --- | --- | --- | --- | --- | --- | --- | --- | --- | --- | --- | --- | --- | --- | --- | --- | --- | --- | --- | --- | --- | --- | --- | --- | --- | --- | --- | --- | --- |

| Table S4.4: Standardized loadings by model.   \| factor \| indicator \| B \| SE \| Z \| p-value \| Beta \| \| --- \| --- \| --- \| --- \| --- \| --- \| --- \| \| Diet \| comid_horario_regul \| 1.000 \| 0.000 \| NA \| NA \| 0.502 \| \| Diet \| desayuna_antes \| 1.846 \| 0.199 \| 9.265 \| 0.000 \| 0.926 \| \| Diet \| desayuno \| 1.988 \| 0.218 \| 9.109 \| 0.000 \| 0.998 \| \| Diet \| almuerzo \| 0.807 \| 0.209 \| 3.852 \| 0.000 \| 0.405 \| \| Diet \| cena \| 1.099 \| 0.241 \| 4.555 \| 0.000 \| 0.551 \| \| Diet \| merienda_manana \| 1.129 \| 0.132 \| 8.584 \| 0.000 \| 0.567 \| \| Diet \| merienda_tarde \| 1.304 \| 0.150 \| 8.696 \| 0.000 \| 0.654 \| \| Diet \| merienda_desp_cena \| 1.194 \| 0.143 \| 8.321 \| 0.000 \| 0.599 \| \| Diet \| toma_cafe \| -1.593 \| 0.178 \| -8.967 \| 0.000 \| -0.800 \| \| Diet \| cafe_con_azucar \| -1.593 \| 0.184 \| -8.658 \| 0.000 \| -0.799 \| \| Diet \| condim_sal \| -1.290 \| 0.168 \| -7.676 \| 0.000 \| -0.647 \| \| Diet \| dieta_p_perd_peso \| -0.291 \| 0.160 \| -1.827 \| 0.068 \| -0.146 \| \| Diet \| cantid_agua_toma_dia_menos4 \| 0.310 \| 0.165 \| 1.883 \| 0.060 \| 0.156 \| \| Diet \| com_rapida_m3vps \| -0.089 \| 0.131 \| -0.680 \| 0.496 \| -0.045 \| \| Diet \| mucha_sal \| 0.041 \| 0.140 \| 0.290 \| 0.772 \| 0.020 \| \| Diet \| ahumados_m1vps \| 0.341 \| 0.148 \| 2.306 \| 0.021 \| 0.171 \| \| Diet \| cons_refr_enlat \| 0.067 \| 0.136 \| 0.494 \| 0.621 \| 0.034 \| \| Diet \| cons_dulces_helados \| -0.285 \| 0.139 \| -2.055 \| 0.040 \| -0.143 \| \| Diet \| alim_dulces \| -0.221 \| 0.146 \| -1.510 \| 0.131 \| -0.111 \| \| Diet \| tipo_carne_res \| -0.938 \| 0.148 \| -6.354 \| 0.000 \| -0.471 \| \| Diet \| tipo_carne_carnero \| -0.599 \| 0.251 \| -2.389 \| 0.017 \| -0.301 \| \| Diet \| tipo_carne_cerdo \| -0.260 \| 0.146 \| -1.780 \| 0.075 \| -0.130 \| \| Diet \| tipo_carne_pollo \| -0.449 \| 0.300 \| -1.495 \| 0.135 \| -0.225 \| \| Diet \| tipo_carne_pescado \| 0.060 \| 0.135 \| 0.448 \| 0.654 \| 0.030 \| \| Diet \| x1vs_lacteos \| 0.711 \| 0.153 \| 4.646 \| 0.000 \| 0.357 \| \| Diet \| x1vs_vegetales \| -0.885 \| 0.166 \| -5.329 \| 0.000 \| -0.444 \| \| Diet \| x1vs_frutas \| -1.050 \| 0.149 \| -7.047 \| 0.000 \| -0.527 \| \| Diet \| x1vs_granos \| -1.187 \| 0.152 \| -7.793 \| 0.000 \| -0.596 \| \| Diet \| x1vs_panes \| -0.323 \| 0.218 \| -1.483 \| 0.138 \| -0.162 \| \| Diet \| x1vs_cereales \| -0.752 \| 0.135 \| -5.578 \| 0.000 \| -0.377 \| \| Diet \| condim_naturales \| -1.329 \| 0.176 \| -7.547 \| 0.000 \| -0.667 \| \| Diet \| condim_deshidratado \| -0.186 \| 0.141 \| -1.319 \| 0.187 \| -0.093 \| \| Diet \| condim_procesados \| 0.052 \| 0.137 \| 0.382 \| 0.702 \| 0.026 \| \| phys_act \| frec_ej_fisico \| 1.000 \| 0.000 \| NA \| NA \| 0.928 \| \| phys_act \| frec_ej_fisico_cardio \| 1.026 \| 0.068 \| 15.065 \| 0.000 \| 0.952 \| \| phys_act \| frec_ej_fisico_relaj \| 0.906 \| 0.080 \| 11.278 \| 0.000 \| 0.840 \| \| phys_act \| ej_fis_supervis_entrenador \| 0.943 \| 0.066 \| 14.239 \| 0.000 \| 0.875 \| \| phys_act \| ej_fis_supervis_fisiot \| 0.580 \| 0.127 \| 4.551 \| 0.000 \| 0.538 \| \| sedentary \| horas_sentado \| 1.000 \| 0.000 \| NA \| NA \| 0.794 \| \| sedentary \| cuanto_descanso \| 1.000 \| 0.000 \| NA \| NA \| 0.794 \| \| ConsMed \| toma_medicam \| 1.000 \| 0.000 \| NA \| NA \| 0.728 \| \| ConsMed \| muchos_medicam \| 1.097 \| 0.609 \| 1.800 \| 0.072 \| 0.799 \| \| ConsMed \| se_automedica \| 0.399 \| 0.172 \| 2.323 \| 0.020 \| 0.290 \| \| ConsNat \| mnt \| 1.000 \| 0.000 \| NA \| NA \| 0.359 \| \| ConsNat \| suplemento \| 3.429 \| 0.910 \| 3.769 \| 0.000 \| 1.232 \| \| ConsNat \| suplem_vitam \| 2.249 \| 0.477 \| 4.715 \| 0.000 \| 0.809 \| \| ConsNat \| suplem_nutric \| 1.601 \| 0.405 \| 3.952 \| 0.000 \| 0.575 \| \| ConsPsic \| mas_4_tazas_cafe \| 1.000 \| 0.000 \| NA \| NA \| 0.722 \| \| ConsPsic \| fuma \| 0.192 \| 0.086 \| 2.229 \| 0.026 \| 0.138 \| \| ConsPsic \| fuma_m10cdia \| 0.435 \| 0.103 \| 4.215 \| 0.000 \| 0.314 \| \| ConsPsic \| permite_fumen \| 0.828 \| 0.060 \| 13.876 \| 0.000 \| 0.597 \| \| ConsPsic \| cons_psicofarmaco2 \| 0.117 \| 0.086 \| 1.364 \| 0.172 \| 0.085 \| \| ConsPsic \| cons_sust_psicoact \| 1.262 \| 0.459 \| 2.752 \| 0.006 \| 0.911 \| \| EconDepr \| fuente_agua_acued \| 1.000 \| 0.000 \| NA \| NA \| 0.972 \| \| EconDepr \| fuente_agua_pozo \| -0.929 \| 0.056 \| -16.606 \| 0.000 \| -0.903 \| \| EconDepr \| metodo_desinf_agua \| 0.332 \| 0.071 \| 4.692 \| 0.000 \| 0.323 \| \| EconDepr \| met_desinf_hervir \| 0.636 \| 0.060 \| 10.599 \| 0.000 \| 0.618 \| \| EconDepr \| frecuenca_agua_leq1vsem \| -0.546 \| 0.092 \| -5.924 \| 0.000 \| -0.531 \| \| EconDepr \| hacinamiento_gt2 \| 0.272 \| 0.105 \| 2.581 \| 0.010 \| 0.264 \| \| EconDepr \| gastos_fam_lt50cuc \| -0.659 \| 0.057 \| -11.637 \| 0.000 \| -0.640 \| \| EconDepr \| gastos_fam_lt400mn \| -0.428 \| 0.144 \| -2.971 \| 0.003 \| -0.416 \| \| EconDepr \| equipos_viv_auto_moto \| 0.614 \| 0.059 \| 10.396 \| 0.000 \| 0.597 \| \| EconDepr \| equipos_viv_aireac \| 0.832 \| 0.042 \| 19.613 \| 0.000 \| 0.808 \| \| EconDepr \| equipos_viv_microwav \| 0.856 \| 0.043 \| 19.897 \| 0.000 \| 0.832 \| \| EconDepr \| equipos_viv_comput \| 0.649 \| 0.052 \| 12.480 \| 0.000 \| 0.631 \| \| EconDepr \| equipos_viv_tv_plana \| 1.043 \| 0.039 \| 26.526 \| 0.000 \| 1.014 \| \| EconDepr \| equipos_viv_tv \| -0.938 \| 0.038 \| -24.544 \| 0.000 \| -0.912 \| \| EconDepr \| equipos_viv_bicicleta \| -0.252 \| 0.075 \| -3.382 \| 0.001 \| -0.245 \| \| EconDepr \| equipos_viv_refrig \| 0.041 \| 0.091 \| 0.447 \| 0.655 \| 0.039 \| \| EconDepr \| equipos_viv_lavadora \| 0.387 \| 0.043 \| 9.072 \| 0.000 \| 0.377 \| \| EconDepr \| equipos_viv_ventilador \| 0.034 \| 0.093 \| 0.371 \| 0.711 \| 0.033 \| \| EconDepr \| equipos_viv_batidora \| 0.496 \| 0.148 \| 3.342 \| 0.001 \| 0.482 \| \| EconDepr \| equipos_viv_cocina_gas \| 0.439 \| 0.088 \| 4.985 \| 0.000 \| 0.427 \| \| freetime \| t_libre_semana \| 1.000 \| 0.000 \| NA \| NA \| 0.865 \| \| freetime \| t_libre_dia \| 0.806 \| 0.106 \| 7.621 \| 0.000 \| 0.697 \| \| freetime \| pref_cine \| 0.814 \| 0.146 \| 5.590 \| 0.000 \| 0.704 \| \| freetime \| pref_musica \| 0.627 \| 0.091 \| 6.899 \| 0.000 \| 0.542 \| \| freetime \| pref_pasear \| 0.596 \| 0.101 \| 5.891 \| 0.000 \| 0.515 \| \| freetime \| pref_tv \| 0.165 \| 0.097 \| 1.707 \| 0.088 \| 0.143 \| \| freetime \| pref_ami_fami \| 0.920 \| 0.086 \| 10.635 \| 0.000 \| 0.795 \| \| freetime \| pref_estudiar \| 0.898 \| 0.104 \| 8.611 \| 0.000 \| 0.776 \| \| freetime \| pref_deporte \| 0.584 \| 0.112 \| 5.197 \| 0.000 \| 0.505 \| \| freetime \| pref_leer \| 0.616 \| 0.095 \| 6.510 \| 0.000 \| 0.533 \| \| freetime \| pref_nada \| 0.079 \| 0.153 \| 0.520 \| 0.603 \| 0.069 \| \| freetime \| pref_jugaconnh \| 0.572 \| 0.110 \| 5.206 \| 0.000 \| 0.495 \| \| Pollut \| contam_ambiental \| 1.000 \| 0.000 \| NA \| NA \| 1.025 \| \| Pollut \| contam_amb_dessol \| 0.732 \| 0.065 \| 11.275 \| 0.000 \| 0.750 \| \| Pollut \| contam_amb_desconst \| 0.776 \| 0.064 \| 12.073 \| 0.000 \| 0.795 \| \| Pollut \| contam_amb_traficvh \| 0.542 \| 0.096 \| 5.654 \| 0.000 \| 0.555 \| \| Pollut \| contam_amb_quimindust \| 0.716 \| 0.065 \| 11.072 \| 0.000 \| 0.734 \| \| Pollut \| contam_amb_mucho \| 0.792 \| 0.062 \| 12.836 \| 0.000 \| 0.812 \| \| Pollut \| usa_quimicos_casa \| 0.063 \| 0.091 \| 0.688 \| 0.491 \| 0.064 \| \| Pollut \| cambio_donde_vives \| 0.860 \| 0.066 \| 13.042 \| 0.000 \| 0.881 \| \| Pollut \| cambio_nuevas_construcc \| 0.402 \| 0.116 \| 3.473 \| 0.001 \| 0.412 \| \| Pollut \| cambio_nuevas_fabricas \| 0.642 \| 0.061 \| 10.453 \| 0.000 \| 0.658 \| \| Pollut \| cambio_mov_personas \| 0.700 \| 0.070 \| 10.064 \| 0.000 \| 0.717 \| \| Pollut \| cambio_aumento_trafico_veh \| 0.702 \| 0.059 \| 11.908 \| 0.000 \| 0.719 \| \| selfcare \| ac_estomat1va \| 1.000 \| 0.000 \| NA \| NA \| 0.791 \| \| selfcare \| ac_med1va \| 1.131 \| 0.063 \| 17.825 \| 0.000 \| 0.895 \| \| selfcare \| ac_evita_sol \| 0.360 \| 0.098 \| 3.670 \| 0.000 \| 0.285 \| \| selfcare \| ac_usa_prot_solar \| 0.209 \| 0.100 \| 2.092 \| 0.036 \| 0.166 \| \| selfcare \| cheq_ta_1va \| 1.098 \| 0.067 \| 16.419 \| 0.000 \| 0.869 \| \| selfcare \| cheq_tricol_1va \| 1.253 \| 0.067 \| 18.738 \| 0.000 \| 0.992 \| \| selfcare \| cheq_glic_1va \| 1.237 \| 0.066 \| 18.777 \| 0.000 \| 0.979 \| \| selfcare \| cheq_obs_cuerpo \| 0.998 \| 0.071 \| 14.013 \| 0.000 \| 0.790 \| \| selfcare \| cheq_detect_precoz_cancer \| 0.611 \| 0.095 \| 6.464 \| 0.000 \| 0.484 \| \| selfcare \| aborda_veh_chof_alcohol \| 0.212 \| 0.224 \| 0.944 \| 0.345 \| 0.167 \| \| selfcare \| obed_ley_trans \| 0.452 \| 0.210 \| 2.151 \| 0.032 \| 0.358 \| \| selfcare \| usa_cint_seguridad \| 0.583 \| 0.120 \| 4.849 \| 0.000 \| 0.461 \| \| Sleep \| sleep_inadeq_time \| 1.000 \| 0.000 \| NA \| NA \| 0.145 \| \| Sleep \| levanta_descansado \| 5.738 \| 3.490 \| 1.644 \| 0.100 \| 0.834 \| \| Sleep \| dificul_dormir \| 6.190 \| 3.766 \| 1.643 \| 0.100 \| 0.900 \| \| Sleep \| despierta_multiplesv \| 5.951 \| 3.615 \| 1.646 \| 0.100 \| 0.865 \| \| Sleep \| sueno_durante_dia \| 5.038 \| 3.090 \| 1.630 \| 0.103 \| 0.732 \| \| Sleep \| usa_pastillas_dormir \| 4.286 \| 2.655 \| 1.614 \| 0.106 \| 0.623 \| \| Stress \| clasif_trabajo \| 1.000 \| 0.000 \| NA \| NA \| 0.424 \| \| Stress \| disfruta_trabajo \| 0.844 \| 0.407 \| 2.075 \| 0.038 \| 0.358 \| \| Stress \| amb_trabajo \| 1.552 \| 0.428 \| 3.626 \| 0.000 \| 0.658 \| \| Stress \| stress_casa \| 1.317 \| 0.382 \| 3.451 \| 0.001 \| 0.558 \| \| Stress \| stress_casa_padres \| 2.221 \| 0.550 \| 4.040 \| 0.000 \| 0.942 \| \| Stress \| violenc_verb_fis_xpadrs \| 2.290 \| 0.539 \| 4.245 \| 0.000 \| 0.971 \| \| Stress \| violenc_entorno \| 0.915 \| 0.427 \| 2.141 \| 0.032 \| 0.388 \| \| Workenv \| horas_trabajo \| 1.000 \| 0.000 \| NA \| NA \| 1.023 \| \| Workenv \| ubicac_lug_trabajo \| 0.224 \| 0.078 \| 2.880 \| 0.004 \| 0.229 \| \| Workenv \| act_fisica_trabajo \| 0.649 \| 0.083 \| 7.775 \| 0.000 \| 0.664 \| \| Workenv \| descanso_en_trabajo \| -0.666 \| 0.126 \| -5.306 \| 0.000 \| -0.682 \| \| Workenv \| medio_protec \| -0.653 \| 0.101 \| -6.472 \| 0.000 \| -0.668 \| \| Workenv \| antec_accid_laboral \| 0.237 \| 0.093 \| 2.560 \| 0.010 \| 0.243 \| |
| --- | --- | --- | --- | --- | --- | --- | --- | --- | --- | --- | --- | --- | --- | --- | --- | --- | --- | --- | --- | --- | --- | --- | --- | --- | --- | --- | --- | --- | --- | --- | --- | --- | --- | --- | --- | --- | --- | --- | --- | --- | --- | --- | --- | --- | --- | --- | --- | --- | --- | --- | --- | --- | --- | --- | --- | --- | --- | --- | --- | --- | --- | --- | --- | --- | --- | --- | --- | --- | --- | --- | --- | --- | --- | --- | --- | --- | --- | --- | --- | --- | --- | --- | --- | --- | --- | --- | --- | --- | --- | --- | --- | --- | --- | --- | --- | --- | --- | --- | --- | --- | --- | --- | --- | --- | --- | --- | --- | --- | --- | --- | --- | --- | --- | --- | --- | --- | --- | --- | --- | --- | --- | --- | --- | --- | --- | --- | --- | --- | --- | --- | --- | --- | --- | --- | --- | --- | --- | --- | --- | --- | --- | --- | --- | --- | --- | --- | --- | --- | --- | --- | --- | --- | --- | --- | --- | --- | --- | --- | --- | --- | --- | --- | --- | --- | --- | --- | --- | --- | --- | --- | --- | --- | --- | --- | --- | --- | --- | --- | --- | --- | --- | --- | --- | --- | --- | --- | --- | --- | --- | --- | --- | --- | --- | --- | --- | --- | --- | --- | --- | --- | --- | --- | --- | --- | --- | --- | --- | --- | --- | --- | --- | --- | --- | --- | --- | --- | --- | --- | --- | --- | --- | --- | --- | --- | --- | --- | --- | --- | --- | --- | --- | --- | --- | --- | --- | --- | --- | --- | --- | --- | --- | --- | --- | --- | --- | --- | --- | --- | --- | --- | --- | --- | --- | --- | --- | --- | --- | --- | --- | --- | --- | --- | --- | --- | --- | --- | --- | --- | --- | --- | --- | --- | --- | --- | --- | --- | --- | --- | --- | --- | --- | --- | --- | --- | --- | --- | --- | --- | --- | --- | --- | --- | --- | --- | --- | --- | --- | --- | --- | --- | --- | --- | --- | --- | --- | --- | --- | --- | --- | --- | --- | --- | --- | --- | --- | --- | --- | --- | --- | --- | --- | --- | --- | --- | --- | --- | --- | --- | --- | --- | --- | --- | --- | --- | --- | --- | --- | --- | --- | --- | --- | --- | --- | --- | --- | --- | --- | --- | --- | --- | --- | --- | --- | --- | --- | --- | --- | --- | --- | --- | --- | --- | --- | --- | --- | --- | --- | --- | --- | --- | --- | --- | --- | --- | --- | --- | --- | --- | --- | --- | --- | --- | --- | --- | --- | --- | --- | --- | --- | --- | --- | --- | --- | --- | --- | --- | --- | --- | --- | --- | --- | --- | --- | --- | --- | --- | --- | --- | --- | --- | --- | --- | --- | --- | --- | --- | --- | --- | --- | --- | --- | --- | --- | --- | --- | --- | --- | --- | --- | --- | --- | --- | --- | --- | --- | --- | --- | --- | --- | --- | --- | --- | --- | --- | --- | --- | --- | --- | --- | --- | --- | --- | --- | --- | --- | --- | --- | --- | --- | --- | --- | --- | --- | --- | --- | --- | --- | --- | --- | --- | --- | --- | --- | --- | --- | --- | --- | --- | --- | --- | --- | --- | --- | --- | --- | --- | --- | --- | --- | --- | --- | --- | --- | --- | --- | --- | --- | --- | --- | --- | --- | --- | --- | --- | --- | --- | --- | --- | --- | --- | --- | --- | --- | --- | --- | --- | --- | --- | --- | --- | --- | --- | --- | --- | --- | --- | --- | --- | --- | --- | --- | --- | --- | --- | --- | --- | --- | --- | --- | --- | --- | --- | --- | --- | --- | --- | --- | --- | --- | --- | --- | --- | --- | --- | --- | --- | --- | --- | --- | --- | --- | --- | --- | --- | --- | --- | --- | --- | --- | --- | --- | --- | --- | --- | --- | --- | --- | --- | --- | --- | --- | --- | --- | --- | --- | --- | --- | --- | --- | --- | --- | --- | --- | --- | --- | --- | --- | --- | --- | --- | --- | --- | --- | --- | --- | --- | --- | --- | --- | --- | --- | --- | --- | --- | --- | --- | --- | --- | --- | --- | --- | --- | --- | --- | --- | --- | --- | --- | --- | --- | --- | --- | --- | --- | --- | --- | --- | --- | --- | --- | --- | --- | --- | --- | --- | --- | --- | --- | --- | --- | --- | --- | --- | --- | --- | --- | --- | --- | --- | --- | --- | --- | --- | --- | --- | --- | --- | --- | --- | --- | --- | --- | --- | --- | --- | --- | --- | --- | --- | --- | --- | --- | --- | --- | --- | --- | --- | --- | --- | --- | --- | --- | --- | --- | --- | --- | --- | --- | --- | --- | --- | --- | --- | --- | --- | --- | --- | --- | --- | --- | --- | --- | --- | --- | --- | --- | --- | --- | --- | --- | --- | --- | --- | --- | --- | --- | --- | --- | --- | --- | --- | --- | --- | --- | --- | --- | --- | --- | --- | --- | --- | --- | --- | --- | --- | --- | --- | --- | --- | --- | --- | --- | --- | --- | --- | --- | --- | --- | --- | --- | --- | --- | --- | --- | --- | --- | --- | --- | --- | --- | --- | --- | --- | --- | --- | --- | --- | --- | --- | --- | --- | --- | --- | --- | --- | --- | --- | --- | --- | --- | --- | --- | --- | --- | --- | --- | --- | --- | --- | --- | --- | --- | --- | --- | --- | --- | --- | --- | --- | --- | --- | --- | --- | --- | --- | --- | --- | --- | --- | --- | --- | --- | --- | --- | --- | --- | --- | --- | --- | --- | --- | --- | --- | --- | --- | --- | --- | --- | --- | --- | --- | --- | --- | --- | --- | --- | --- | --- | --- | --- | --- | --- | --- | --- | --- | --- | --- | --- | --- | --- | --- | --- | --- | --- | --- | --- | --- | --- | --- | --- | --- | --- | --- | --- | --- | --- | --- | --- | --- | --- | --- | --- | --- | --- | --- | --- | --- | --- | --- | --- | --- | --- | --- | --- | --- | --- | --- | --- | --- | --- | --- | --- | --- |

| Table S4.5: Item standardized variances by model.   \| model \| indicator \| B \| SE \| Z \| p-value \| Beta \| \| --- \| --- \| --- \| --- \| --- \| --- \| --- \| \| Diet \| comid_horario_regul \| 0.748 \| 0.000 \| NA \| NA \| 0.748 \| \| Diet \| desayuna_antes \| 0.142 \| 0.000 \| NA \| NA \| 0.142 \| \| Diet \| desayuno \| 0.005 \| 0.000 \| NA \| NA \| 0.005 \| \| Diet \| almuerzo \| 0.836 \| 0.000 \| NA \| NA \| 0.836 \| \| Diet \| cena \| 0.696 \| 0.000 \| NA \| NA \| 0.696 \| \| Diet \| merienda_manana \| 0.679 \| 0.000 \| NA \| NA \| 0.679 \| \| Diet \| merienda_tarde \| 0.572 \| 0.000 \| NA \| NA \| 0.572 \| \| Diet \| merienda_desp_cena \| 0.641 \| 0.000 \| NA \| NA \| 0.641 \| \| Diet \| toma_cafe \| 0.361 \| 0.000 \| NA \| NA \| 0.361 \| \| Diet \| cafe_con_azucar \| 0.361 \| 0.000 \| NA \| NA \| 0.361 \| \| Diet \| condim_sal \| 0.581 \| 0.000 \| NA \| NA \| 0.581 \| \| Diet \| dieta_p_perd_peso \| 0.979 \| 0.000 \| NA \| NA \| 0.979 \| \| Diet \| cantid_agua_toma_dia_menos4 \| 0.976 \| 0.000 \| NA \| NA \| 0.976 \| \| Diet \| com_rapida_m3vps \| 0.998 \| 0.000 \| NA \| NA \| 0.998 \| \| Diet \| mucha_sal \| 1.000 \| 0.000 \| NA \| NA \| 1.000 \| \| Diet \| ahumados_m1vps \| 0.971 \| 0.000 \| NA \| NA \| 0.971 \| \| Diet \| cons_refr_enlat \| 0.999 \| 0.000 \| NA \| NA \| 0.999 \| \| Diet \| cons_dulces_helados \| 0.980 \| 0.000 \| NA \| NA \| 0.980 \| \| Diet \| alim_dulces \| 0.988 \| 0.000 \| NA \| NA \| 0.988 \| \| Diet \| tipo_carne_res \| 0.779 \| 0.000 \| NA \| NA \| 0.779 \| \| Diet \| tipo_carne_carnero \| 0.910 \| 0.000 \| NA \| NA \| 0.910 \| \| Diet \| tipo_carne_cerdo \| 0.983 \| 0.000 \| NA \| NA \| 0.983 \| \| Diet \| tipo_carne_pollo \| 0.949 \| 0.000 \| NA \| NA \| 0.949 \| \| Diet \| tipo_carne_pescado \| 0.999 \| 0.000 \| NA \| NA \| 0.999 \| \| Diet \| x1vs_lacteos \| 0.873 \| 0.000 \| NA \| NA \| 0.873 \| \| Diet \| x1vs_vegetales \| 0.803 \| 0.000 \| NA \| NA \| 0.803 \| \| Diet \| x1vs_frutas \| 0.722 \| 0.000 \| NA \| NA \| 0.722 \| \| Diet \| x1vs_granos \| 0.645 \| 0.000 \| NA \| NA \| 0.645 \| \| Diet \| x1vs_panes \| 0.974 \| 0.000 \| NA \| NA \| 0.974 \| \| Diet \| x1vs_cereales \| 0.858 \| 0.000 \| NA \| NA \| 0.858 \| \| Diet \| condim_naturales \| 0.555 \| 0.000 \| NA \| NA \| 0.555 \| \| Diet \| condim_deshidratado \| 0.991 \| 0.000 \| NA \| NA \| 0.991 \| \| Diet \| condim_procesados \| 0.999 \| 0.000 \| NA \| NA \| 0.999 \| \| Diet \| Diet \| 0.252 \| 0.053 \| 4.780 \| 0.000 \| 1.000 \| \| Physcal \| frec_ej_fisico \| 0.139 \| 0.000 \| NA \| NA \| 0.139 \| \| Physcal \| frec_ej_fisico_cardio \| 0.093 \| 0.000 \| NA \| NA \| 0.093 \| \| Physcal \| frec_ej_fisico_relaj \| 0.294 \| 0.000 \| NA \| NA \| 0.294 \| \| Physcal \| ej_fis_supervis_entrenador \| 0.234 \| 0.000 \| NA \| NA \| 0.234 \| \| Physcal \| ej_fis_supervis_fisiot \| 0.711 \| 0.000 \| NA \| NA \| 0.711 \| \| Physcal \| phys_act \| 0.861 \| 0.072 \| 11.949 \| 0.000 \| 1.000 \| \| Sedent \| horas_sentado \| 0.370 \| 0.000 \| NA \| NA \| 0.370 \| \| Sedent \| cuanto_descanso \| 0.370 \| 0.000 \| NA \| NA \| 0.370 \| \| Sedent \| sedentary \| 0.630 \| 0.115 \| 5.482 \| 0.000 \| 1.000 \| \| ConsMeds \| toma_medicam \| 0.470 \| 0.000 \| NA \| NA \| 0.470 \| \| ConsMeds \| muchos_medicam \| 0.362 \| 0.000 \| NA \| NA \| 0.362 \| \| ConsMeds \| se_automedica \| 0.916 \| 0.000 \| NA \| NA \| 0.916 \| \| ConsMeds \| ConsMed \| 0.530 \| 0.316 \| 1.680 \| 0.093 \| 1.000 \| \| ConsNat \| mnt \| 0.871 \| 0.000 \| NA \| NA \| 0.871 \| \| ConsNat \| suplemento \| -0.519 \| 0.000 \| NA \| NA \| -0.519 \| \| ConsNat \| suplem_vitam \| 0.346 \| 0.000 \| NA \| NA \| 0.346 \| \| ConsNat \| suplem_nutric \| 0.669 \| 0.000 \| NA \| NA \| 0.669 \| \| ConsNat \| ConsNat \| 0.129 \| 0.058 \| 2.236 \| 0.025 \| 1.000 \| \| ConsPsyc \| mas_4_tazas_cafe \| 0.479 \| 0.000 \| NA \| NA \| 0.479 \| \| ConsPsyc \| fuma \| 0.981 \| 0.000 \| NA \| NA \| 0.981 \| \| ConsPsyc \| fuma_m10cdia \| 0.901 \| 0.000 \| NA \| NA \| 0.901 \| \| ConsPsyc \| permite_fumen \| 0.643 \| 0.000 \| NA \| NA \| 0.643 \| \| ConsPsyc \| cons_psicofarmaco2 \| 0.993 \| 0.000 \| NA \| NA \| 0.993 \| \| ConsPsyc \| cons_sust_psicoact \| 0.170 \| 0.000 \| NA \| NA \| 0.170 \| \| ConsPsyc \| ConsPsic \| 0.521 \| 0.192 \| 2.714 \| 0.007 \| 1.000 \| \| Econ \| fuente_agua_acued \| 0.055 \| 0.000 \| NA \| NA \| 0.055 \| \| Econ \| fuente_agua_pozo \| 0.185 \| 0.000 \| NA \| NA \| 0.185 \| \| Econ \| metodo_desinf_agua \| 0.896 \| 0.000 \| NA \| NA \| 0.896 \| \| Econ \| met_desinf_hervir \| 0.618 \| 0.000 \| NA \| NA \| 0.618 \| \| Econ \| frecuenca_agua_leq1vsem \| 0.718 \| 0.000 \| NA \| NA \| 0.718 \| \| Econ \| hacinamiento_gt2 \| 0.930 \| 0.000 \| NA \| NA \| 0.930 \| \| Econ \| gastos_fam_lt50cuc \| 0.590 \| 0.000 \| NA \| NA \| 0.590 \| \| Econ \| gastos_fam_lt400mn \| 0.827 \| 0.000 \| NA \| NA \| 0.827 \| \| Econ \| equipos_viv_auto_moto \| 0.644 \| 0.000 \| NA \| NA \| 0.644 \| \| Econ \| equipos_viv_aireac \| 0.346 \| 0.000 \| NA \| NA \| 0.346 \| \| Econ \| equipos_viv_microwav \| 0.308 \| 0.000 \| NA \| NA \| 0.308 \| \| Econ \| equipos_viv_comput \| 0.602 \| 0.000 \| NA \| NA \| 0.602 \| \| Econ \| equipos_viv_tv_plana \| -0.029 \| 0.000 \| NA \| NA \| -0.029 \| \| Econ \| equipos_viv_tv \| 0.169 \| 0.000 \| NA \| NA \| 0.169 \| \| Econ \| equipos_viv_bicicleta \| 0.940 \| 0.000 \| NA \| NA \| 0.940 \| \| Econ \| equipos_viv_refrig \| 0.998 \| 0.000 \| NA \| NA \| 0.998 \| \| Econ \| equipos_viv_lavadora \| 0.858 \| 0.000 \| NA \| NA \| 0.858 \| \| Econ \| equipos_viv_ventilador \| 0.999 \| 0.000 \| NA \| NA \| 0.999 \| \| Econ \| equipos_viv_batidora \| 0.768 \| 0.000 \| NA \| NA \| 0.768 \| \| Econ \| equipos_viv_cocina_gas \| 0.818 \| 0.000 \| NA \| NA \| 0.818 \| \| Econ \| EconDepr \| 0.945 \| 0.059 \| 16.076 \| 0.000 \| 1.000 \| \| Freetime \| t_libre_semana \| 0.252 \| 0.000 \| NA \| NA \| 0.252 \| \| Freetime \| t_libre_dia \| 0.514 \| 0.000 \| NA \| NA \| 0.514 \| \| Freetime \| pref_cine \| 0.504 \| 0.000 \| NA \| NA \| 0.504 \| \| Freetime \| pref_musica \| 0.706 \| 0.000 \| NA \| NA \| 0.706 \| \| Freetime \| pref_pasear \| 0.734 \| 0.000 \| NA \| NA \| 0.734 \| \| Freetime \| pref_tv \| 0.980 \| 0.000 \| NA \| NA \| 0.980 \| \| Freetime \| pref_ami_fami \| 0.368 \| 0.000 \| NA \| NA \| 0.368 \| \| Freetime \| pref_estudiar \| 0.398 \| 0.000 \| NA \| NA \| 0.398 \| \| Freetime \| pref_deporte \| 0.745 \| 0.000 \| NA \| NA \| 0.745 \| \| Freetime \| pref_leer \| 0.716 \| 0.000 \| NA \| NA \| 0.716 \| \| Freetime \| pref_nada \| 0.995 \| 0.000 \| NA \| NA \| 0.995 \| \| Freetime \| pref_jugaconnh \| 0.755 \| 0.000 \| NA \| NA \| 0.755 \| \| Freetime \| freetime \| 0.748 \| 0.115 \| 6.530 \| 0.000 \| 1.000 \| \| Pollut \| contam_ambiental \| -0.050 \| 0.000 \| NA \| NA \| -0.050 \| \| Pollut \| contam_amb_dessol \| 0.437 \| 0.000 \| NA \| NA \| 0.437 \| \| Pollut \| contam_amb_desconst \| 0.367 \| 0.000 \| NA \| NA \| 0.367 \| \| Pollut \| contam_amb_traficvh \| 0.692 \| 0.000 \| NA \| NA \| 0.692 \| \| Pollut \| contam_amb_quimindust \| 0.461 \| 0.000 \| NA \| NA \| 0.461 \| \| Pollut \| contam_amb_mucho \| 0.341 \| 0.000 \| NA \| NA \| 0.341 \| \| Pollut \| usa_quimicos_casa \| 0.996 \| 0.000 \| NA \| NA \| 0.996 \| \| Pollut \| cambio_donde_vives \| 0.223 \| 0.000 \| NA \| NA \| 0.223 \| \| Pollut \| cambio_nuevas_construcc \| 0.830 \| 0.000 \| NA \| NA \| 0.830 \| \| Pollut \| cambio_nuevas_fabricas \| 0.566 \| 0.000 \| NA \| NA \| 0.566 \| \| Pollut \| cambio_mov_personas \| 0.486 \| 0.000 \| NA \| NA \| 0.486 \| \| Pollut \| cambio_aumento_trafico_veh \| 0.482 \| 0.000 \| NA \| NA \| 0.482 \| \| Pollut \| Pollut \| 1.050 \| 0.093 \| 11.335 \| 0.000 \| 1.000 \| \| Selfcare \| ac_estomat1va \| 0.374 \| 0.000 \| NA \| NA \| 0.374 \| \| Selfcare \| ac_med1va \| 0.199 \| 0.000 \| NA \| NA \| 0.199 \| \| Selfcare \| ac_evita_sol \| 0.919 \| 0.000 \| NA \| NA \| 0.919 \| \| Selfcare \| ac_usa_prot_solar \| 0.973 \| 0.000 \| NA \| NA \| 0.973 \| \| Selfcare \| cheq_ta_1va \| 0.244 \| 0.000 \| NA \| NA \| 0.244 \| \| Selfcare \| cheq_tricol_1va \| 0.016 \| 0.000 \| NA \| NA \| 0.016 \| \| Selfcare \| cheq_glic_1va \| 0.041 \| 0.000 \| NA \| NA \| 0.041 \| \| Selfcare \| cheq_obs_cuerpo \| 0.377 \| 0.000 \| NA \| NA \| 0.377 \| \| Selfcare \| cheq_detect_precoz_cancer \| 0.766 \| 0.000 \| NA \| NA \| 0.766 \| \| Selfcare \| aborda_veh_chof_alcohol \| 0.972 \| 0.000 \| NA \| NA \| 0.972 \| \| Selfcare \| obed_ley_trans \| 0.872 \| 0.000 \| NA \| NA \| 0.872 \| \| Selfcare \| usa_cint_seguridad \| 0.787 \| 0.000 \| NA \| NA \| 0.787 \| \| Selfcare \| selfcare \| 0.626 \| 0.065 \| 9.566 \| 0.000 \| 1.000 \| \| Sleep \| sleep_inadeq_time \| 0.979 \| 0.000 \| NA \| NA \| 0.979 \| \| Sleep \| levanta_descansado \| 0.305 \| 0.000 \| NA \| NA \| 0.305 \| \| Sleep \| dificul_dormir \| 0.191 \| 0.000 \| NA \| NA \| 0.191 \| \| Sleep \| despierta_multiplesv \| 0.252 \| 0.000 \| NA \| NA \| 0.252 \| \| Sleep \| sueno_durante_dia \| 0.464 \| 0.000 \| NA \| NA \| 0.464 \| \| Sleep \| usa_pastillas_dormir \| 0.612 \| 0.000 \| NA \| NA \| 0.612 \| \| Sleep \| Sleep \| 0.021 \| 0.026 \| 0.823 \| 0.410 \| 1.000 \| \| Stress \| clasif_trabajo \| 0.820 \| 0.000 \| NA \| NA \| 0.820 \| \| Stress \| disfruta_trabajo \| 0.872 \| 0.000 \| NA \| NA \| 0.872 \| \| Stress \| amb_trabajo \| 0.567 \| 0.000 \| NA \| NA \| 0.567 \| \| Stress \| stress_casa \| 0.688 \| 0.000 \| NA \| NA \| 0.688 \| \| Stress \| stress_casa_padres \| 0.113 \| 0.000 \| NA \| NA \| 0.113 \| \| Stress \| violenc_verb_fis_xpadrs \| 0.057 \| 0.000 \| NA \| NA \| 0.057 \| \| Stress \| violenc_entorno \| 0.850 \| 0.000 \| NA \| NA \| 0.850 \| \| Stress \| Stress \| 0.180 \| 0.084 \| 2.147 \| 0.032 \| 1.000 \| \| WorkEnv \| horas_trabajo \| -0.048 \| 0.000 \| NA \| NA \| -0.048 \| \| WorkEnv \| ubicac_lug_trabajo \| 0.947 \| 0.000 \| NA \| NA \| 0.947 \| \| WorkEnv \| act_fisica_trabajo \| 0.559 \| 0.000 \| NA \| NA \| 0.559 \| \| WorkEnv \| descanso_en_trabajo \| 0.535 \| 0.000 \| NA \| NA \| 0.535 \| \| WorkEnv \| medio_protec \| 0.553 \| 0.000 \| NA \| NA \| 0.553 \| \| WorkEnv \| antec_accid_laboral \| 0.941 \| 0.000 \| NA \| NA \| 0.941 \| \| WorkEnv \| Workenv \| 1.048 \| 0.190 \| 5.527 \| 0.000 \| 1.000 \| |
| --- | --- | --- | --- | --- | --- | --- | --- | --- | --- | --- | --- | --- | --- | --- | --- | --- | --- | --- | --- | --- | --- | --- | --- | --- | --- | --- | --- | --- | --- | --- | --- | --- | --- | --- | --- | --- | --- | --- | --- | --- | --- | --- | --- | --- | --- | --- | --- | --- | --- | --- | --- | --- | --- | --- | --- | --- | --- | --- | --- | --- | --- | --- | --- | --- | --- | --- | --- | --- | --- | --- | --- | --- | --- | --- | --- | --- | --- | --- | --- | --- | --- | --- | --- | --- | --- | --- | --- | --- | --- | --- | --- | --- | --- | --- | --- | --- | --- | --- | --- | --- | --- | --- | --- | --- | --- | --- | --- | --- | --- | --- | --- | --- | --- | --- | --- | --- | --- | --- | --- | --- | --- | --- | --- | --- | --- | --- | --- | --- | --- | --- | --- | --- | --- | --- | --- | --- | --- | --- | --- | --- | --- | --- | --- | --- | --- | --- | --- | --- | --- | --- | --- | --- | --- | --- | --- | --- | --- | --- | --- | --- | --- | --- | --- | --- | --- | --- | --- | --- | --- | --- | --- | --- | --- | --- | --- | --- | --- | --- | --- | --- | --- | --- | --- | --- | --- | --- | --- | --- | --- | --- | --- | --- | --- | --- | --- | --- | --- | --- | --- | --- | --- | --- | --- | --- | --- | --- | --- | --- | --- | --- | --- | --- | --- | --- | --- | --- | --- | --- | --- | --- | --- | --- | --- | --- | --- | --- | --- | --- | --- | --- | --- | --- | --- | --- | --- | --- | --- | --- | --- | --- | --- | --- | --- | --- | --- | --- | --- | --- | --- | --- | --- | --- | --- | --- | --- | --- | --- | --- | --- | --- | --- | --- | --- | --- | --- | --- | --- | --- | --- | --- | --- | --- | --- | --- | --- | --- | --- | --- | --- | --- | --- | --- | --- | --- | --- | --- | --- | --- | --- | --- | --- | --- | --- | --- | --- | --- | --- | --- | --- | --- | --- | --- | --- | --- | --- | --- | --- | --- | --- | --- | --- | --- | --- | --- | --- | --- | --- | --- | --- | --- | --- | --- | --- | --- | --- | --- | --- | --- | --- | --- | --- | --- | --- | --- | --- | --- | --- | --- | --- | --- | --- | --- | --- | --- | --- | --- | --- | --- | --- | --- | --- | --- | --- | --- | --- | --- | --- | --- | --- | --- | --- | --- | --- | --- | --- | --- | --- | --- | --- | --- | --- | --- | --- | --- | --- | --- | --- | --- | --- | --- | --- | --- | --- | --- | --- | --- | --- | --- | --- | --- | --- | --- | --- | --- | --- | --- | --- | --- | --- | --- | --- | --- | --- | --- | --- | --- | --- | --- | --- | --- | --- | --- | --- | --- | --- | --- | --- | --- | --- | --- | --- | --- | --- | --- | --- | --- | --- | --- | --- | --- | --- | --- | --- | --- | --- | --- | --- | --- | --- | --- | --- | --- | --- | --- | --- | --- | --- | --- | --- | --- | --- | --- | --- | --- | --- | --- | --- | --- | --- | --- | --- | --- | --- | --- | --- | --- | --- | --- | --- | --- | --- | --- | --- | --- | --- | --- | --- | --- | --- | --- | --- | --- | --- | --- | --- | --- | --- | --- | --- | --- | --- | --- | --- | --- | --- | --- | --- | --- | --- | --- | --- | --- | --- | --- | --- | --- | --- | --- | --- | --- | --- | --- | --- | --- | --- | --- | --- | --- | --- | --- | --- | --- | --- | --- | --- | --- | --- | --- | --- | --- | --- | --- | --- | --- | --- | --- | --- | --- | --- | --- | --- | --- | --- | --- | --- | --- | --- | --- | --- | --- | --- | --- | --- | --- | --- | --- | --- | --- | --- | --- | --- | --- | --- | --- | --- | --- | --- | --- | --- | --- | --- | --- | --- | --- | --- | --- | --- | --- | --- | --- | --- | --- | --- | --- | --- | --- | --- | --- | --- | --- | --- | --- | --- | --- | --- | --- | --- | --- | --- | --- | --- | --- | --- | --- | --- | --- | --- | --- | --- | --- | --- | --- | --- | --- | --- | --- | --- | --- | --- | --- | --- | --- | --- | --- | --- | --- | --- | --- | --- | --- | --- | --- | --- | --- | --- | --- | --- | --- | --- | --- | --- | --- | --- | --- | --- | --- | --- | --- | --- | --- | --- | --- | --- | --- | --- | --- | --- | --- | --- | --- | --- | --- | --- | --- | --- | --- | --- | --- | --- | --- | --- | --- | --- | --- | --- | --- | --- | --- | --- | --- | --- | --- | --- | --- | --- | --- | --- | --- | --- | --- | --- | --- | --- | --- | --- | --- | --- | --- | --- | --- | --- | --- | --- | --- | --- | --- | --- | --- | --- | --- | --- | --- | --- | --- | --- | --- | --- | --- | --- | --- | --- | --- | --- | --- | --- | --- | --- | --- | --- | --- | --- | --- | --- | --- | --- | --- | --- | --- | --- | --- | --- | --- | --- | --- | --- | --- | --- | --- | --- | --- | --- | --- | --- | --- | --- | --- | --- | --- | --- | --- | --- | --- | --- | --- | --- | --- | --- | --- | --- | --- | --- | --- | --- | --- | --- | --- | --- | --- | --- | --- | --- | --- | --- | --- | --- | --- | --- | --- | --- | --- | --- | --- | --- | --- | --- | --- | --- | --- | --- | --- | --- | --- | --- | --- | --- | --- | --- | --- | --- | --- | --- | --- | --- | --- | --- | --- | --- | --- | --- | --- | --- | --- | --- | --- | --- | --- | --- | --- | --- | --- | --- | --- | --- | --- | --- | --- | --- | --- | --- | --- | --- | --- | --- | --- | --- | --- | --- | --- | --- | --- | --- | --- | --- | --- | --- | --- | --- | --- | --- | --- | --- | --- | --- | --- | --- | --- | --- | --- | --- | --- | --- | --- | --- | --- | --- | --- | --- | --- | --- | --- | --- | --- | --- | --- | --- | --- | --- | --- | --- | --- | --- | --- | --- | --- | --- | --- | --- | --- | --- | --- | --- | --- | --- | --- | --- | --- | --- | --- | --- | --- | --- | --- | --- | --- | --- | --- | --- | --- | --- | --- | --- | --- | --- | --- | --- | --- | --- | --- | --- | --- | --- | --- | --- | --- | --- | --- | --- | --- | --- | --- | --- | --- | --- | --- | --- | --- | --- | --- | --- | --- | --- | --- | --- | --- | --- | --- | --- | --- | --- | --- | --- | --- | --- | --- | --- | --- | --- | --- | --- | --- | --- | --- | --- | --- | --- | --- | --- | --- | --- | --- | --- | --- | --- | --- | --- | --- | --- | --- | --- | --- | --- | --- | --- | --- |
